# Long non-coding RNA lincRNA-erythroid prosurvival (EPS) alleviates cerebral ischemia/reperfusion injury by maintaining high-temperature requirement protein A1 (Htra1) stability through recruiting heterogeneous nuclear ribonucleoprotein L (HNRNPL)

**DOI:** 10.1080/21655979.2022.2074738

**Published:** 2022-05-13

**Authors:** Haifeng Guo, Xia Guo, Shiting Jiang

**Affiliations:** a Department of encephalopathy, Jinan Municipal Hospital of Traditional Chinese Medicine, Jinan, Shandong, P.R.China; b Department of Obstetrics, Dongying People’s Hospital, Dongying, Shandong, P.R.China; c Department of Internal Medicine-Neurology, Dongping People’s Hospital, Taian, Shandong, P.R.China

**Keywords:** Stroke, cerebral ischemia/reperfusion injury, lincRNA-EPS, HNRNPL, Htra1

## Abstract

This study aimed at investigating the role and mechanism of lincRNA-EPS (erythroid prosurvival) in cerebral ischemia/reperfusion (CIR) injury. The results showed that the overexpression of lincRNA-EPS was able to reduce the levels of interleukin-6, tumor necrosis factor-alpha and interleukin-1β stimulated in the OGD-treated Neuro-2a (N-2a) cells. The levels of reactive oxygen species and malondialdehyde were enhanced while the superoxide dismutase levels were reduced by oxygen and glucose deprivation (OGD) treatment, in which the lincRNA-EPS overexpression could reverse this effect in the cells. LincRNA-EPS interacted with high-temperature requirement protein A1 (Htra1) and heterogeneous nuclear ribonucleoprotein L (HNRNPL), and their depletion inhibited the Htra1 mRNA stability in N-2a cells. HNRNPL knockdown blocked lincRNA-EPS overexpression-induced Htra1 expression in the cells. The depletion of Htra1 could rescue lincRNA-EPS overexpression-mediated N-2a cell injury, inflammation, and oxidative stress induced by OGD. Functionally, lincRNA-EPS alleviates CIR injury of the middle cerebral artery occlusion/reperfusion mice *in vivo*. In conclusion, lincRNA-EPS attenuates CIR injury by maintaining Htra1 stability through recruiting HNRNPL.

## Graphical Abstract

**Figure:**
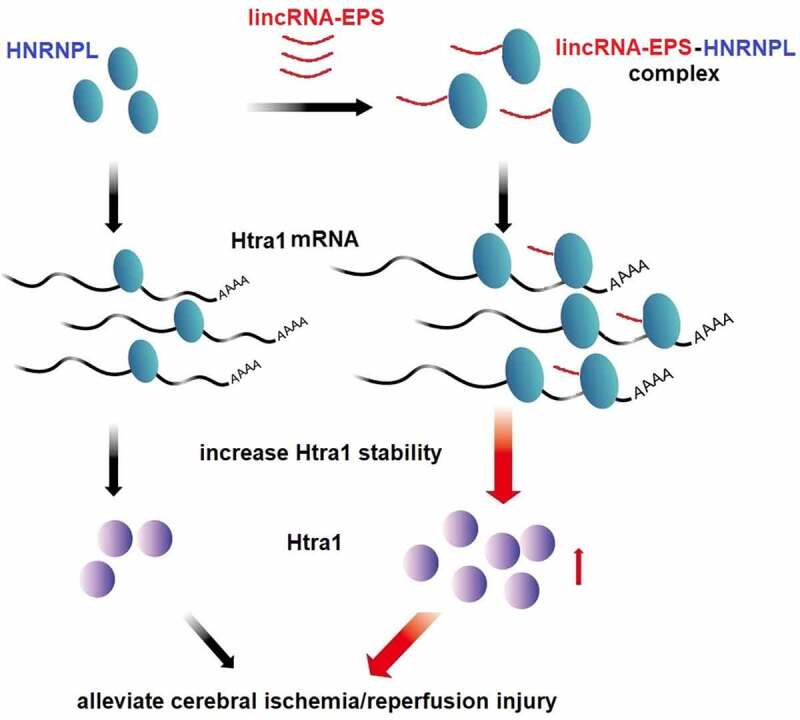

## Highlights

- LincRNA-EPS inhibits OGD-induced cell injury in N-2a cells
- LincRNA-EPS enhances stability of Htra1 by interacting with HNRNPL in N-2a cells
- LincRNA-EPS attenuates OGD-induced N-2a cell injury by maintaining Htra1 stability

## Introduction

Stroke is a leading cause of long-term physical disability and death globally [1,2]. Each year, approximately 15 million people suffer from stroke, and cerebral ischemia represents the cause in 87% of all stroke cases [3]. Stroke intrusions include the reconstruction of blood racecourse, which results in cerebral ischemia/reperfusion (CIR) injury [4]. CIR injury serves as a pathological process in which nervure lesions caused by hypoxia and ischemia are further developed following the short-course recovery of blood reperfusion [5]. Moreover, an increasing number of studies show that hypoxia and ischemia usually include a series of neural disorders, such as inflammatory response, apoptosis, and oxidative stress [6–8]. Hence, it is of great importance to understand the molecular mechanism of CIR injury.

Long non-coding RNAs (lncRNAs, ~200 nucleotides) represent a type of regulatory RNAs with no protein coding ability. They have been identified in regulating different cellular processes [9]. In recent years, lncRNAs have been reported to have an important role in the progression of CIR injury. For instance, through modulating the miR-136-5p/ROCK1 axis, lncRNA SNHG14 enhances CIR-induced inflammation [10]. LncRNA GAS5 promotes CIR injury by increasing neuronal glycolysis [11]. Furthermore, lncRNA lincRNA-EPS (erythroid prosurvival) is able to repress erythroid cell apoptosis [12] and has also been shown to act as a transcriptional brake to restrain inflammation [13]. Agliano et al. [14] reported that following infection with the intracellular bacterium *Listeria monocytogenes*, both mouse macrophages and dendritic cells that lack lincRNA-EPS increased the levels of inflammatory genes. A recent study by Zhang et al. has confirmed that targeting cerebral infarction, lincRNA-EPS inhibits inflammation and promotes neuron regeneration [15]. Nevertheless, the potential mechanisms behind the effects of lincRNA-EPS on CIR injury have rarely been studied.

LncRNAs are able to exert various functions through binding with miRNAs and interacting with RNA-binding proteins (RBPs) [16]. Heterogeneous nuclear ribonucleoproteins (HNRNPs) have been presented to have a major function in the modulation of alternative RNA splicing and to play fundamental roles in mRNA stabilization, translation, and transport as multi-functional RBPs [17]. There are more than 20 members in the HNRNPs family, termed from HNRNPA to HNRNPU [18]. HNRNPL has been identified to participate in multiple diseases, such as cancer and heart defects [19–22]. In addition, high-temperature requirement protein A1 (Htra1) is an evolutionarily conserved extracellular serine protease, which has been well-identified in brain disorders, including cerebral small vessel disease and cerebral autosomal recessive arteriopathy [23,24].

In this study, we investigated the role and underlying mechanism of lincRNA-EPS in CIR injury. Our study highlights the potential of lincRNA-EPS to act as an new therapeutic target in CIR injury by elevating the stability of Htra1 through recruiting HNRNPL.

## Materials and methods

### Middle cerebral artery occlusion/reperfusion (MCAO/R) mouse model

The middle cerebral artery occlusion/reperfusion (MCAO/R) mouse model was constructed in C57BL/6 J mice (six-week-old, male, 20–25 g) as previously described [25]. All mice were housed in a controlled environment with free access to food and water. In brief, the mice were anesthetized with an intraperitoneal injection of 3.5% chloral hydrate. Then, the left common carotid arteries of the mice were exposed, and a 4–0 monofilament nylon suture was inserted into the internal carotid artery and advanced to occlude the left middle cerebral artery. After 120 min, the monofilament was removed to perform the reperfusion. In the sham group, the mice underwent the same surgical procedures without monofilament insertion. At five days before MCAO, the control vector or pcDNA3.1-lincRNA-EPS was injected into the cortex of the mice at three points using a stereotaxia instrument under anesthesia as previously described [26]. The neurological function score was measured at 24 h after reperfusion. Then, brain tissues were collected for the subsequent experiments. Animal care and method procedure were authorized by the Animal Ethics Committee of Jinan Municipal Hospital of Traditional Chinese Medicine (DW20200112).

### 2,3,5-triphenyltetrazolium chloride (TTC) staining

TTC staining was used to assess cerebral infarction area [27]. The brain tissue was sliced into 2-mm-thick coronal sections. Subsequently, the sections were stained in 2% TTC (Sigma, USA) at 37°C for 30 min, and then fixed in 10% formaldehyde overnight. Finally, the Image J software was used to analyze the infarct volumes.

### Enzyme-linked immunosorbent assay (ELISA)

The expression of interleukin-6 (IL-6), tumor necrosis factor-alpha (TNF-α), and interleukin-1β (IL-1β) in the serum of the mice was analyzed using the ELISA. The samples were homogenized by phosphate-buffered saline (PBS) comprising protease-inhibitor (Sigma, USA), and the homogenate was centrifuged at 4°C for 30 min. The ELISA kits (TNF-α: no. PT512; IL-1β: no. PI301 and IL-6: no. PI326) were obtained from Beyotime, Jiangsu, China. All procedures were performed according to the manufacturer’s instructions.

### Cell culture and treatment

Mouse neuroblast cell line Neuro-2a (N-2a) were obtained from the Cell Bank of the Typical Culture Preservation Committee of the Chinese Academy of Sciences, and incubated at 5% CO_2_ and 37°C in the DMEM medium (Gibco, USA) with 10% FBS (10%, Gibco, USA), streptomycin (0.1 mg/mL, Sigma, USA) and penicillin (100 units/mL, Sigma, USA). Regarding the oxygen and glucose deprivation (OGD) treatment, the cells were cultured in a glucose-free and FBS-free DMEM at the condition of the hypoxic incubator including 1% O_2_, 94% N_2_ and 5% CO_2_ at 37°C for 4 h. In the control group, the cells were incubated in a normoxic condition. To analyze the RNA stability, the cells were treated with actinomycin D (2 μg/ml, Solarbio, China). The control shRNA, lincRNA-EPS shRNA, HNRNPL shRNA, Htra1 shRNA, pcDNA3.1 vector, and pcDNA3.1 lincRNA-EPS vector were purchased from GenePharma, China, and transfected into the N-2a cells using Lipofectamine 2000 (Invitrogen, USA) according to the manufacturer’s instructions.

### Quantitative real time-PCR (qRT-PCR)

The qRT-PCR was performed as described previously [28]. Total RNAs from brain tissues or N-2a cells were isolated using the TRIzol reagent (Invitrogen, USA). Then, cDNA was synthesized using the TransScript One-Step gDNA Removal and cDNA Synthesis SuperMix (TransGen Biotech, China). Next, qRT-PCR was performed using an ABI Prism 7500 Sequence Detection System (Applied Biosystems, USA) with the primers and the TransStart TipTop Green qPCR SuperMix (TransGen Biotech, China). The primer sequences were as follows: lincRNA-EPS F: 5′-CAGATGAGAGAAGTGCGCGG-3′, R: 5′-TGGCCTGTTGTACCATGTGAT-3′; HNRNPL F: 5′-GCTAGGGTGGAAAGTGGGAC-3′, R: 5′-GGTCATCGTAGTTCTCCAGC-3′; Htra1 F: 5′-AAGGGCAGGAAGATCCCAAC-3′, R: 5′-TCGAGAAAGGAAGCTTGCGATA-3′; IL-6 F: 5′-ACAAAGCCAGAGTCCTTCAGAG-3′, R: 5′-TCTGTGACTCCAGCTTATCTCTTG-3′; TNF-α F: 5′-CCCTCACACTCACAAACCAC-3′, R: 5′- ACAAGGTACAACCCATCGGC-3′; IL-1β F: 5′-AGCTTCCTTGTGCAAGTGTCTG-3′, R: 5′-GACCACTCTCCAGTACCCACT-3′; GAPDH F: 5′- GGGTCCCAGCTTAGGTTCAT-3′, R: 5′- CCCAATACGGCCAAATCCGT-3′.

### Western blot analysis

Western blotting was performed as described previously [29]. Proteins were extracted from brain tissues or N-2a cells using protein lysis solution (Cell Signaling, USA) containing protease inhibitors (Sigma, USA) and quantitatively detected using the bicinchoninic acid (BCA) method (Thermo Fisher, USA). Next, the proteins (50 μg) were separated using 10% SDS-PAGE and transferred onto a PVDF membrane (Millipore, USA), followed by incubation with 5% milk and the primary antibodies (HNRNPL, no. #65043, 1:500, Cell Signaling, USA; Htra1, no. 55011-1-AP, 1:1000, Proteintech, USA; Bax, no. 50559-2-Ig, 1:1000, Proteintech, USA; Bcl-2, no. 12789-1-AP, 1:1000, Proteintech, USA; Caspase-3, no. #9662, 1:1000, Cell Signaling, USA; cleaved caspase-3, no. #9661, 1:500, Cell Signaling, USA; GAPDH, no. 60004-1-Ig, 1:2000, Proteintech, USA) at 4°C overnight. Afterward, the membranes were incubated with IgG secondary antibody (Proteintech, USA) at room temperature for 1 h. At last, an enhanced chemiluminescence reagent (Bio-Rad, USA) was used to visualize the membranes, and the Quantity 1 software (Bio-Rad, USA) was used to analyze the intensity of bands.

### Lactate dehydrogenase (LDH) assay

The death of the N-2a cells was assessed using a cytotoxicity LDH assay kit (Jiancheng Biotechnology, China) according to the manufacturer’s protocol as previously described [30].

### Terminal deoxynucleotidyl transferase dUTP nick end labeling (TUNEL)

The apoptosis rate was assessed using a TUNEL Apoptosis Assay kit (Roche, Germany) according to the product’s guidance in mice [31]. Briefly, the brain tissue sections were stained with the TUNEL dye for 1 h in a 37°C humidified atmosphere. Then, the sections were treated with diaminobenzidine, and counterstained with hematoxylin (to stain the cell nuclei). Finally, an optical microscope was used to visualize the apoptosis.

### 3-(4,5-dimethylthiazol-2-yl)-2,5-diphenyl tetrazolium bromide (MTT) assay

The viability of the N-2a cells was detected with MTT assay [32]. In brief, N-2a cells at a density of 3 × 10^3^ cells/well were plated into 96-well plates and MTT solution (20 μL, Beyotime, Jiangsu, China) was added to each well. After incubation for 2 h, the optical density of each well was detected at 590 nm using a microplate reader (BioTek Instruments, USA).

### RNA immunoprecipitation (RIP)

The interaction of lincRNA-EPS with HNRNPL and Htra1 was analyzed in the N-2a cells using a RIP Kit (Millipore, USA) [33]. Briefly, the N-2a cells were suspended through the lysis buffer (200 μl, RNase inhibitor 0.5 μl, protease inhibitor 1 μl). Then, magnetic beads were used to pretreat the anti-IgG, anti-HNRNPL, or anti-Htra1 antibodies, followed by immunoprecipitation of the samples at 4°C overnight. Finally, the The RNAs were purified from the protein-RNA complex and analyzed using qPCR with lincRNA-EPS primers.

### RNA pull-down

Using an RNA pull-down kit (Thermo, USA), we analyzed the direct interaction of lincRNA-EPS with HNRNPL and Htra1 [34]. Briefly, the Biotin-marked RNAs were transcribed *in vitro* and purified according to the manufacturer’s instructions, followed by the incubation of complete cell lysates. Biotin-labeled transcripts and interacted proteins were isolated with streptavidin beads and then subjected to western blot analysis.

### Analysis of cell apoptosis

The apoptosis rate of N-2a cells was determined using the Annexin V/PI staining kit (DOJINDO, Japan) based on the manufacturer’s protocols [28]. Shortly, we incubated the resuspended N-2a cells with 5 μL FITC-labeled Annexin-V for 10 min, and with 10 μL PI for 10 min in the dark at room temperature. At last, apoptotic cells were analyzed using a flow cytometer (BD Biosciences, USA).

### ROS production analysis

The ROS production was assessed by flow cytometry with 7’-Dichlorodihydrofluorescein Diacetate (DCFH-DA) [35]. After digestion and centrifugation, N-2a cells were collected to detect the production of ROS. N-2a cells were stained with 5 μmol/L DCFH-DA (Sigma, USA) for 20 min at room temperature. After resuspending in PBS, the production of ROS was determined using a flow cytometer (BD Biosciences, USA).

### Malondialdehyde (MDA) analysis

The levels of malondialdehyde (MDA) was analyzed by using the thiobarbituric acid reaction in rats [35]. Shortly, the rat brains were eliminated after reperfusion and smoothly homogenized. The tissue homogenates were incubated with thiobarbituric acid (0.67%, TBA) and trichloroacetic acid (10%, TCA), and were heated at 100°C for 30 minutes. Then, the supernatants were shifted into the 96-well plates and analyzed by applying a microplate reader (BioTek Instruments, USA) at 532 nm. Finally, the concentration of MDA was calculated using a standard curve, and the values were indicated as nmol/mg.

### The analysis of anti-oxidant enzyme activity

The brain was entirely eliminated and homogenized in a saline solution on ice. Then, the activity of superoxide dismutase (SOD) was measured using the SOD assay kit (Cayman Company, USA) [35].

### Statistical analysis

All statistical analyses were performed using the GraphPad prism 8.0 software. The results are presented as the means ± standard deviation. The statistical significance of the differences between the various groups was assessed using the one-way analysis of variance (ANOVA). The data of two groups were analyzed by using the Student’s *t*-test. Differences with a P value < 0.05 were deemed statistically significant.

## Results

### LincRNA-EPS alleviates OGD-induced N-2a cell injury

**Figure 1.**
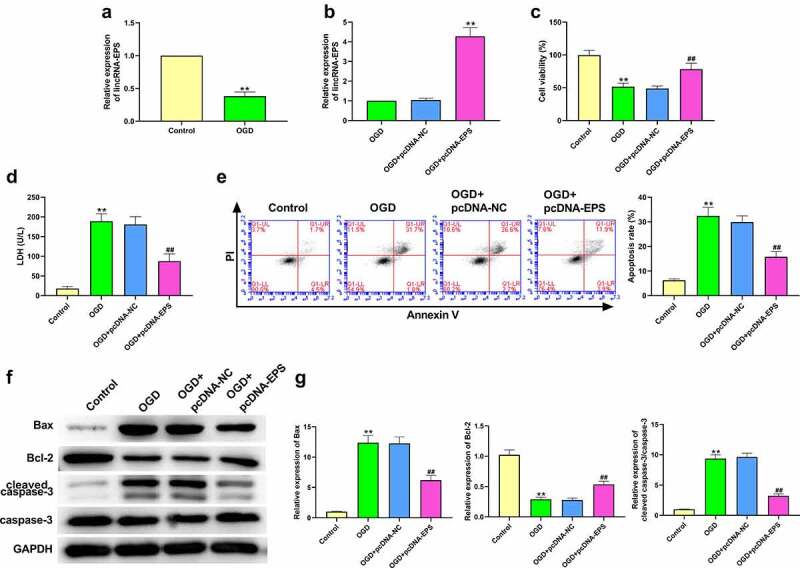
**LincRNA-EPS alleviates OGD-induced N-2a cell injury**. (a) After treatment with OGD, lincRNA-EPS level in N-2a cells was determined using qRT-PCR. (b-g) pcDNA3.1 or pcDNA3.1 lincRNA-EPS overexpression vectors was transfected into the OGD-treated N-2a cells. (b) The expression of lincRNA-EPS in N-2a cells was tested by qRT-PCR. (c) MTT assay was used to determine the viability of N-2a cells. (d) LDH level of N-2a cells was evaluated using a cytotoxicity LDH assay kit. (e) Flow cytometry was used to detect the apoptosis rate of N-2a cells. (f and g) Apoptosis related proteins expressions in N-2a cells were analyzed with western blot analysis. ** *P* < 0.01, ## *P* < 0.01.

We firstly investigated the effects of lincRNA-EPS on OGD-induced N-2a cell injury. As shown in Figure 1(a), the expression level of lincRNA-EPS was significantly lower in the OGD-treated N-2a cells, which implies that lincRNA-EPS may participate in the regulation of CIR injury. To validate this, we transfected the N2a cells with control vectors or lincRNA-EPS overexpression vectors, and verified the overexpression efficiency using qRT-PCR (Figure 1(b)). MTT assays showed that OGD treatment reduced the N-2a cells viability, in which lincRNA-EPS overexpression could rescue this phenotype (Figure 1(c)). Moreover, the results of (Figure 1(d)) showed that the LDH level was notably increased after OGD treatment, which could be reversed by lincRNA-EPS overexpression. Moreover, the overexpression of lincRNA-EPS attenuated the OGD-induced apoptosis of N-2a cells (Figure 1(e)). Similarly, the expression of Bax and cleaved caspase-3 was enhanced and the expression of Bcl-2 was inhibited in the OGD-treated N-2a cells, while these effects were significantly abolished by lincRNA-EPS overexpression (figure 1(f,g)). These results implied that lincRNA-EPS can alleviate OGD-induced N-2a cell injury.

### LincRNA-EPS inhibits OGD-induced inflammation and oxidative stress in N-2a cells

**Figure 2.**
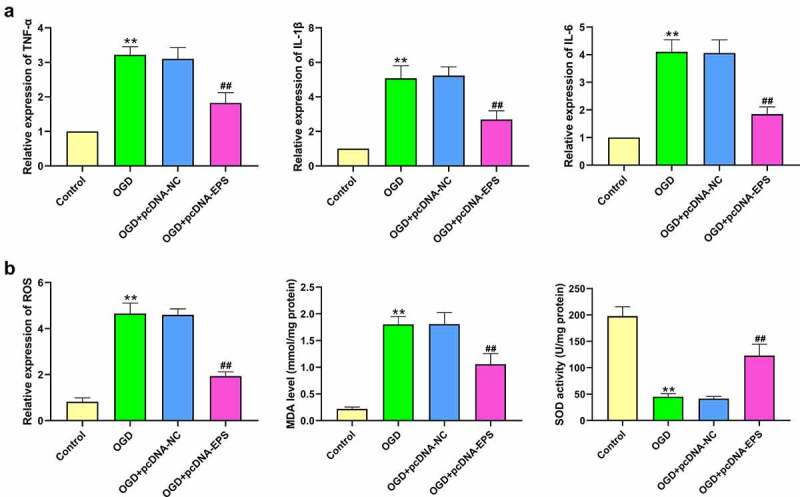
**LincRNA-EPS inhibits OGD-induced inflammation and oxidative stress in N-2a cells**. (a and b) The OGD-treated N-2a cells were treated with pcDNA3.1 or pcDNA3.1 lincRNA-EPS overexpression vectors. (a) The mRNA expression of TNF-α, IL-1β, and IL-6 was tested by qPCR in the cells. (b) The levels of ROS were analyzed by flow cytometry analysis based on DCFH-DA staining in the cells. The levels of MDA and SOD were assessed in the cells. ** *P* < 0.01, ## *P* < 0.01.

Subsequently, we further assessed the association of lincRNA-EPS with inflammation and oxidative stress in the OGD-treated N-2a cells. Remarkably, the expression levels of IL-6, TNF-α, and IL-1β were enhanced in the OGD-treated N-2a cells, which was abolished by the overexpression of lincRNA-EPS (Figure 2a). Meanwhile, the levels of ROS and MDA were enhanced, while SOD levels were reduced by OGD treatment, an effect which can we reversed through the lincRNA-EPS overexpression (Figure 2(b)). Taken together, these data suggest that lincRNA-EPS inhibits the OGD-induced inflammation and oxidative stress in N-2a cells.

### LincRNA-EPS enhances the stability of Htra1 by interacting with HNRNPL in N-2a cells

**Figure 3.**
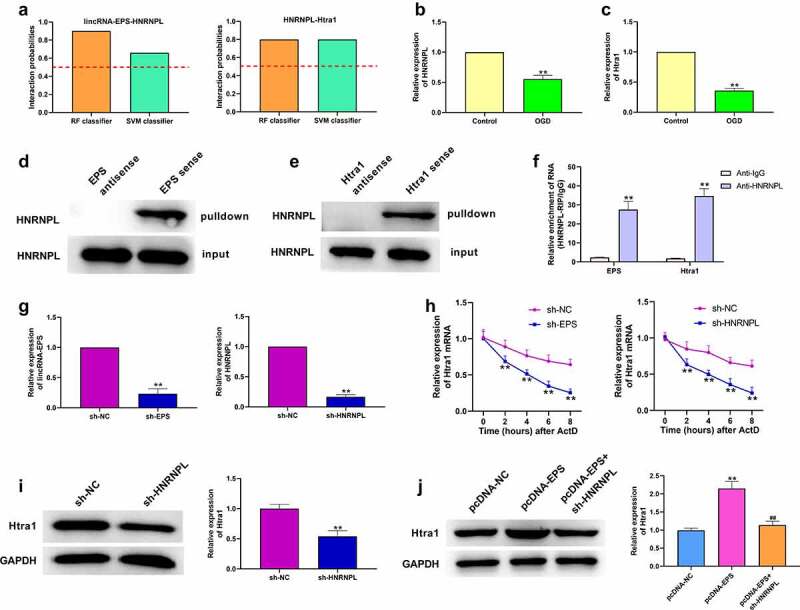
**LincRNA-EPS enhances stability of Htra1 by interacting with HNRNPL in N-2a cells**. (a) The interaction of lincRNA-EPS, Htra1, and HNRNPL was analyzed by bioinformatics analysis based on RPISeq database. (b) The mRNA expression of HNRNPL was measured by qPCR in the OGD-treated N-2a cells. (c) The mRNA expression of Htra1 was tested by qPCR in the OGD-treated N-2a cells. (d and e) The interaction of lincRNA-EPS with HNRNPL and Htra1 was determined by RNA pull down assays in the N-2a cells. (f) The interaction of lincRNA-EPS with HNRNPL and Htra1 was assessed by RIP assays in the N-2a cells. (g) The N-2a cells were treated with lincRNA-EPS shRNA or HNRNPL shRNA. The expression of lincRNA-EPS or HNRNPL was tested with qRT-PCR. (h) The N-2a cells were co-treated with ActD and control shRNA, lincRNA-EPS shRNA, or HNRNPL shRNA. The mRNA expression of Htra1 was examined by qPCR assays in the cells. (i) The N-2a cells were treated with control shRNA or HNRNPL shRNA. The expression of Htra1 was measured by Western blot analysis in the cells. (j) The N-2a cells were treated with pcDNA3.1 or pcDNA3.1 lincRNA-EPS overexpression vectors, or co-treated with pcDNA3.1 lincRNA-EPS overexpression vectors and HNRNPL shRNA. The expression of Htra1 was analyzed with western blot analysis in the cells. ** *P* < 0.01, ## *P* < 0.01.

Next, we tried to assess the mechanism underlying lincRNA-EPS-mediated CIR injury. Using bioinformatics analysis (http://pridb.gdcb.iastate.edu/RPISeq/), we identified the interaction probabilities of lincRNA-EPS or Htra1 with HNRNPL . Predictions with probabilities >0.5 were considered ‘positive’, which indicates that the corresponding RNA and protein are likely to interact. The results in Figure 3(a) revealed an interaction between lincRNA-EPS or Htra1 and HNRNPL. The expression of Htra1 and HNRNPL was decreased in the OGD-treated N-2a cells (Figure 3(b,c)). To confirm this hypothesis, we performed RNA pull down and RIP assays, and found that HNRNPL could bind with lincRNA-EPS or Htra1 (Figure 3(d-f)). Then, we validated the efficiency of lincRNA-EPS and HNRNPL knockdown in N-2a cells (Figure 3(g)). We speculated that lincRNA-EPS might have an impact on Htra1 expression through modulating the Htra1 mRNA stability. After treating with Actinomycin D (ActD) to block mRNA generation, the expression of Htra1 mRNA was assessed using RT-qPCR. The resulting data showed that the depletion of both lincRNA-EPS and HNRNPL remarkably decreased Htra1 mRNA expression in N-2a cells (Figure 3(h)). Meanwhile, the HNRNPL depletion significantly inhibited Htra1 expression (Figure 3(i)). Moreover, the overexpression of lincRNA-EPS significantly enhanced the expression of Htra1, and HNRNPL silencing could reverse this function (Figure 3(j)). This implied that lincRNA-EPS enhances the stability of Htra1 by interacting with HNRNPL in N-2a cells.

### LincRNA-EPS attenuates OGD-induced N-2a cell injury by maintaining Htra1 stability

**Figure 4.**
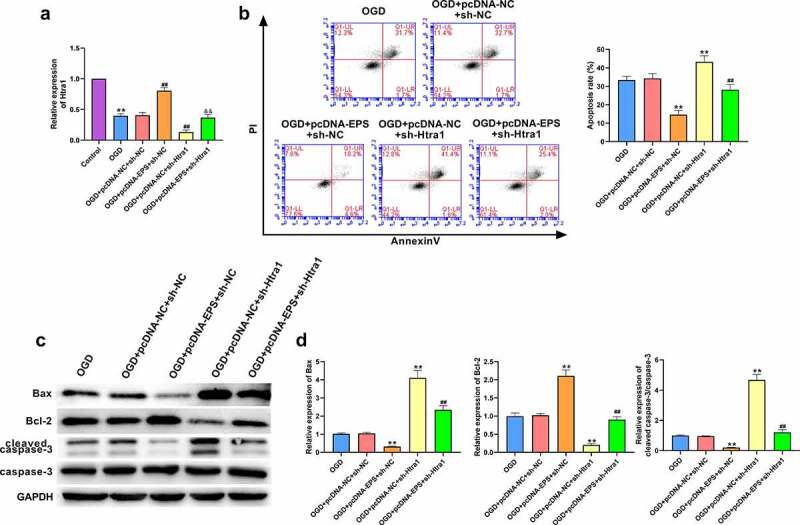
**LincRNA-EPS attenuates OGD-induced N-2a cell injury by increasing Htra1 expression**. (a-d) The OGD-treated N-2a cells were co-treated with pcDNA3.1 lincRNA-EPS overexpression vectors and Htra1 shRNA. (a) qRT-PCR was used to detected Htra1 expression in N-2a cells. (b) Flow cytometry was used to determine the apoptosis rate of N-2a cells. (c and d) Apoptosis related proteins expressions in N-2a cells were analyzed with western blot analysis. ** *P* < 0.01, ## *P* < 0.01, && *P* < 0.01.

Next, we further investigated whether lincRNA-EPS modulated the OGD-induced N-2a cell injury by maintaining Htra1. Our findings indicated that the expression of Htra1 was decreased in the OGD-treated N-2a cells (Figure 4(a)). The overexpression of lincRNA-EPS was able to enhance Htra1 expression in N-2a cells, an effect that could be reversed through the depletion of Htra1 (Figure 4(a)). Moreover, the depletion of Htra1 significantly limited the inhibition of N-2a cells cells through lincRNA-EPS overexpression (Figure 4(b)). In addition, the expression of Bax and cleaved caspase-3 was reduced, while the expression of Bcl-2 was increased by lincRNA-EPS overexpression in the OGD-treated N-2a cells, and Htra1 knockdown could reverse this effect (Figure 4(c,d)). Together these results indicate that lincRNA-EPS attenuates OGD-induced N-2a cell injury by maintaining Htra1 stability.

### LincRNA-EPS relieves OGD-induced inflammation and oxidative stress in N-2a cells by maintaining Htra1 stability

**Figure 5.**
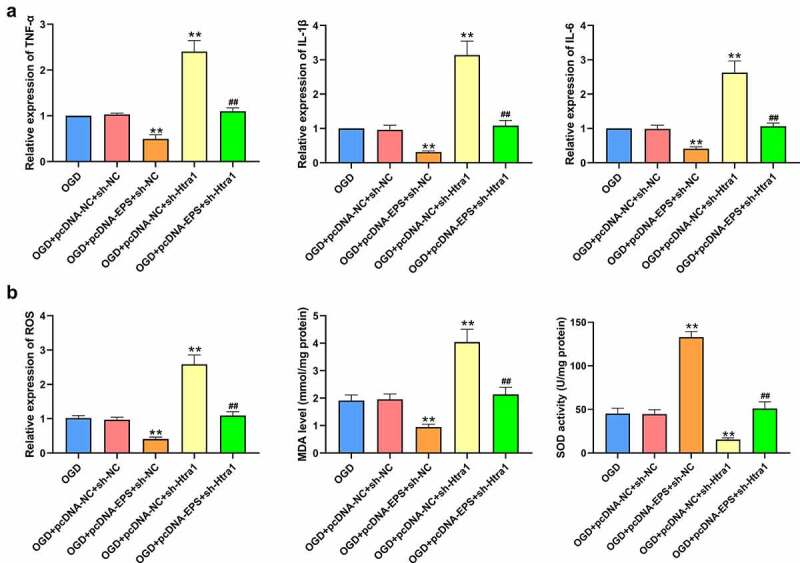
**LincRNA-EPS relieves OGD-induced inflammation and oxidative stress in N-2a cells by enhancing Htra1 expression**. (a and b) The OGD-treated N-2a cells were co-treated with pcDNA3.1 lincRNA-EPS overexpression vectors and Htra1 shRNA. (a) The mRNA expression of TNF-α, IL-1β, and IL-6 was tested by qPCR in the cells. (b) The levels of ROS were analyzed by flow cytometry analysis based on DCFH-DA staining in the cells. The levels of MDA and SOD were detected in the cells. ** *P* < 0.01, ## *P* < 0.01.

Then, we evaluated the role of the lincRNA-EPS/Htra1 axis in the modulation of inflammation and oxidative stress in OGD-treated N-2a cells. The results showed that the expression levels of IL-6, TNF-α, and IL-1β were significantly reduced by the overexpression of lincRNA-EPS, while the depletion of Htra1 could reverse these phenotypes (Figure 5(a)). Besides, the levels of ROS and MDA were inhibited while the SOD levels were enhanced by lincRNA-EPS overexpression in OGD-treated N-2a cells, and Htra1 knockdown could reverse this effect (Figure 5(b)). These findings suggest that lincRNA-EPS relieves OGD-induced inflammation and oxidative stress in N-2a cells by maintaining Htra1 stability.

### LincRNA-EPS alleviates CIR injury in vivo

**Figure 6.**
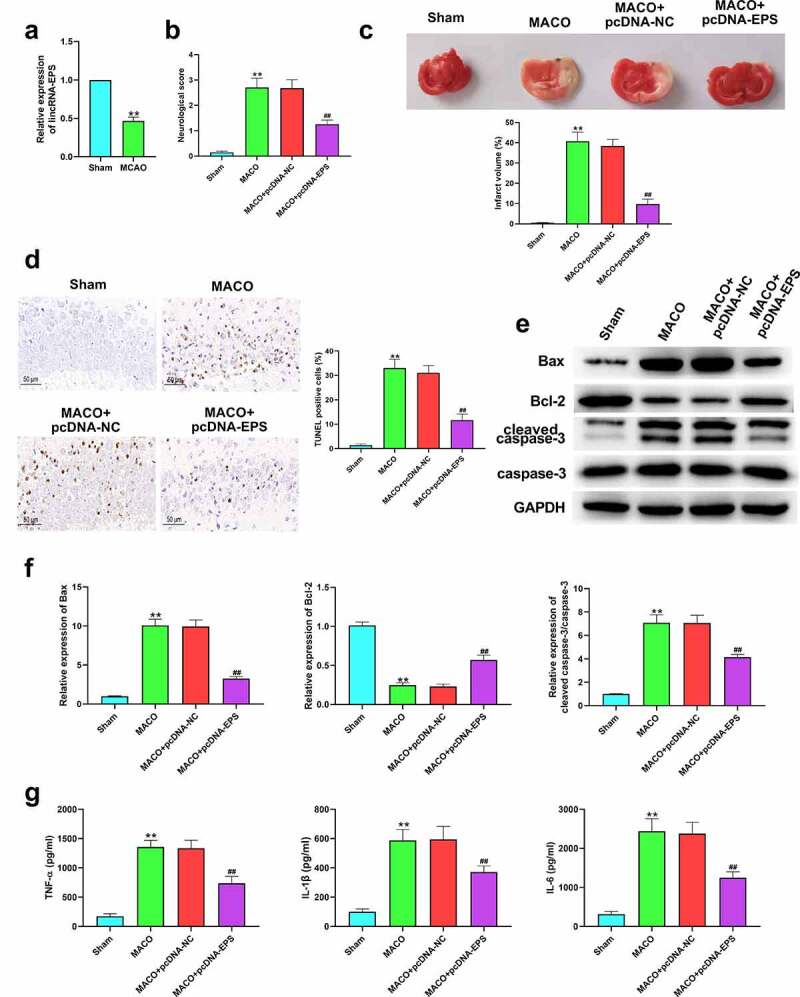
**LincRNA-EPS alleviates CIR injury *in vivo.*** (a-g) The control vector or pcDNA3.1-lincRNA-EPS was injected into the cortex of the MCAO/R-treated mice at three points by a stereotaxia instrument. (a) The expression of lincRNA-EPS in the brain tissues of the mice was measured by qRT-PCR. (b) The neurological deficit scores were measured after 24 hours of reperfusion. (c) TCC staining was used to determine cerebral infarction volume in the mice. (d) The cell apoptosis of brain tissues was determined with TUNEL staining. (e and f) Apoptosis related proteins expressions in the brain tissues of mice were analyzed with western blot analysis.(g) The level of TNF-α, IL-1β, and IL-6 in the serum of the mice was analyzed by ELISA. ** *P* < 0.01, ## *P* < 0.01.

We also assessed the effects of lincRNA-EPS on CIR injury *in vivo*. Our findings showed that the expression of lincRNA-EPS was significantly down-regulated in the MCAO/R mice relative to the sham mice (Figure 6(a)). The neurological score was elevated through the MCAO/R treatment, in which lincRNA-EPS overexpression markedly reversed this effect (Figure 6(b)). Meanwhile, TTC staining showed the overexpression of lincRNA-EPS to decrease the infarct volume of brain in MCAO/R mice (Figure 6(c)). TUNEL analysis revealed that lincRNA-EPS overexpression attenuated MCAO/R-induced apoptosis in mice (Figure 6(d)). Similarly, there was an enhancement in the expression of Bax and cleaved caspase-3 and an inhibition in the expression of Bcl-2 in MCAO/R mice, while these effects were significantly abolished by lincRNA-EPS overexpression (Figure 6(e,f)). Moreover, the expression of IL-6, TNF-α, and IL-1β was up-regulated in the MCAO/R mice, while the overexpression of lincRNA-EPS was able to inhibit their levels (Figure 6(g)). All data indicate that lincRNA-EPS alleviates CIR injury *in vivo*.

## Discussion

Stroke is accompanied by pathological inflammation, oxidative stress, and apoptosis [6]. Recently, lncRNAs are reported to play important roles in CIR injury [32]. In this study, we found that lincRNA-EPS inhibits CIR injury by increasing Htra1stability through recruiting HNRNPL.

Several previous studies have identified that multiple lncRNAs play critical functions in the modulation of the myocardial infarction progression. LncRNA MEG3 is reported to increase CIR injury by regulating the miR-485/AIM2 axis [32]. LncRNA SNHG12 inhibits CIR injury by activating the AMPK signaling through the miR-199a/SIRT1 axis [36]. LncRNA MALAT1 promotes CIR injury by targeting miR-145/AQP4 [27], and lncRNA H19 contributes to CIR injury by activating autophagy [37]. LincRNA-EPS enhances inflammation and neurogenesis in cerebral infarction [38]. In this study, we showd that the expression of lincRNA-EPS was down-regulated in the OGD-treated N-2a cells and MCAO/R mice. LincRNA-EPS was able to inhibit the OGD-induced cell injury, inflammation, and oxidative stress in N-2a cells. Meanwhile, lincRNA-EPS could alleviate CIR injury *in vivo*. These results demonstrate that lincRNA-EPS has a crucial role of in attenuating CIR injury.

HNRNPL has been found to participate in regulating brain disorders. It has been shown to form a complex with lncRNA SChLAP1 to regulate glioblastoma by activating NF-κB signaling and stabilizing ACTN4 [39]. HNRNPL modulates brain injury by inhibiting the mitochondrial metabolism by post- of isocitrate dehydrogenase (IDH) [40]. Moreover, Htra1 has been reported to modulate cerebrovascular disorder, such that its mutation is correlated with cerebral small vessel disease [41], and it is abnormally expressed in astrocytes to regulate astrocyte injury and development [42]. Furthermore, Htra1 promotes the formation of the apolipoprotein E to stimulate neurogenesis [43]. In our mechanical investigation, lincRNA-EPS was able to interact with both Htra1 and HNRNPL, and the depletion of lincRNA-EPS and HNRNPL reduced the mRNA stability in N-2a cells. HNRNPL knockdown reversed the effect of lincRNA-EPS overexpression on enhancing the Htra1 levels. LincRNA-EPS inhibited OGD-induced inflammation and oxidative stress by the HNRNPL/Htra1 axis in N-2a cells. This indicates a novel correlation of lincRNA-EPS with Htra1 and HNRNPL, which implies a new mechanism that involves lincRNA-EPS, HNRNPL, and Htra1 in the modulation of CIR injury. It should be mentioned that our study has some limitations, and further investigation is still needed to determine whether lincRNA-EPS can influence CIR injury through interfering with Htra1 *in vivo*.

## Conclusions

We showed that lincRNA-EPS attenuated CIR injury by maintaining Htra1 stability through recruiting HNRNPL. Our findings present novel insights in the possibility of considering LincRNA-EPS, Htra1 and HNRNPL as potential therapeutic targets for CIR injury.

## Supplementary Material

Supplemental Material

## Data Availability

The datasets used and analyzed during the current study are available from the corresponding author.

## Abbreviations

cerebral ischemia/reperfusion (CIR), oxygen and glucose deprivation (OGD), Neuro-2A (N-2a), middle cerebral artery occlusion/repression (MCAO/R), interleukin-6 (IL-6), tumor necrosis factor-alpha (TNF-α), interleukin-1β (IL-1β), reactive oxygen species (ROS), malondialdehyde (MDA), superoxide dismutase (SOD), RNA-binding proteins (RBPs), Heterogeneous nuclear ribonucleoproteins (HNRNPs), high-temperature requirement protein A1 (Htra1)
