# Supplementary material for: Long non-coding RNA lincRNA-erythroid prosurvival (EPS) alleviates cerebral ischemia/reperfusion injury by maintaining high-temperature requirement protein A1 (Htra1) stability through recruiting heterogeneous nuclear ribonucleoprotein L (HNRNPL)
Source: Bioengineered. 2022 May 13;13(5):12248–60. doi: 10.1080/21655979.2022.2074738 (PMC9275866; doi:10.1080/21655979.2022.2074738)
Supplement: Supplemental Material [file KBIE_A_2074738_SM0221.zip › supplementary/certificate.pdf]

EDITSPRINGS

EDITORIAL CERTIFICATE

This is to certify that the manuscript detailed below was edited by one or more of our highly qualified, native English speakers at EditSprings, to assure compliance with Anglophone academic standards in terms of style, punctuation, grammar, and spelling.

Manuscript title:

Long non-coding RNA lincRNA-erythroid prosurvival (EPS) alleviates cerebral ischemia/reperfusion injury by maintaining high-temperature requirement protein A1 (Htra1) stability through recruiting heterogeneous nuclear ribonucleoprotein L (HNRNPL)

Authors:

Haifeng Guo, Xia Guo, Shiting Jiang

Date Issued:

Apr 21 2022

Certificate Number:

ES-202204151156288730

This certificate can be verified on https://www.editsprings.cn/QueryCertificate.html EditSprings hereby certifies that neither content nor the author's intentions were altered in any way during the editing process. Documents in receipt of this certification should be ready for publication as far as style and language are concerned, provided that the author(s) accepted our suggestions and changes (which remains their right and responsibility).

EditSprings offers a wide range of editing, translation, for researchers and publishers across the world. Our highly skilled editors are all established academics based in Anglophone Higher Education institutions across the world (U.K., U.S.A., Canada, Australia, and elsewhere), are experts in their respective fields, and are qualified to edit research papers authored by non-Anglophone scholars.
