# Supplementary material for: Long non-coding RNA lincRNA-erythroid prosurvival (EPS) alleviates cerebral ischemia/reperfusion injury by maintaining high-temperature requirement protein A1 (Htra1) stability through recruiting heterogeneous nuclear ribonucleoprotein L (HNRNPL)
Source: Bioengineered. 2022 May 13;13(5):12248–60. doi: 10.1080/21655979.2022.2074738 (PMC9275866; doi:10.1080/21655979.2022.2074738)
Supplement: Supplemental Material [file KBIE_A_2074738_SM0221.zip › supplementary/ethical.pdf]

济南市中医医院
动物伦理审查文件

伦理号：（DW20200112）

项目名称	Long non-coding RNA lincRNA-EPS alleviates cerebral ischemia/reperfusion injury by maintaining Htra1 stability through recruiting HNRNPL
申请科室	脑病科
项目负责人	郭海峰
主要人员	郭海峰，郭霞，蒋士廷
项目类别	□新药物临床试验<br>□新器械临床试验<br>□新技术应用<br>□人体标本收集<br>√科研论文<br>其他（请注明）：________________
项目简介（包括理论依据、项目内容、具体实施细则）	采用 6 周龄 C57BL/6J 小鼠(雄性，20-25g)建立 MCAO/R 模型，分析 CIR 损伤情况。小鼠(n = 6)被戊巴比妥钠(30 mg/kg)麻醉，固定在手术加热台上，并在呼吸机中孵育以维持生命。然后切开颈部，显露右侧颈总动脉，从颈总动脉经颈外动脉将 4-0 尼龙丝从颈总动脉经颈外动脉插入颈内动脉末端，阻断血液。阻断 2 小时后，恢复补血 24 小时。假手术组小鼠进行类似的手术，无 MCAO/R 闭塞。MCAO 前 5 天，在脑室内注射对照载体或 pcDNA3.1-lincRNA-EPS。再灌注 24 h 后测量神经功能评分，处死小鼠，取脑组织进行后续分析。
申请人（项目负责人）承诺	以上所填内容均属实，如获批，我将严格按照提供的方案进行研究并遵守医学伦理委员会的相关规定。<br><br>签字：郭海峰 2020 年 1 月 12 日
科室意见	同意<br><br>签字： 2020 年 1 月 12 日
伦理委员会审核意见	同意<br><br>（盖章）：济南市中医医院 2020 年 1 月 12 日
