# Supplementary material for: Long non-coding RNA lincRNA-erythroid prosurvival (EPS) alleviates cerebral ischemia/reperfusion injury by maintaining high-temperature requirement protein A1 (Htra1) stability through recruiting heterogeneous nuclear ribonucleoprotein L (HNRNPL)
Source: Bioengineered. 2022 May 13;13(5):12248–60. doi: 10.1080/21655979.2022.2074738 (PMC9275866; doi:10.1080/21655979.2022.2074738)

Western blot images

Fig 1F


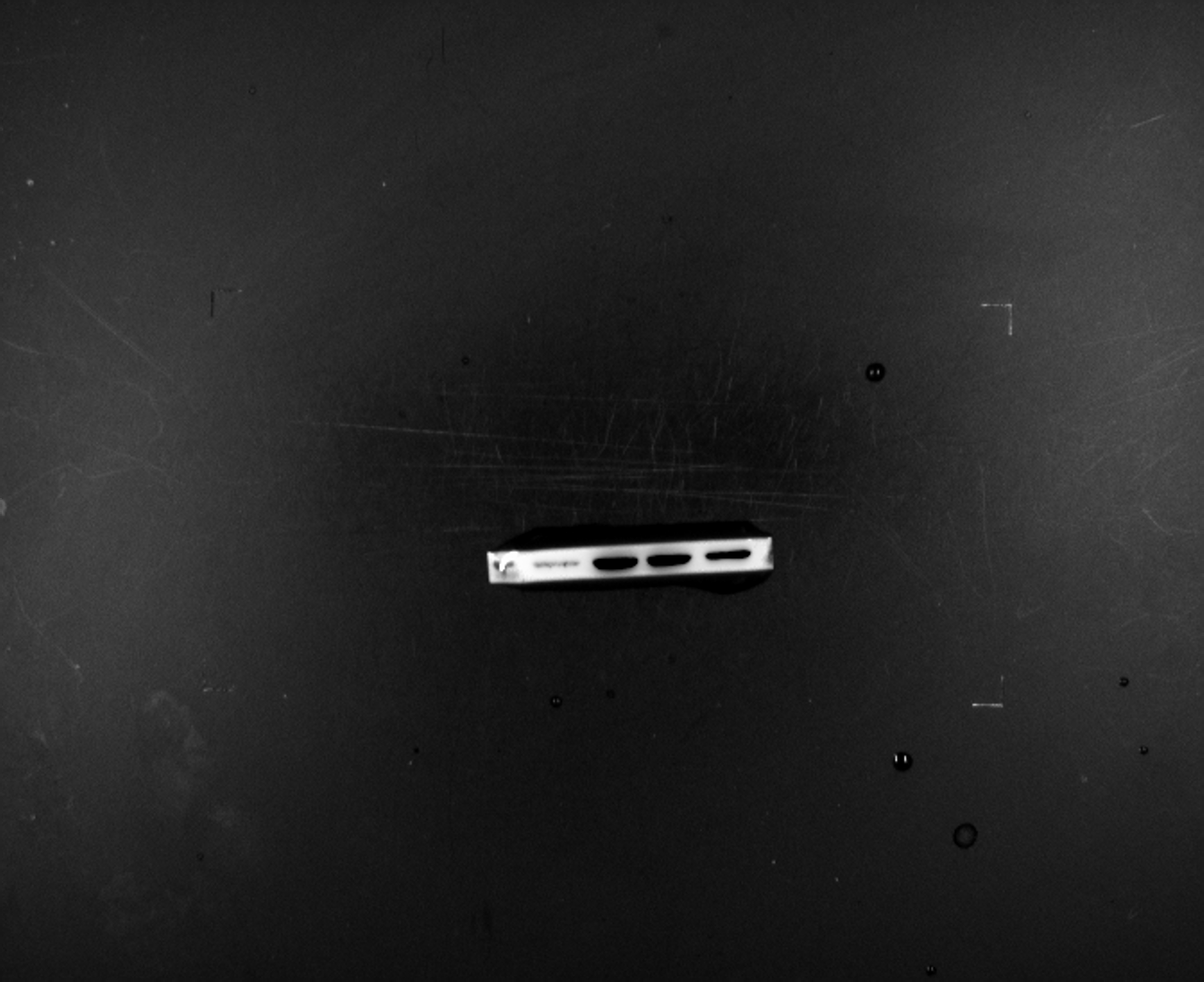

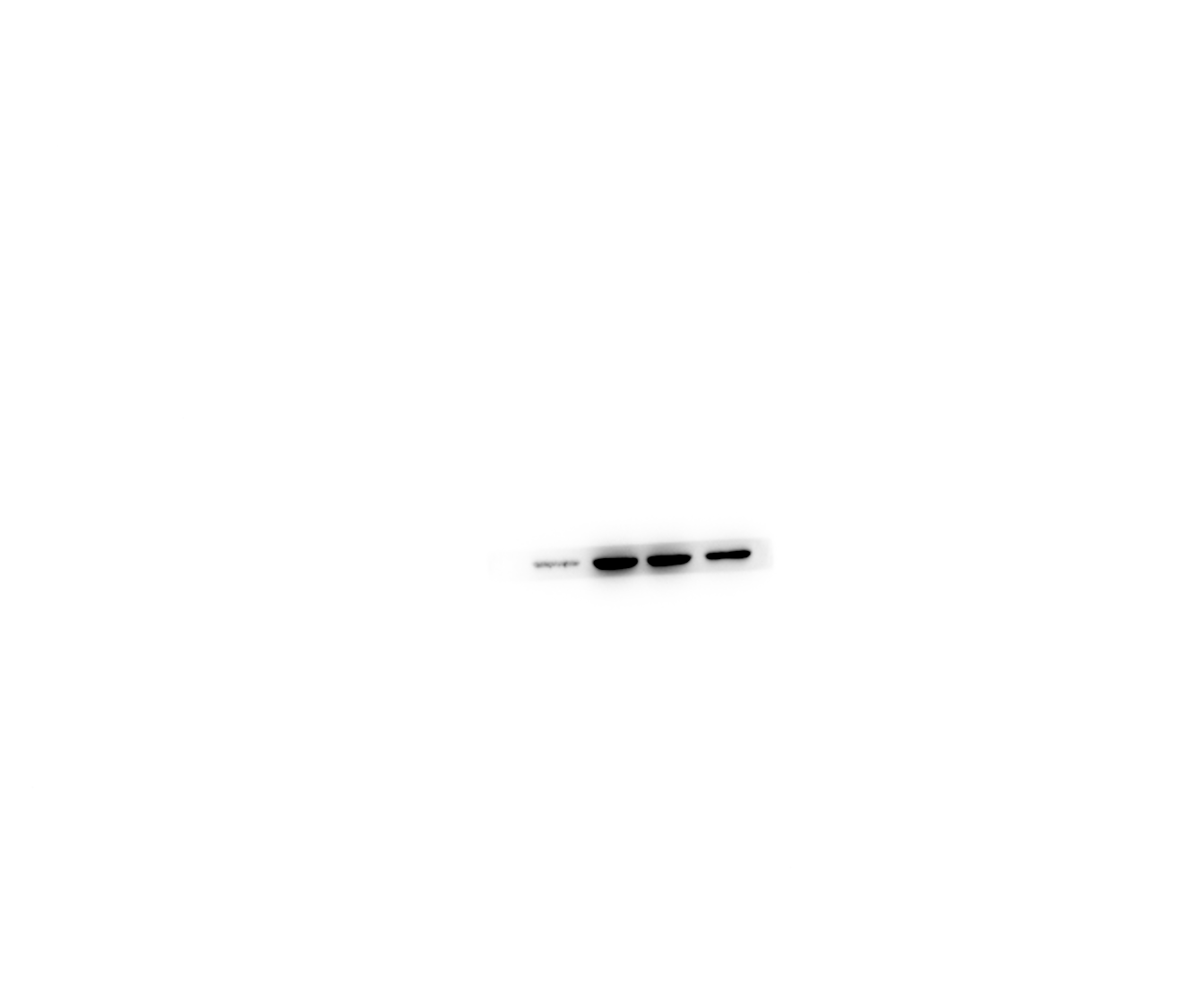

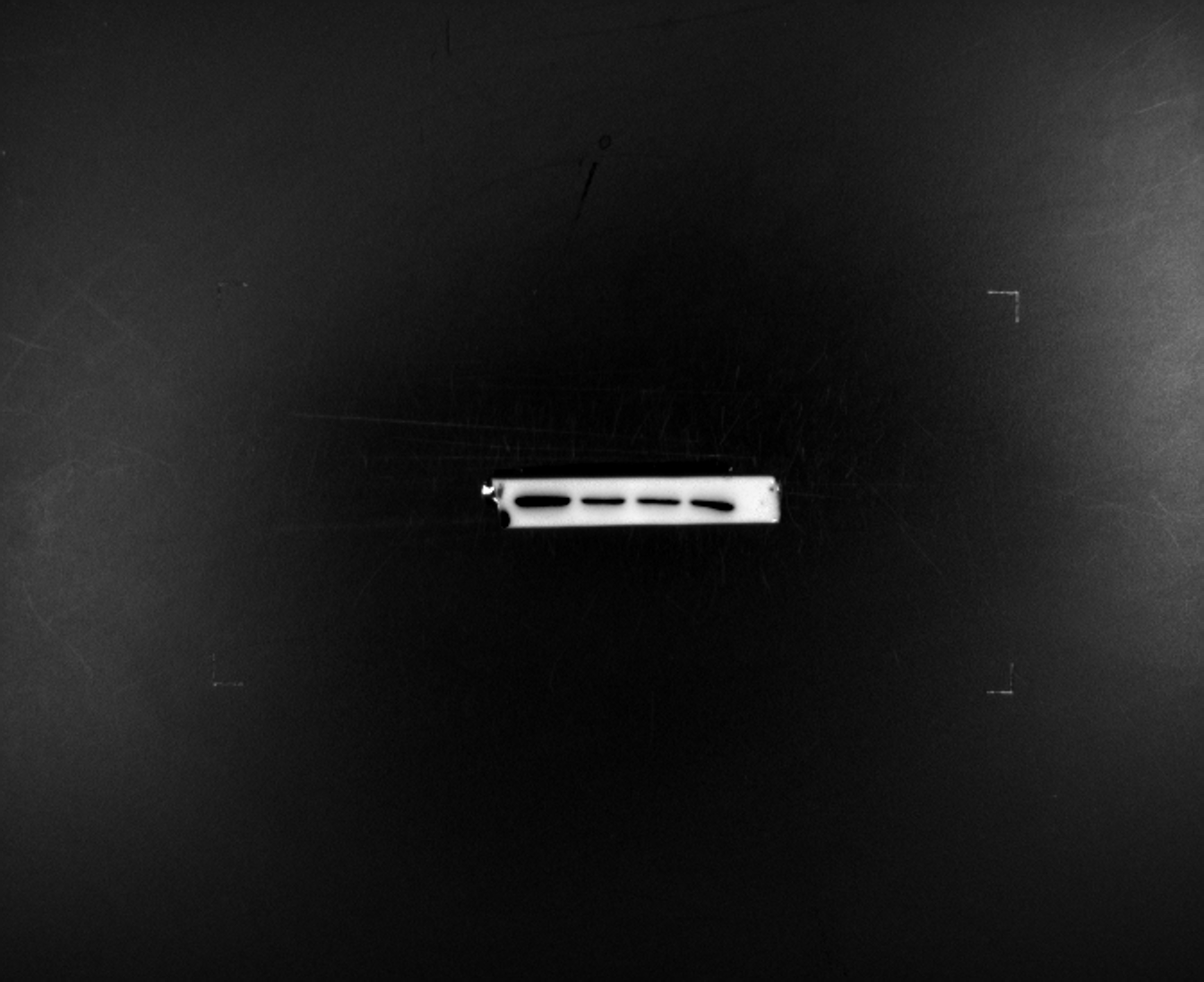

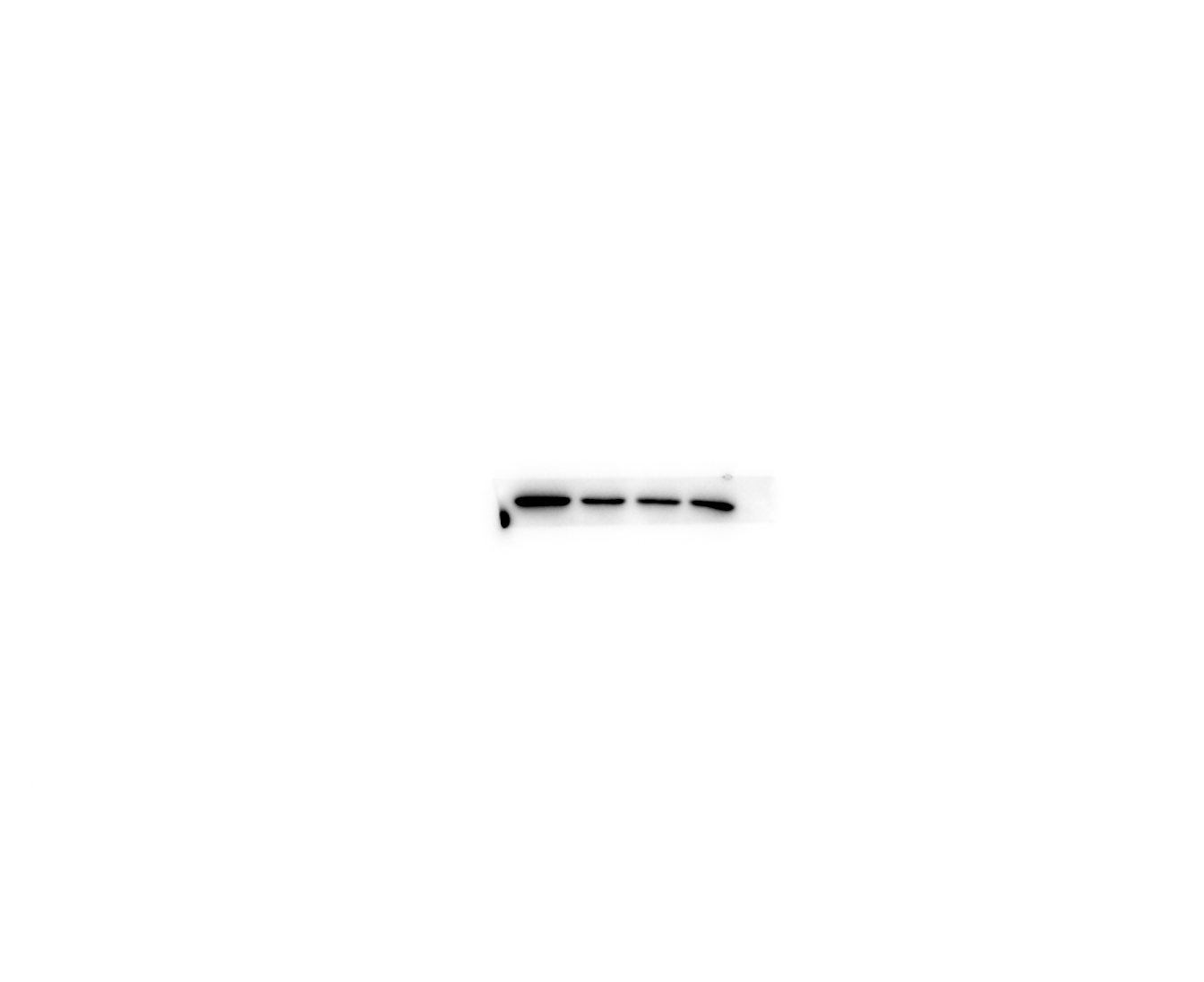


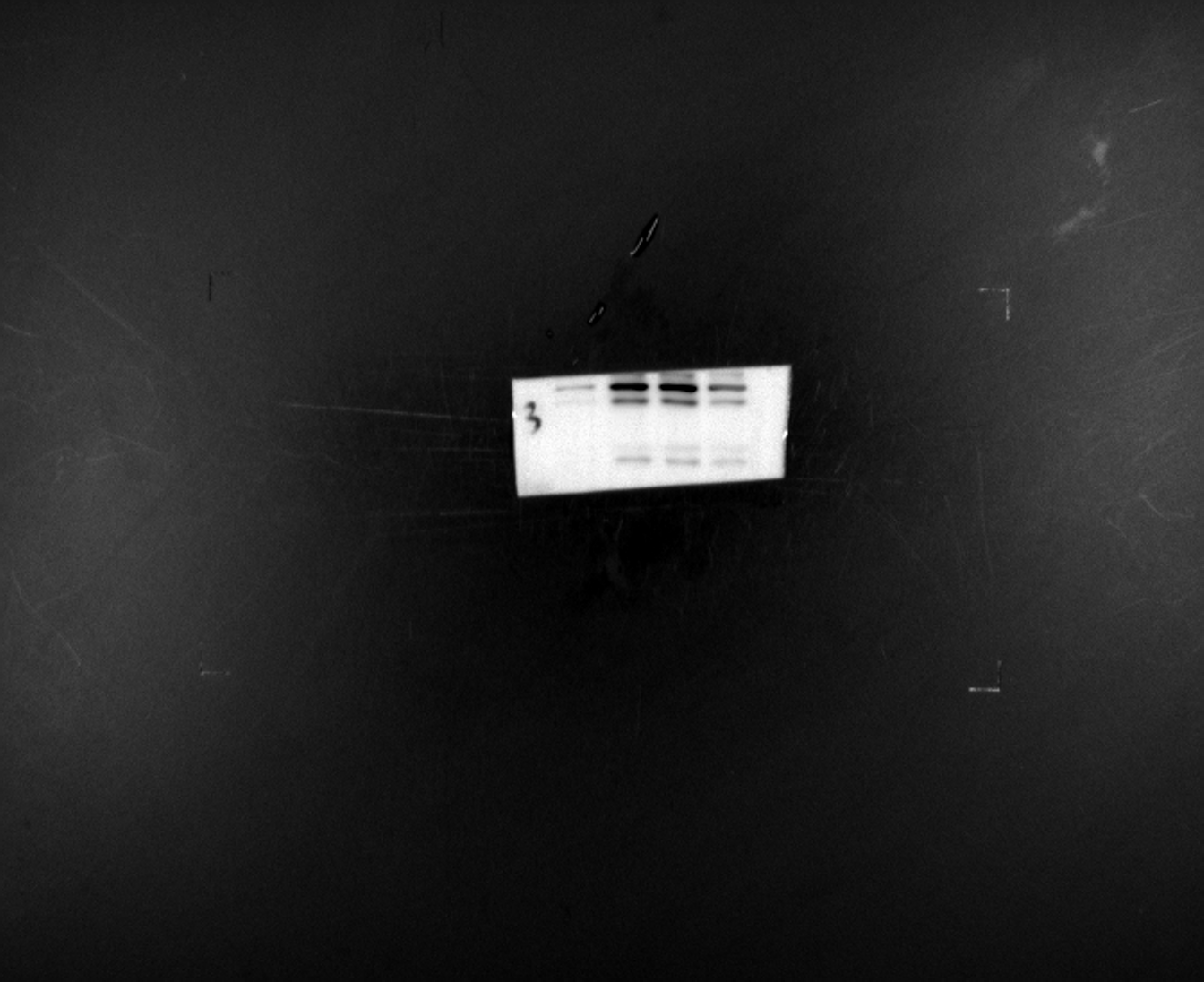


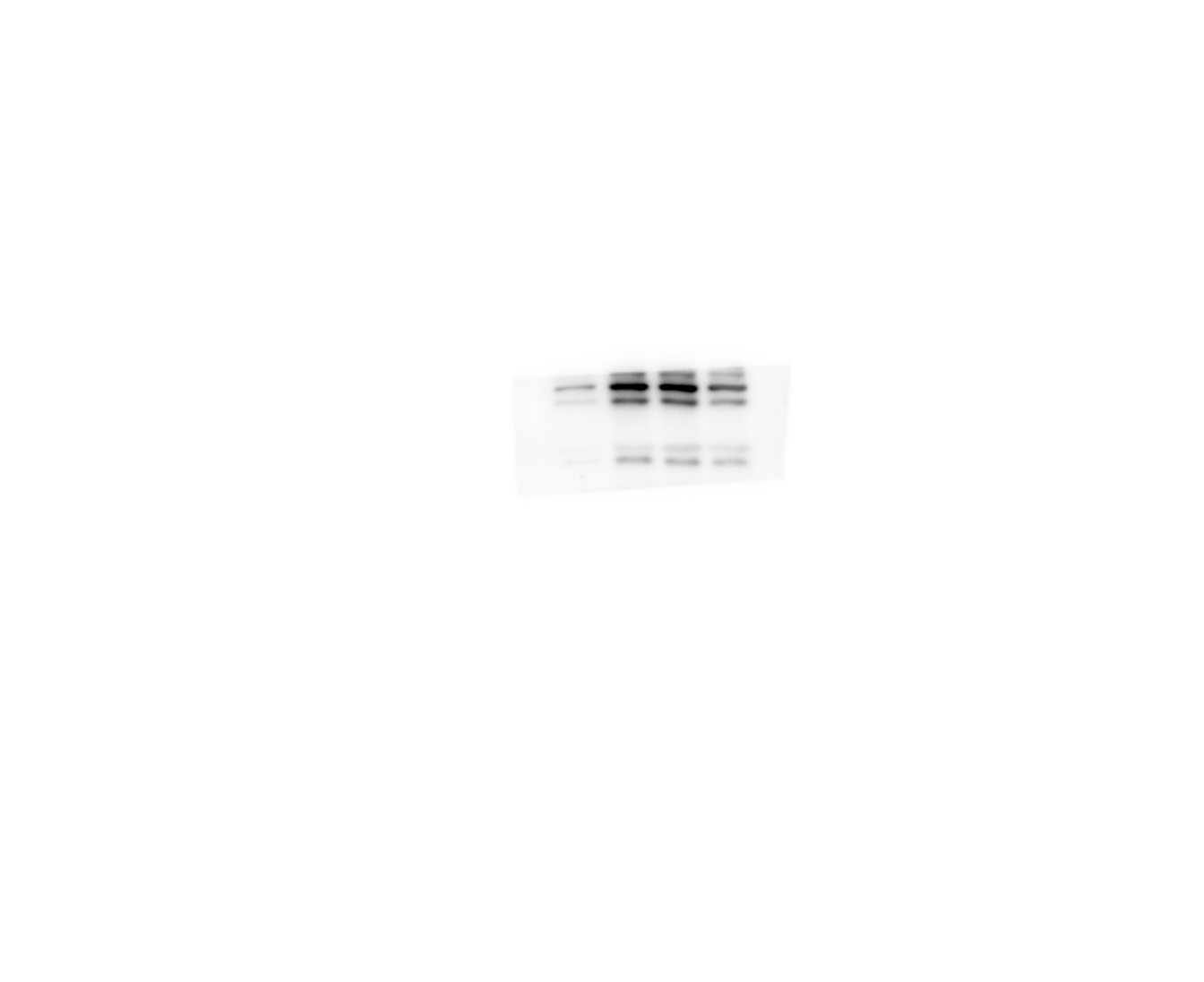

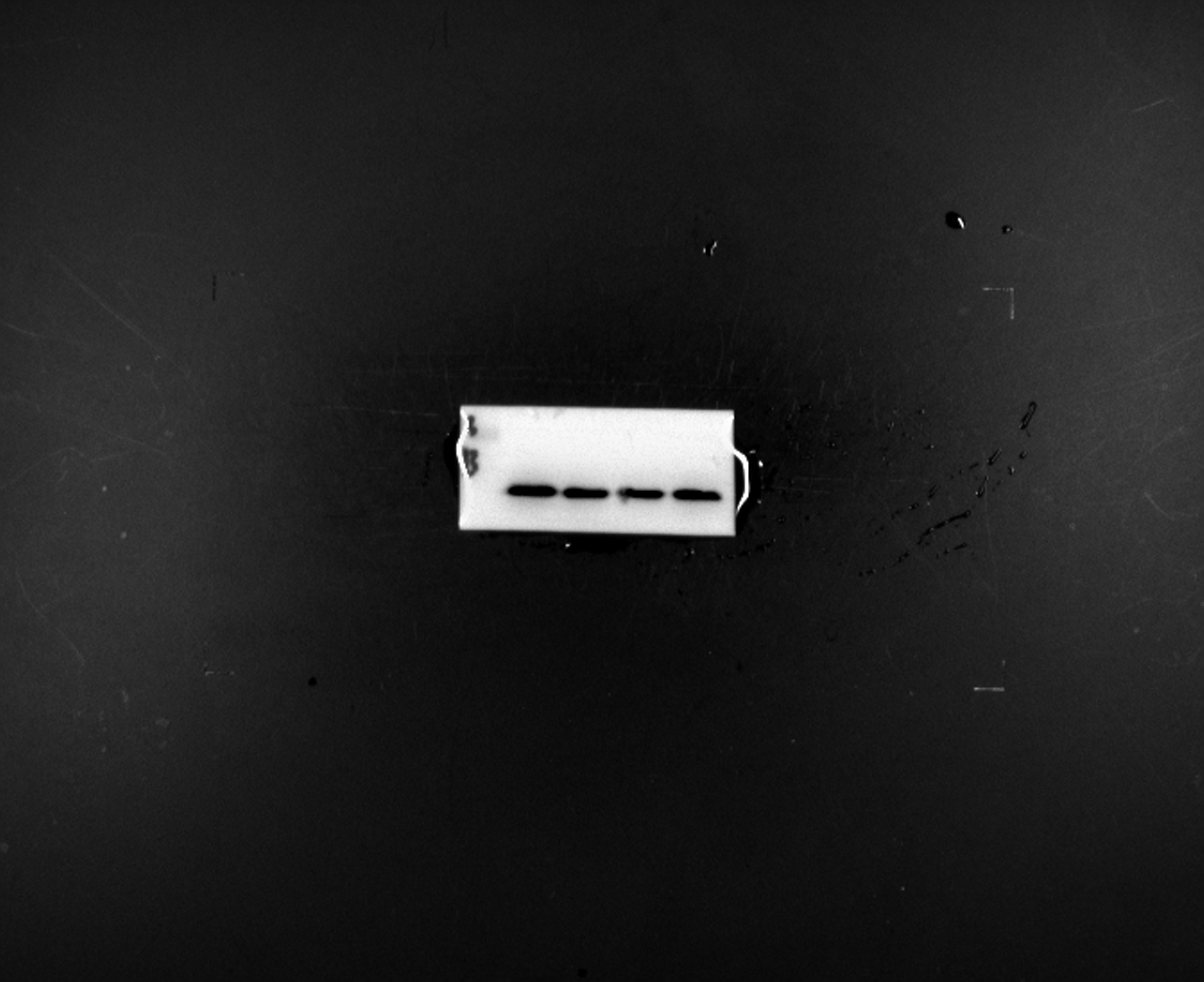

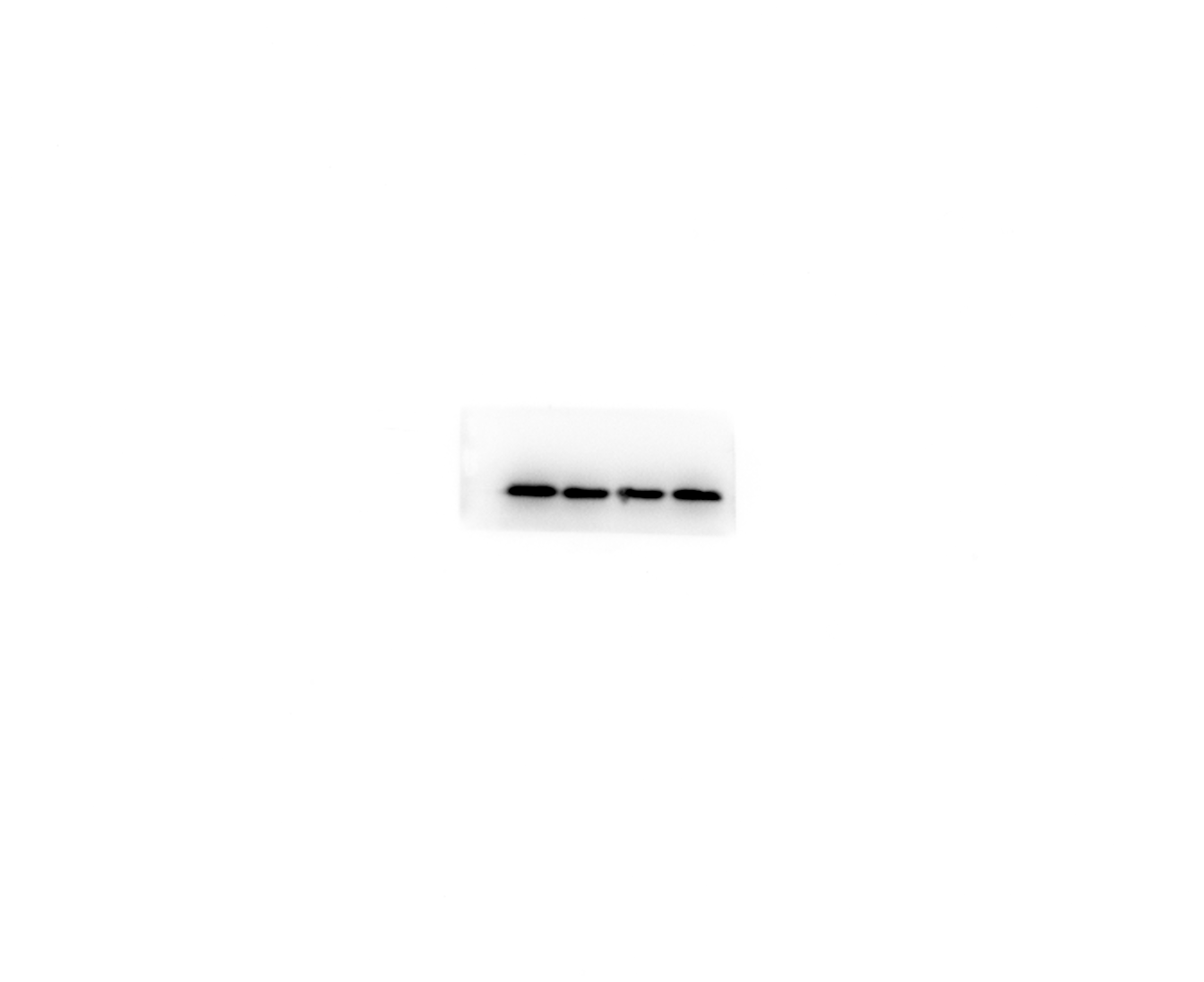

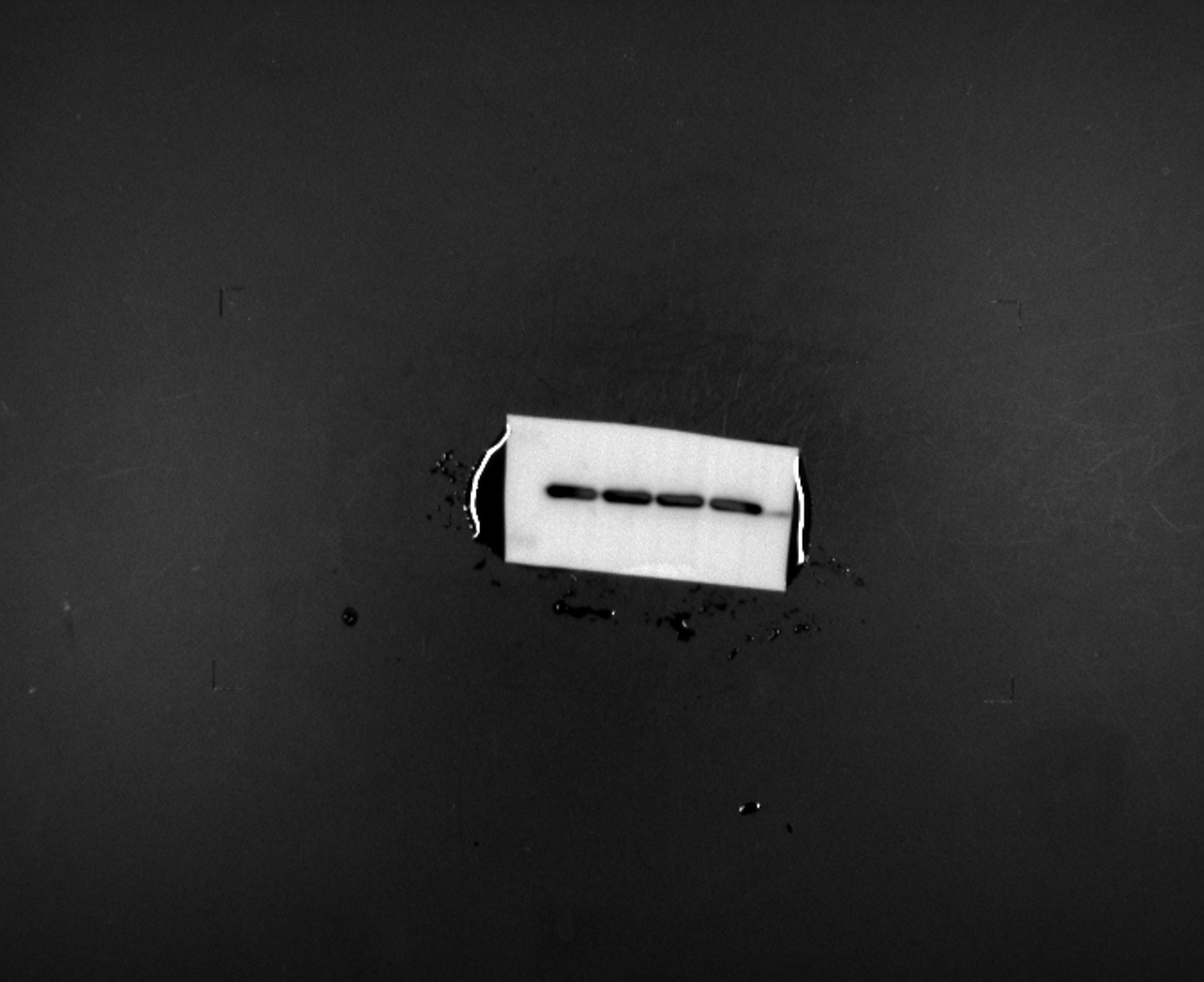

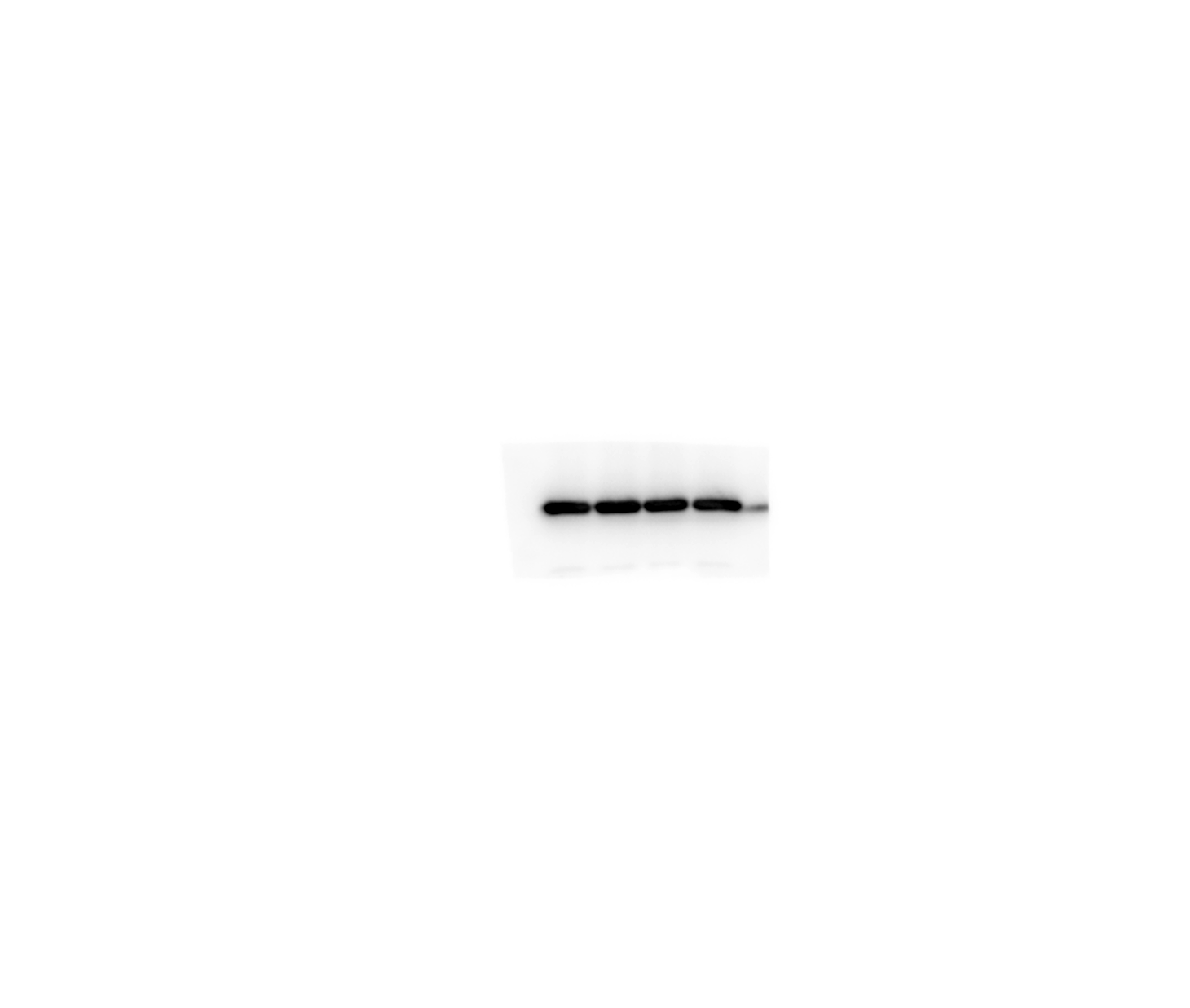


Fig 3D


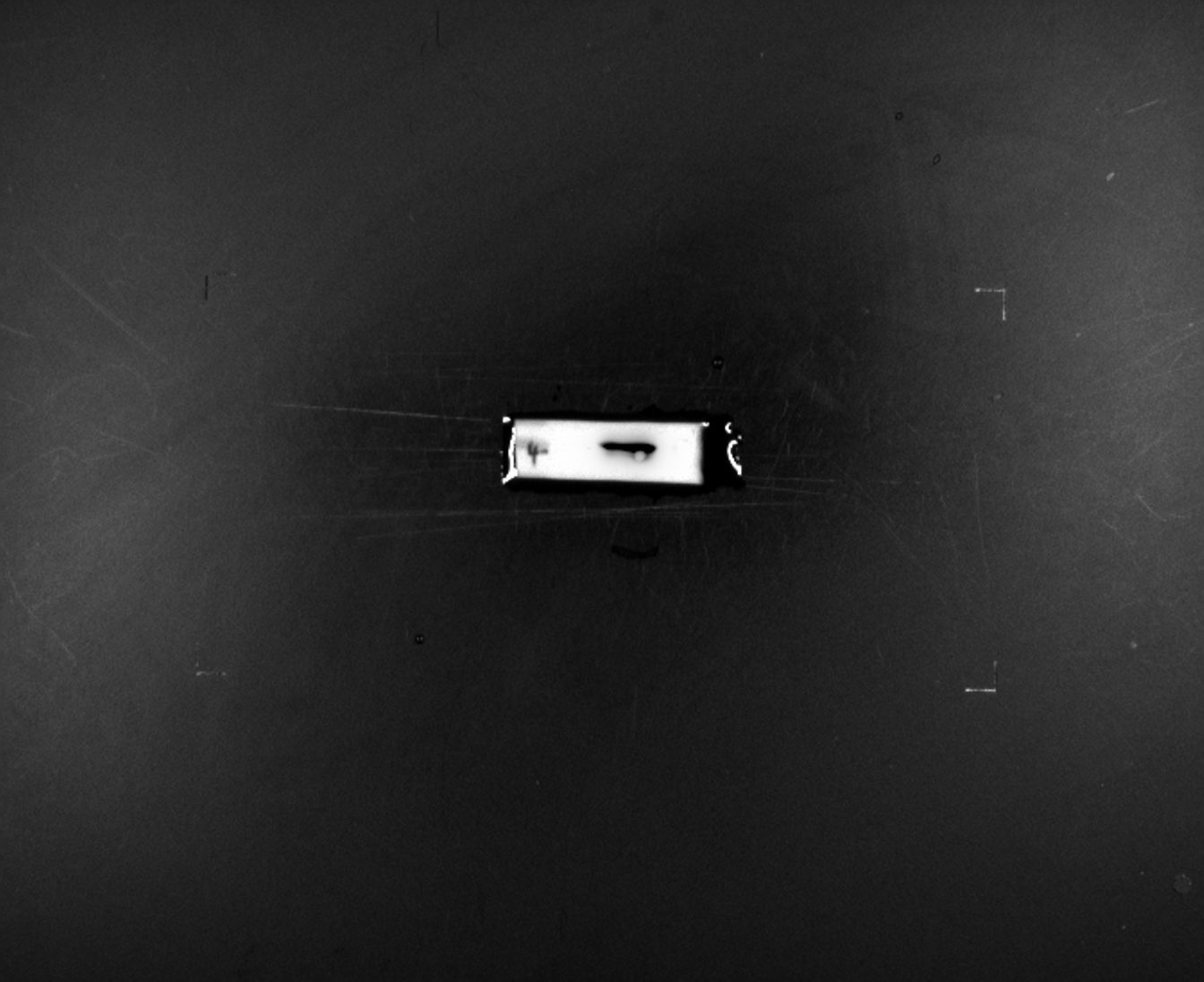


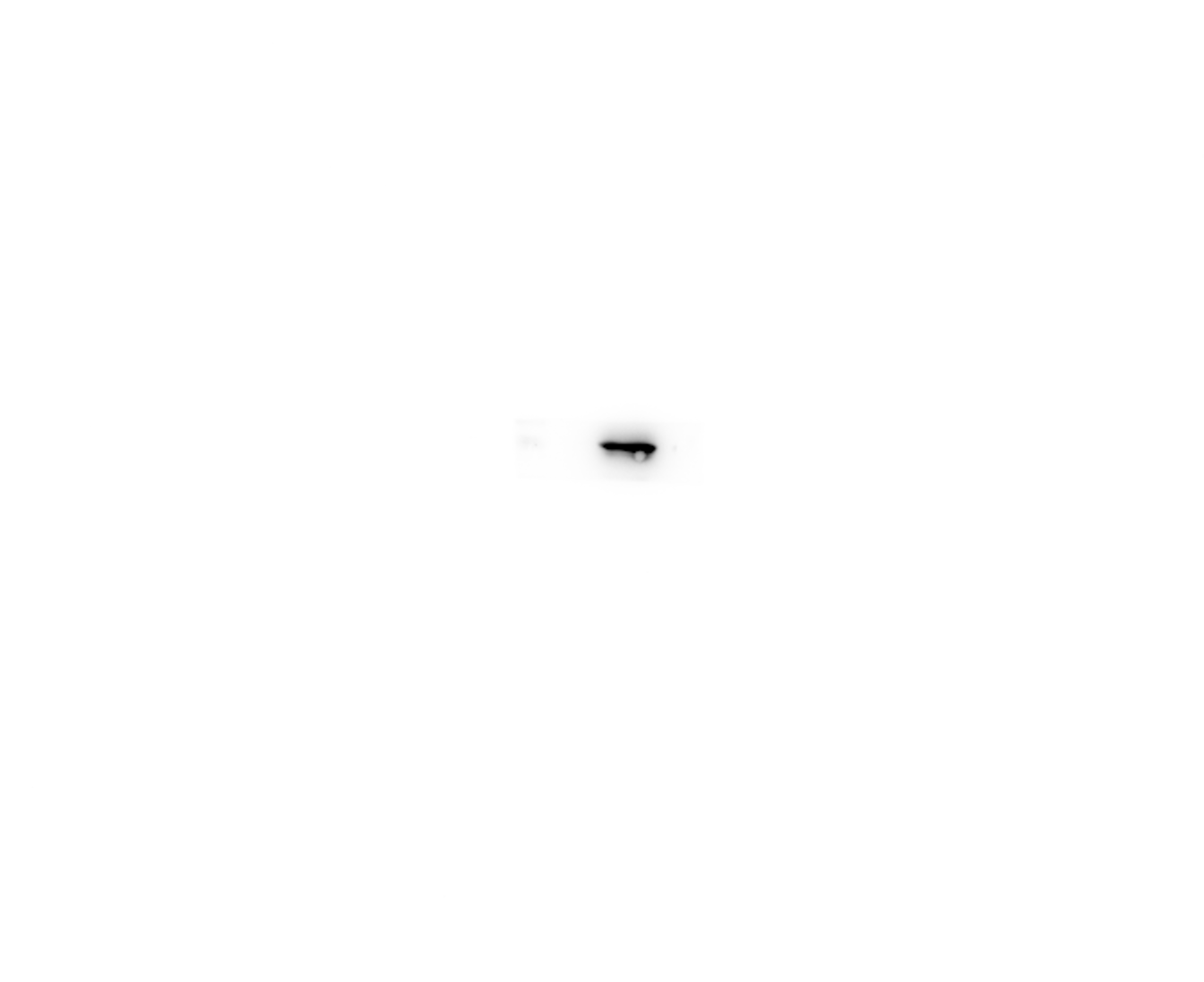

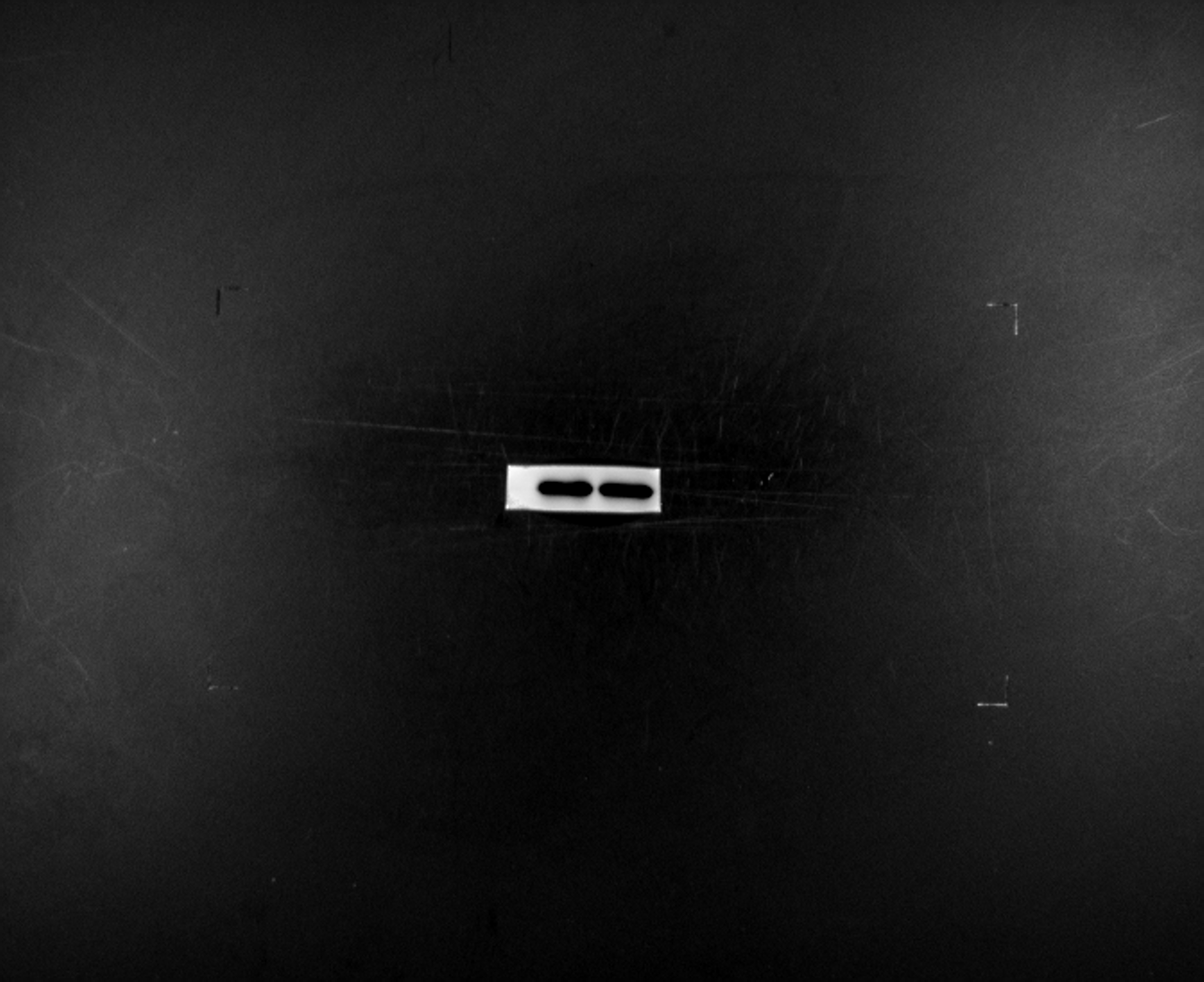

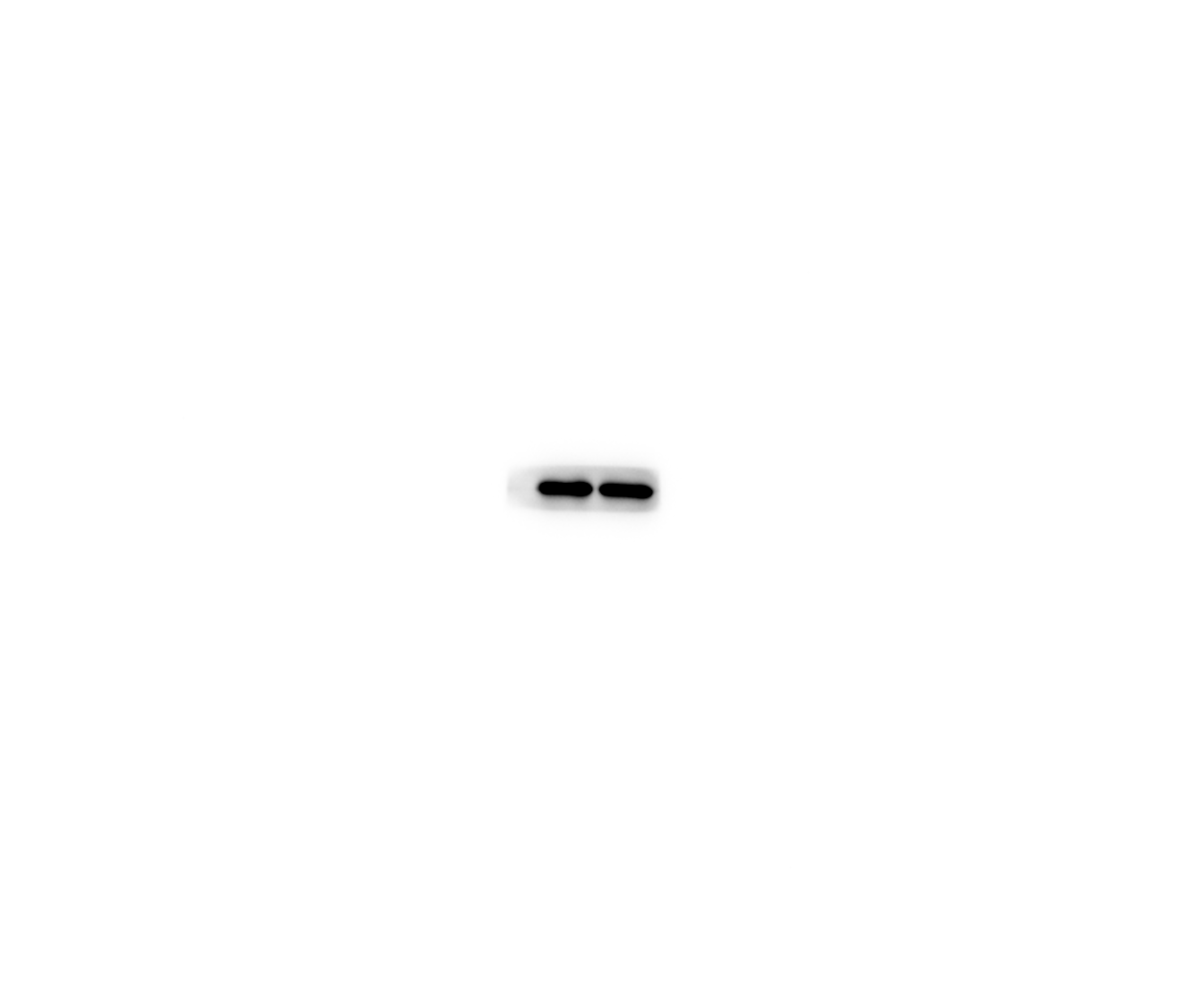


Fig 3E


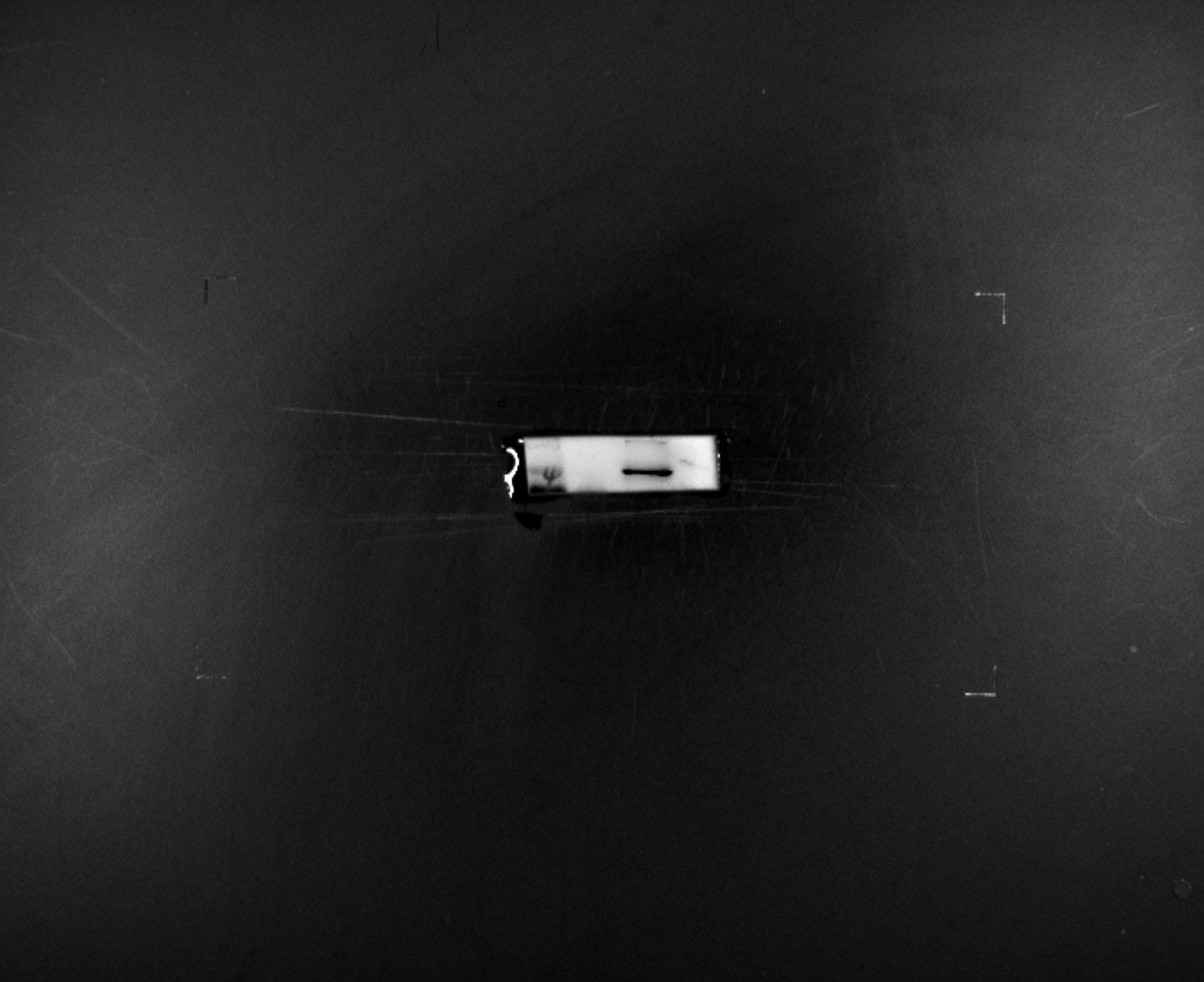

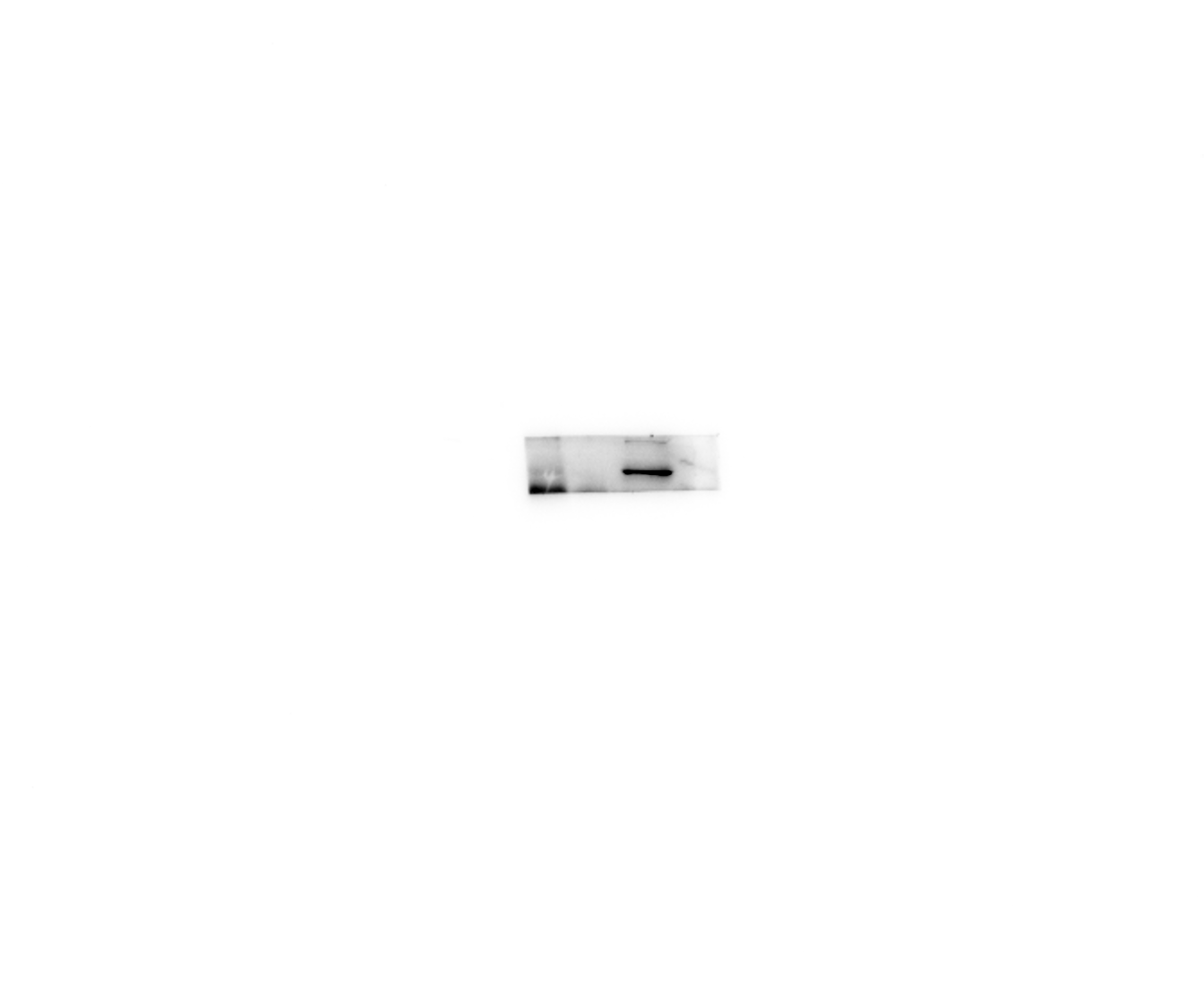

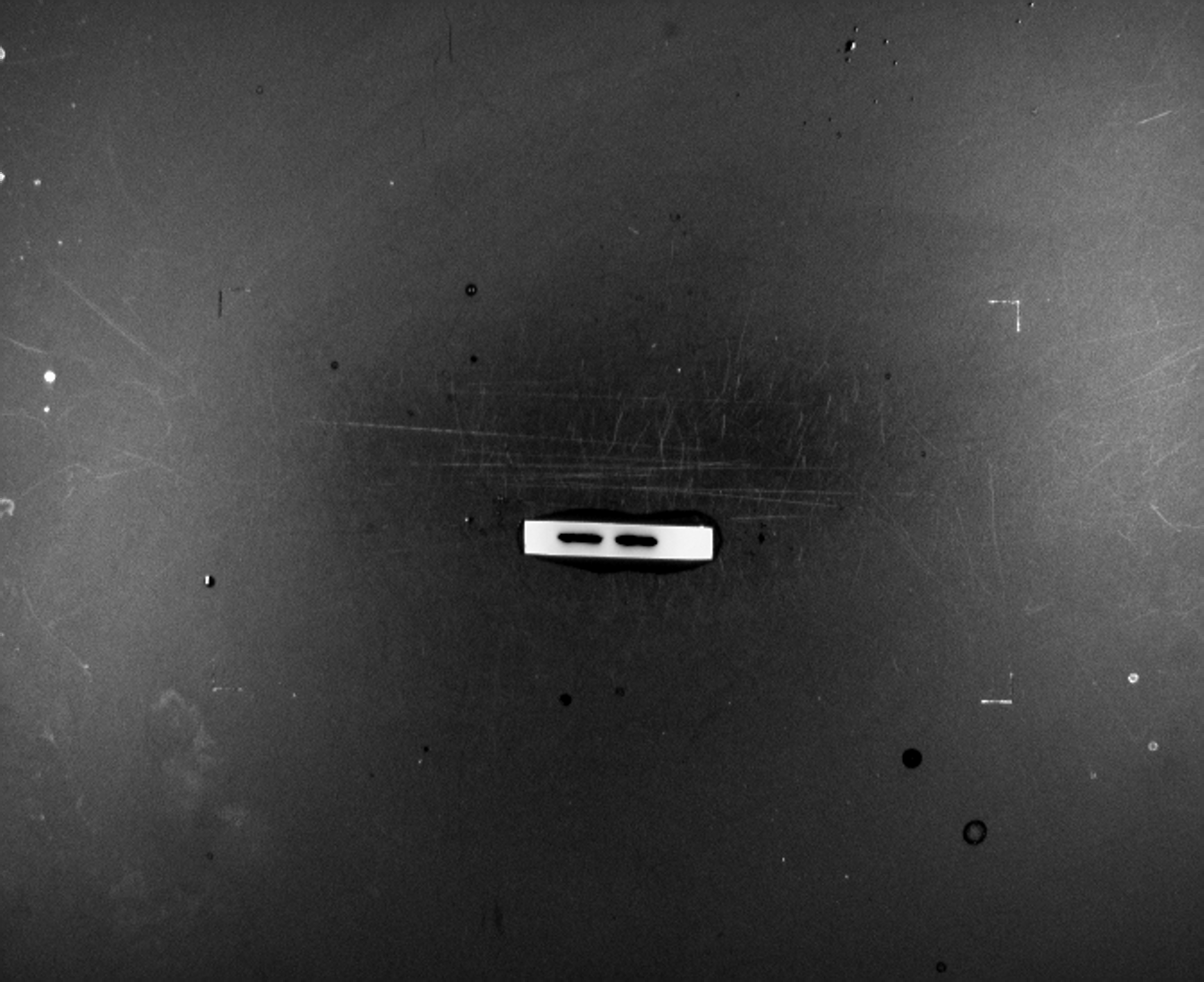

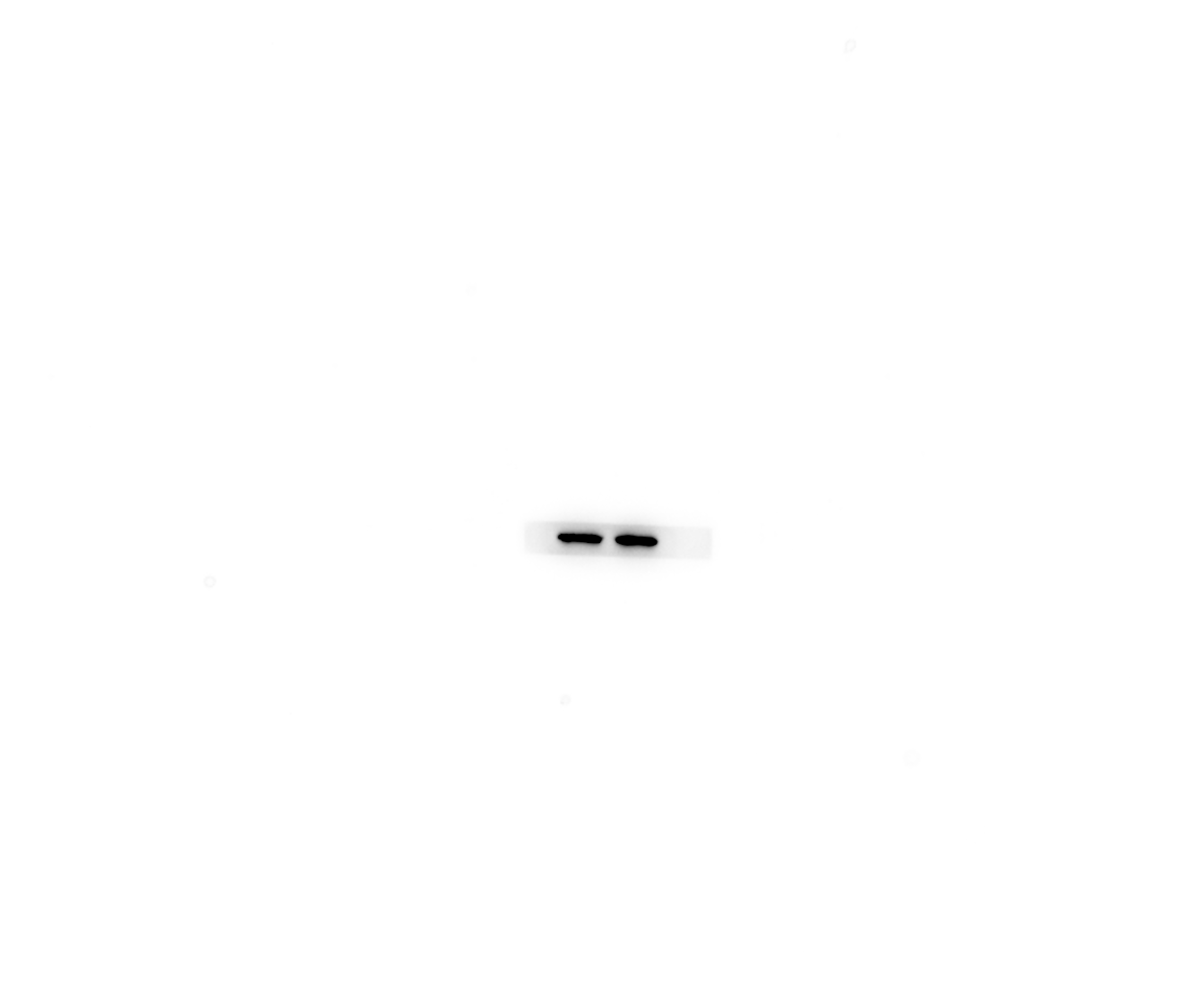


Fig 3I


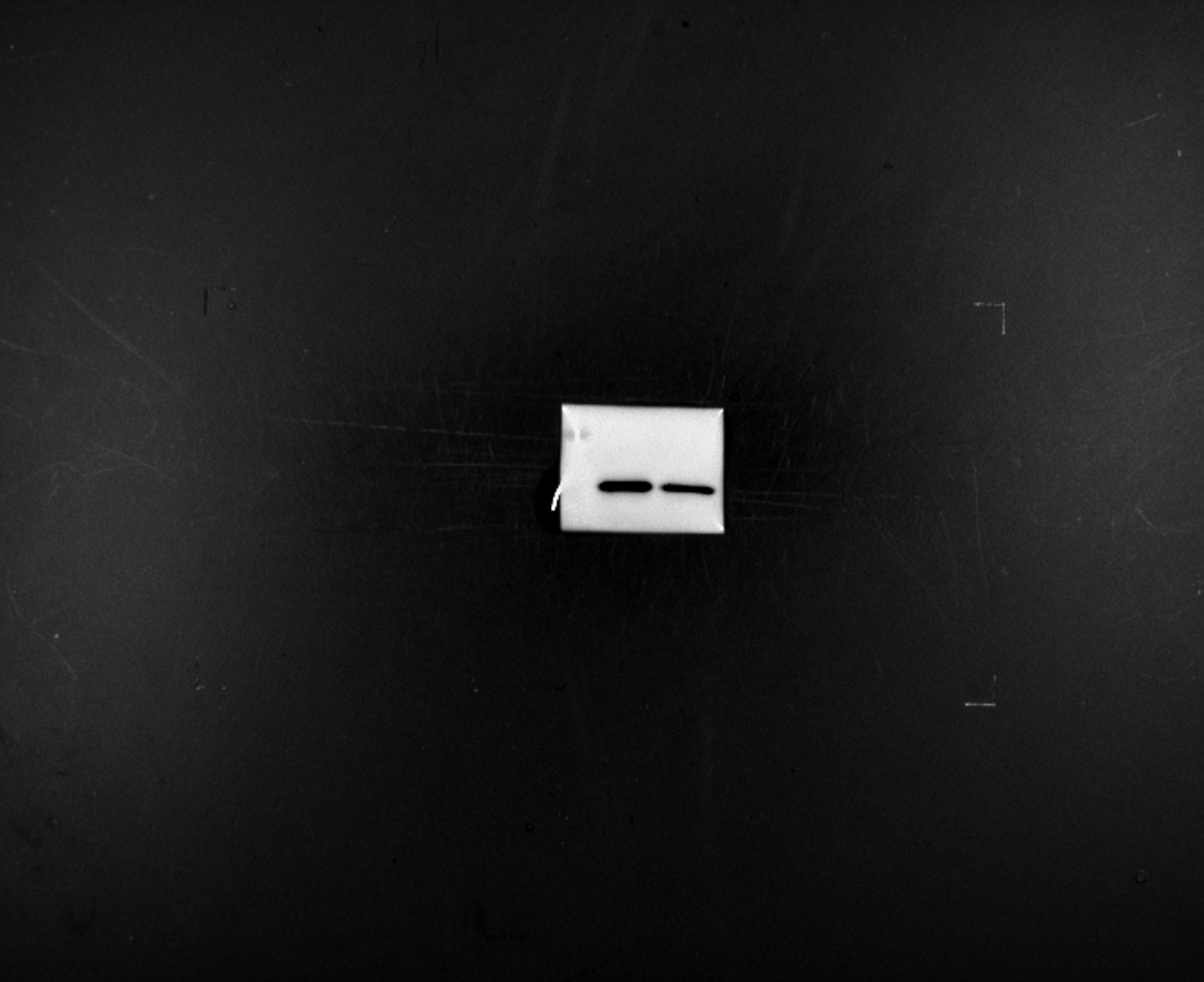


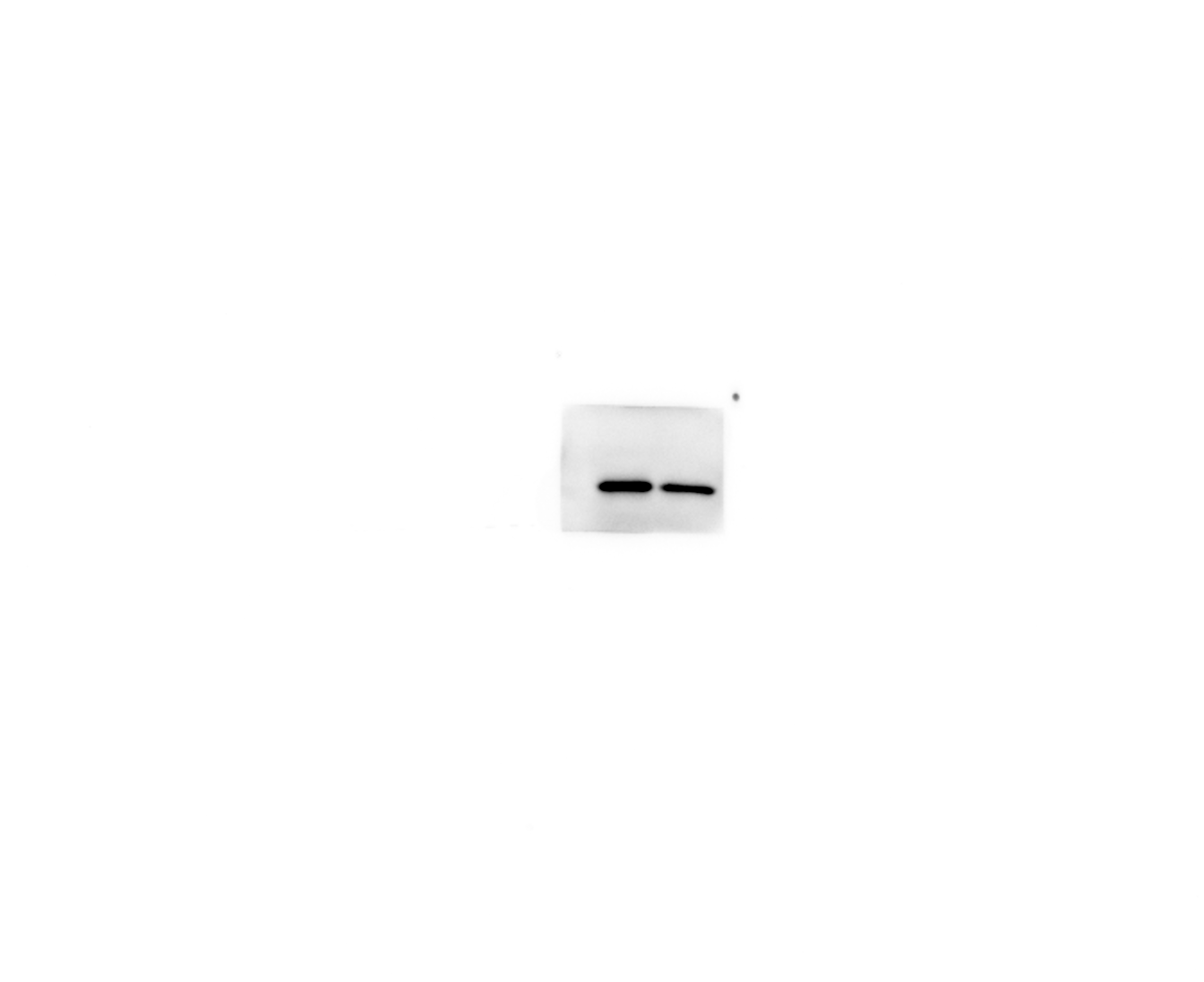

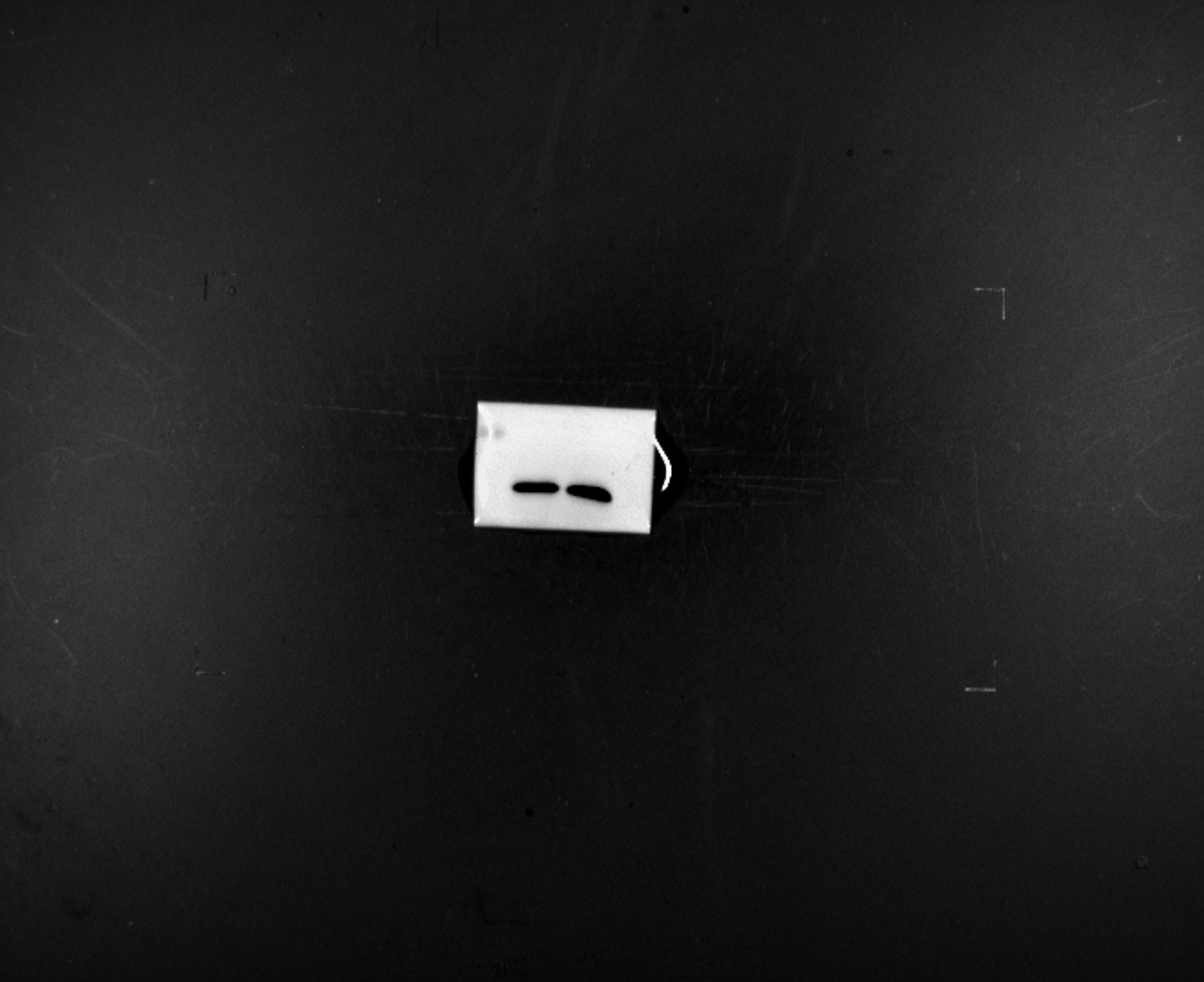

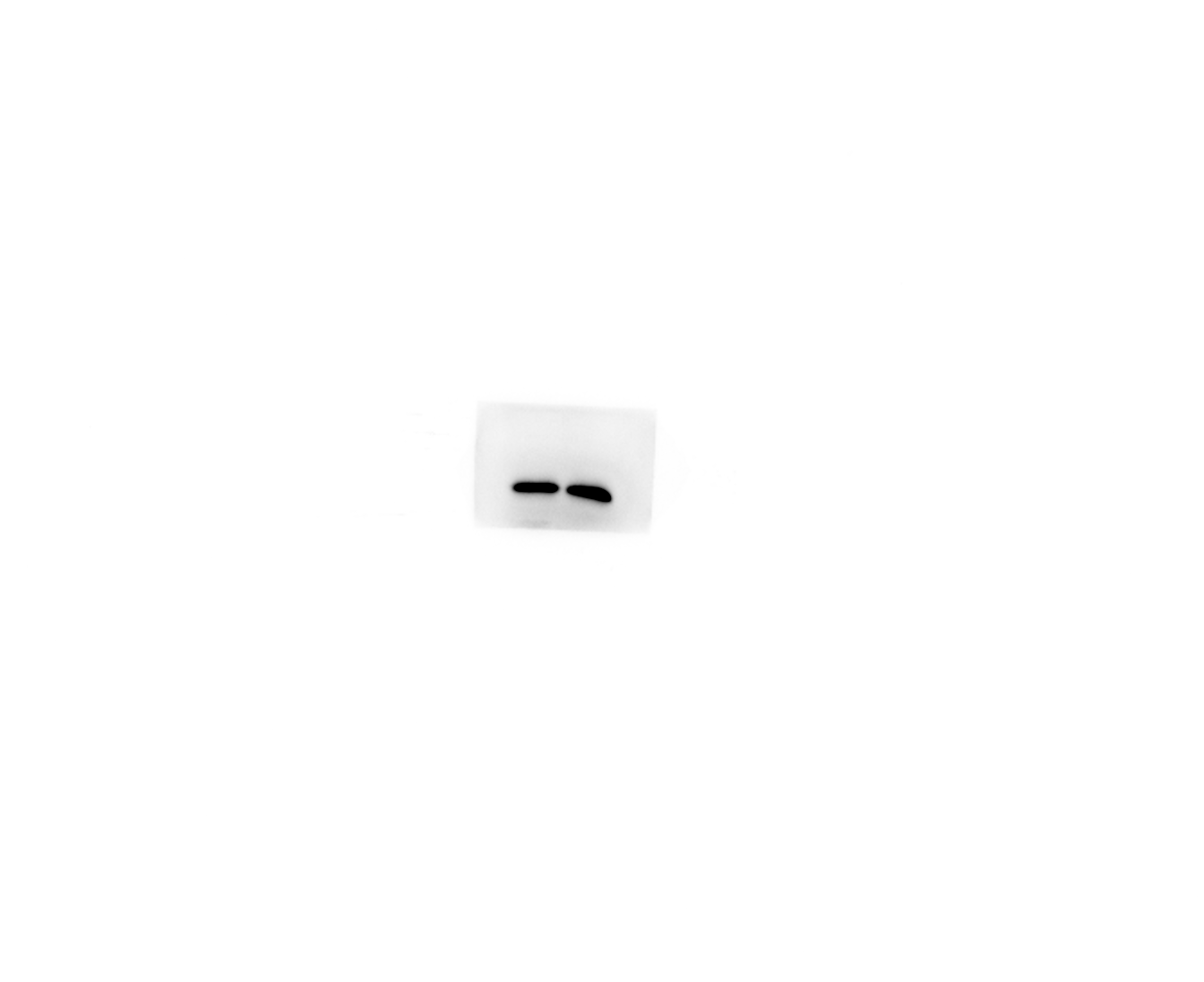


Fig 3J


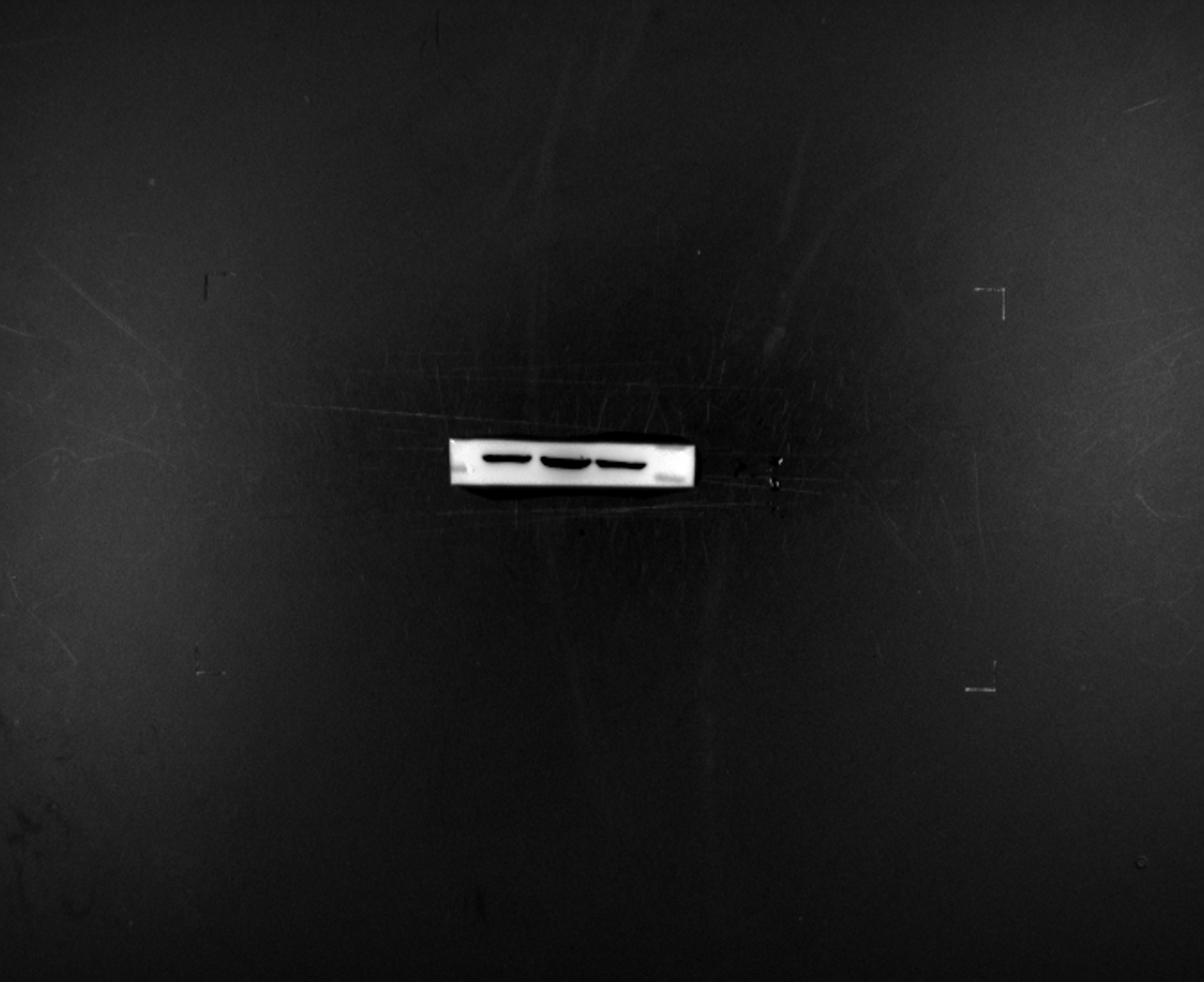

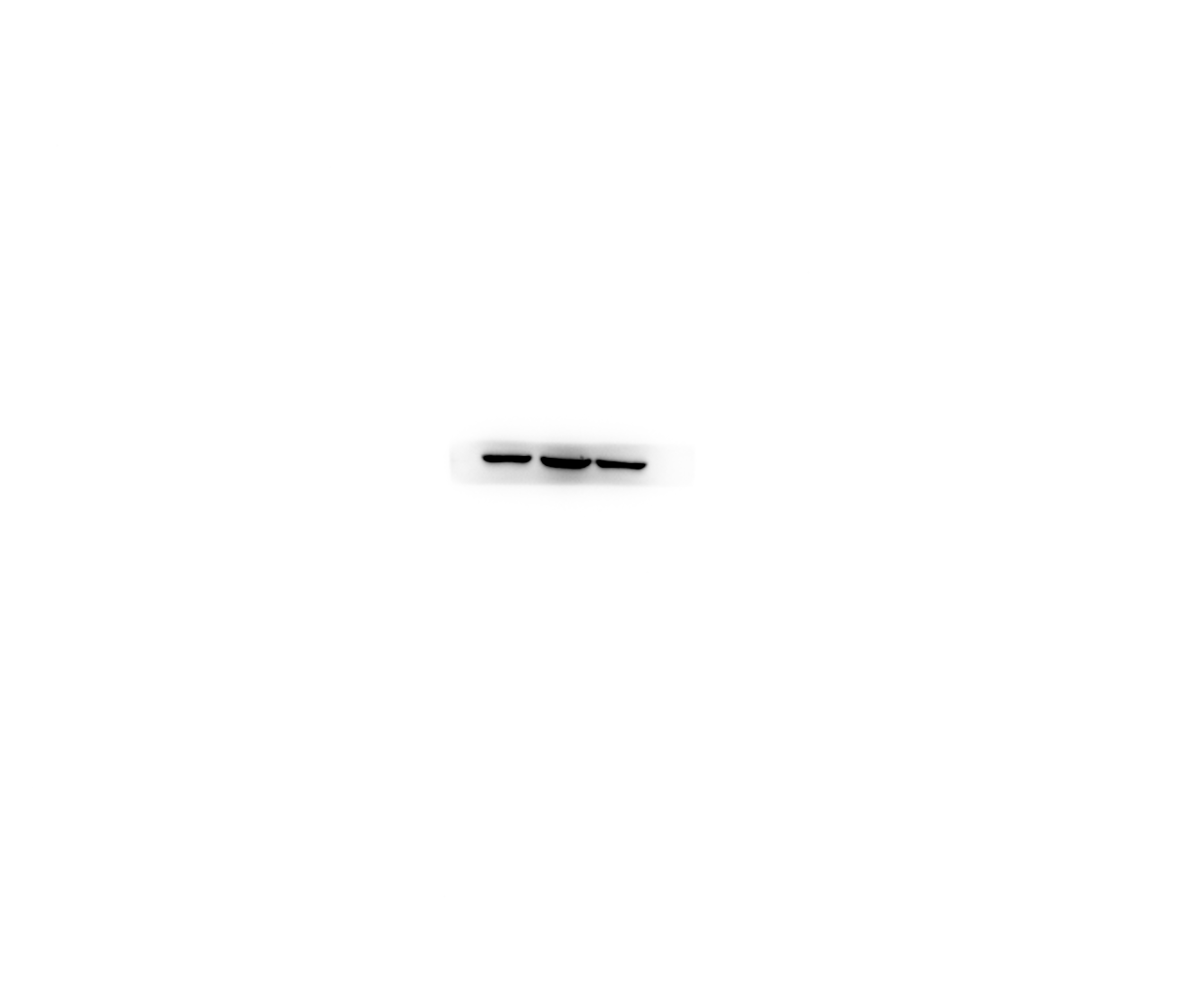

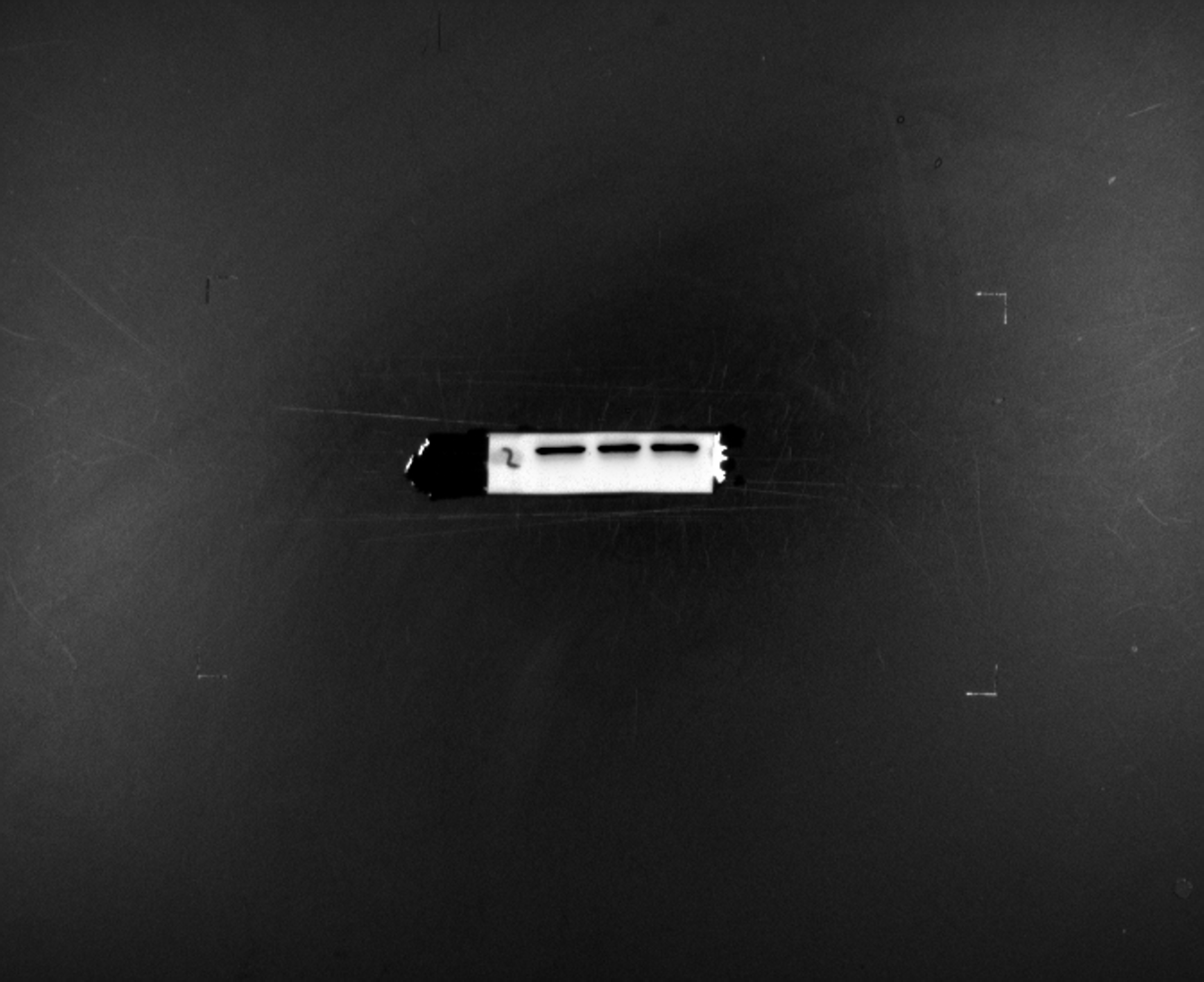

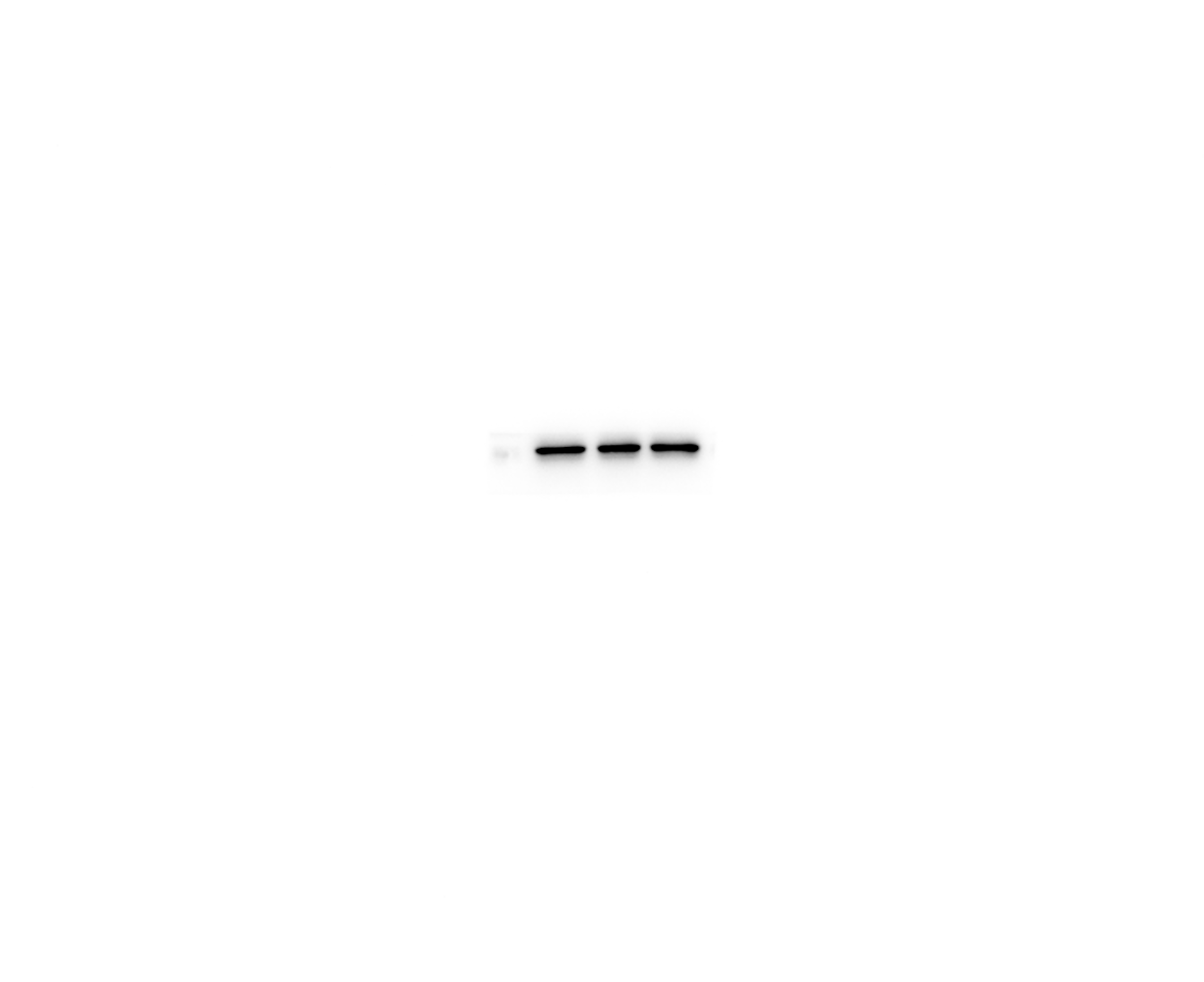


Fig 4C


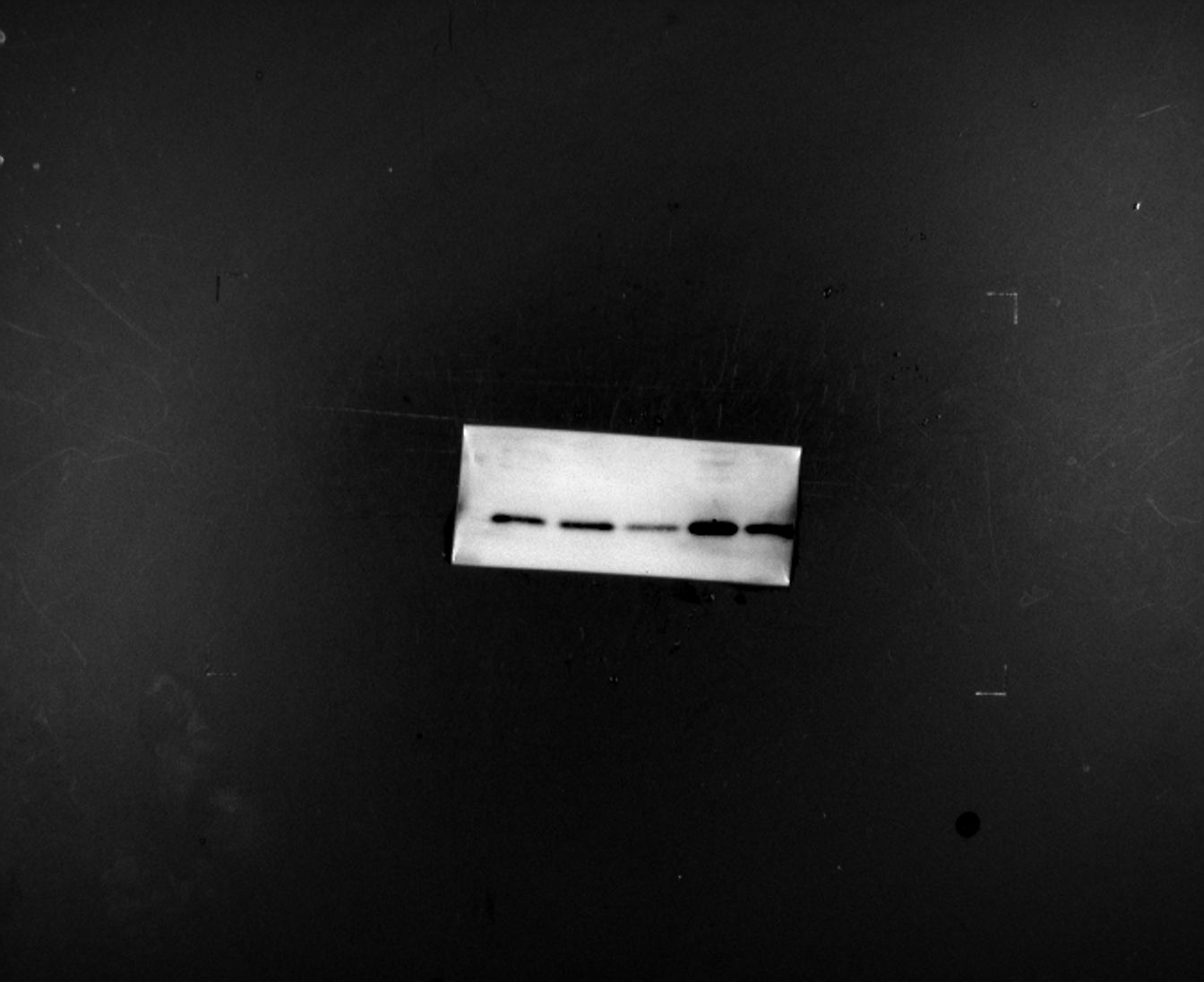

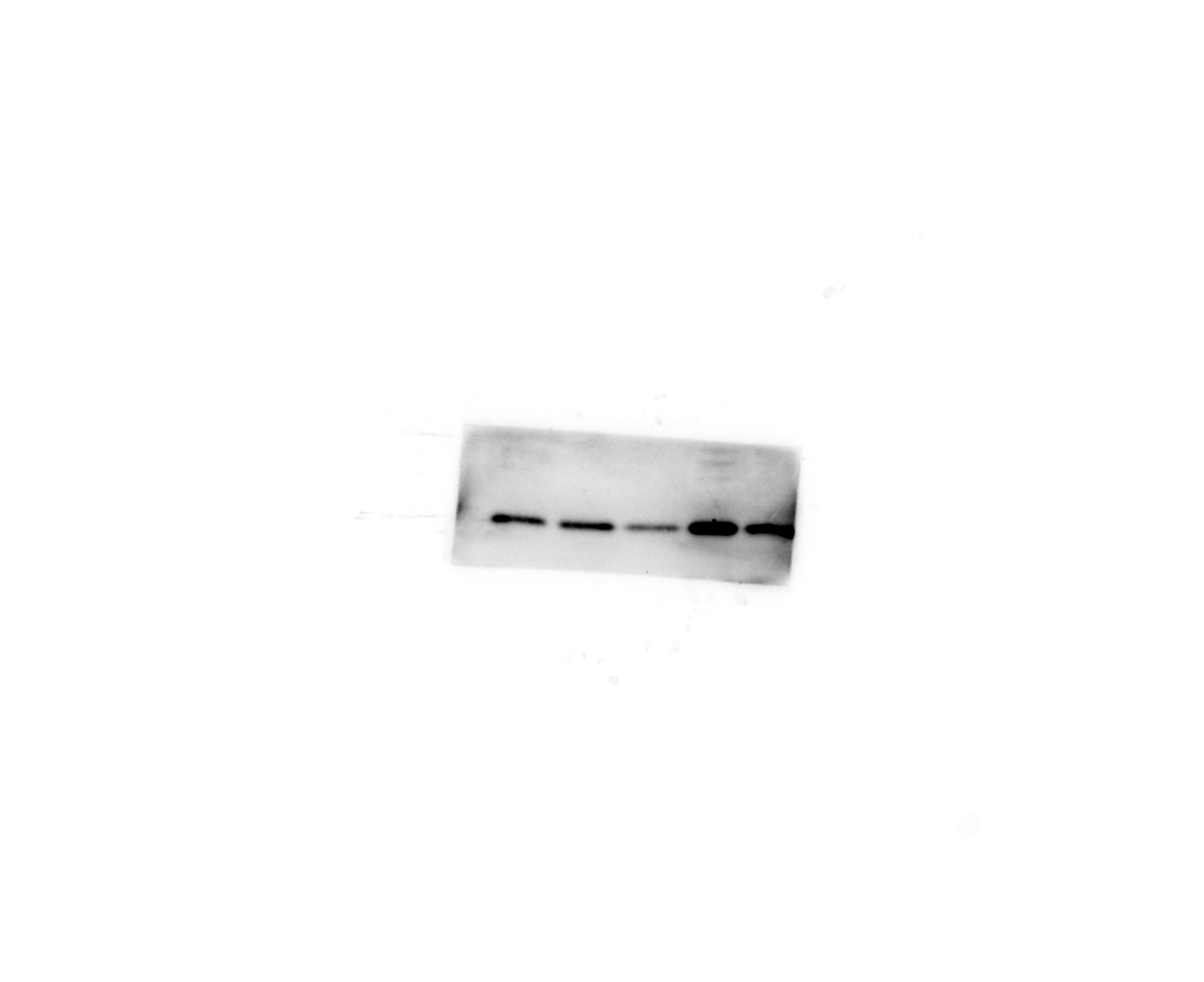

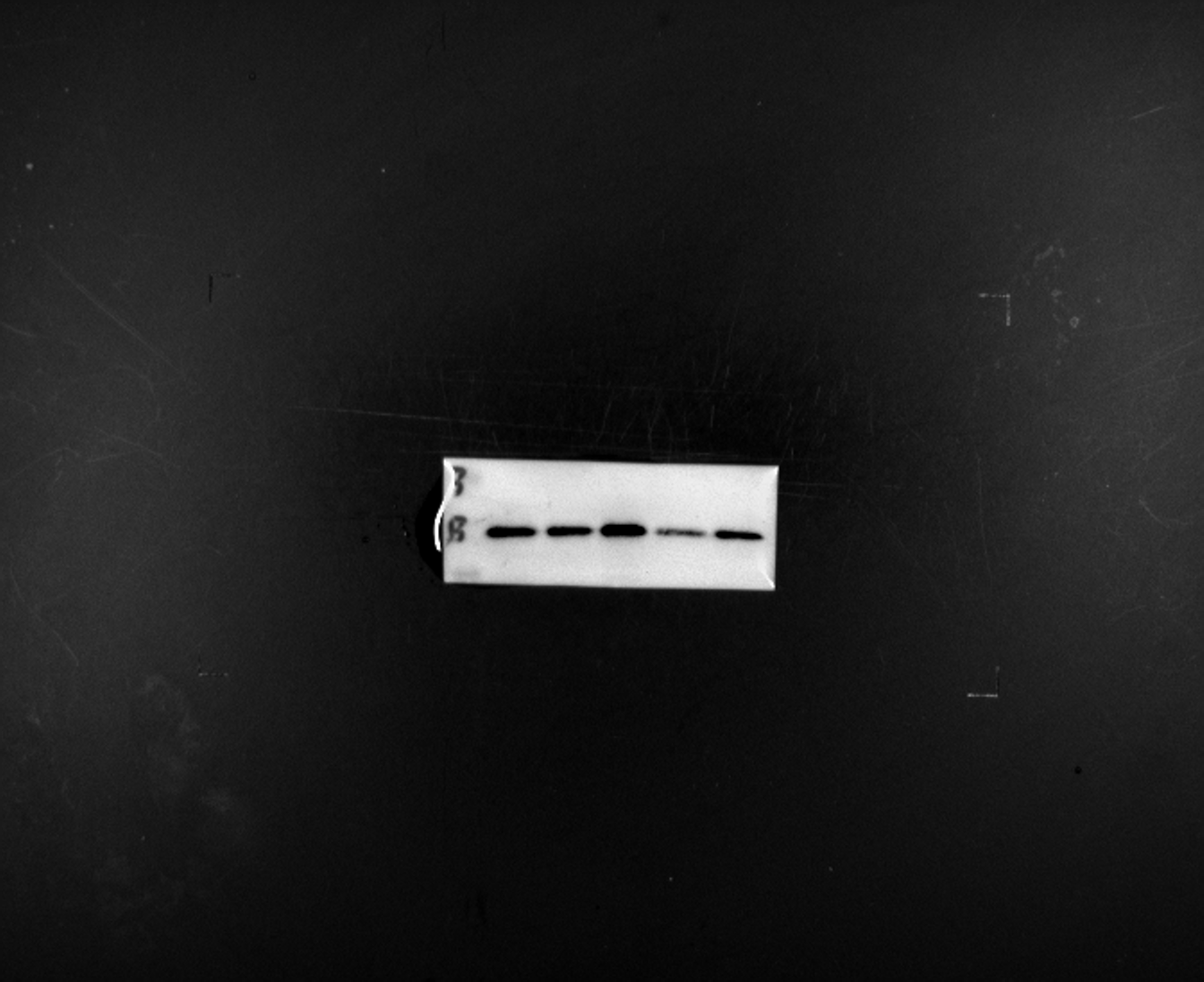

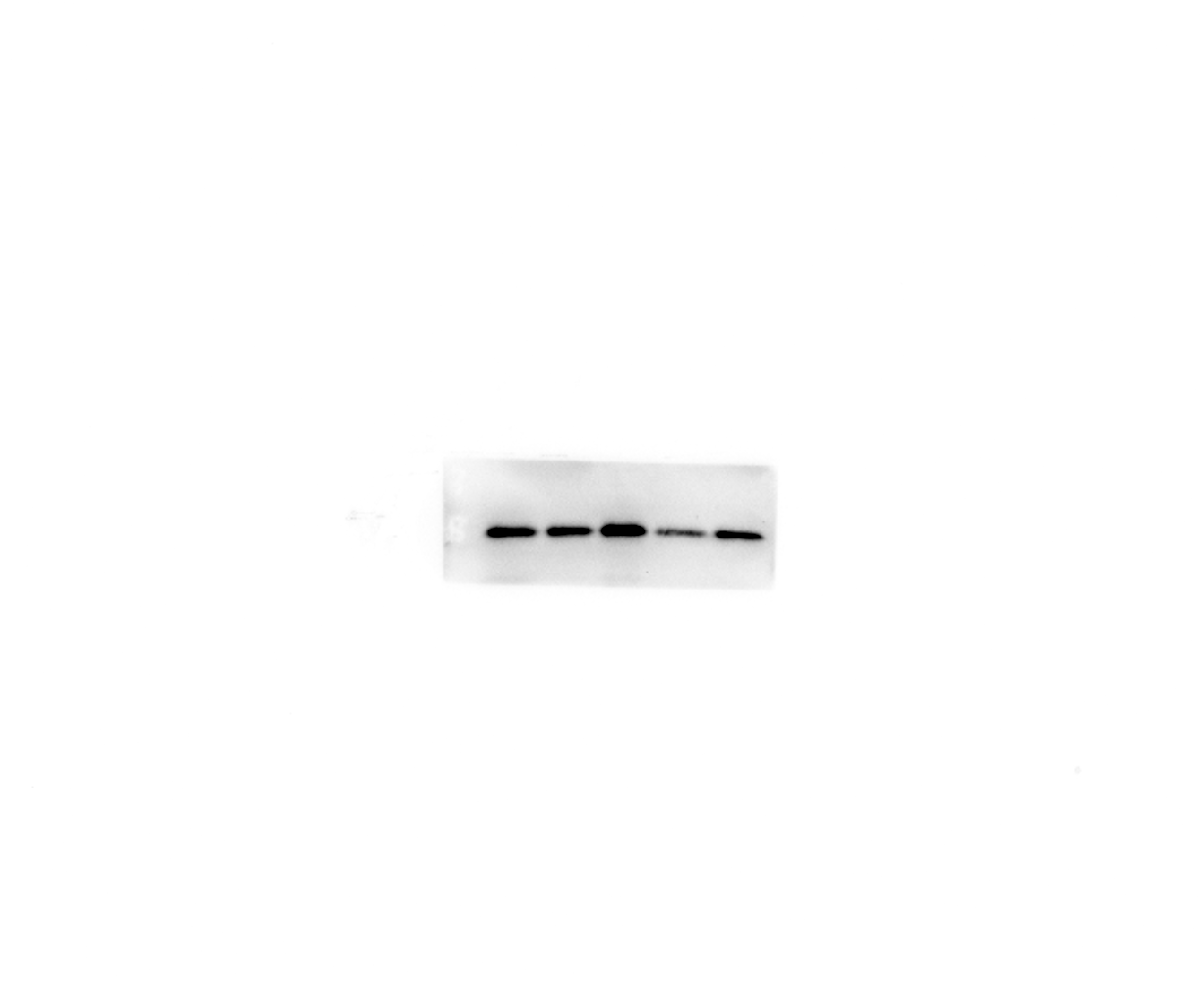

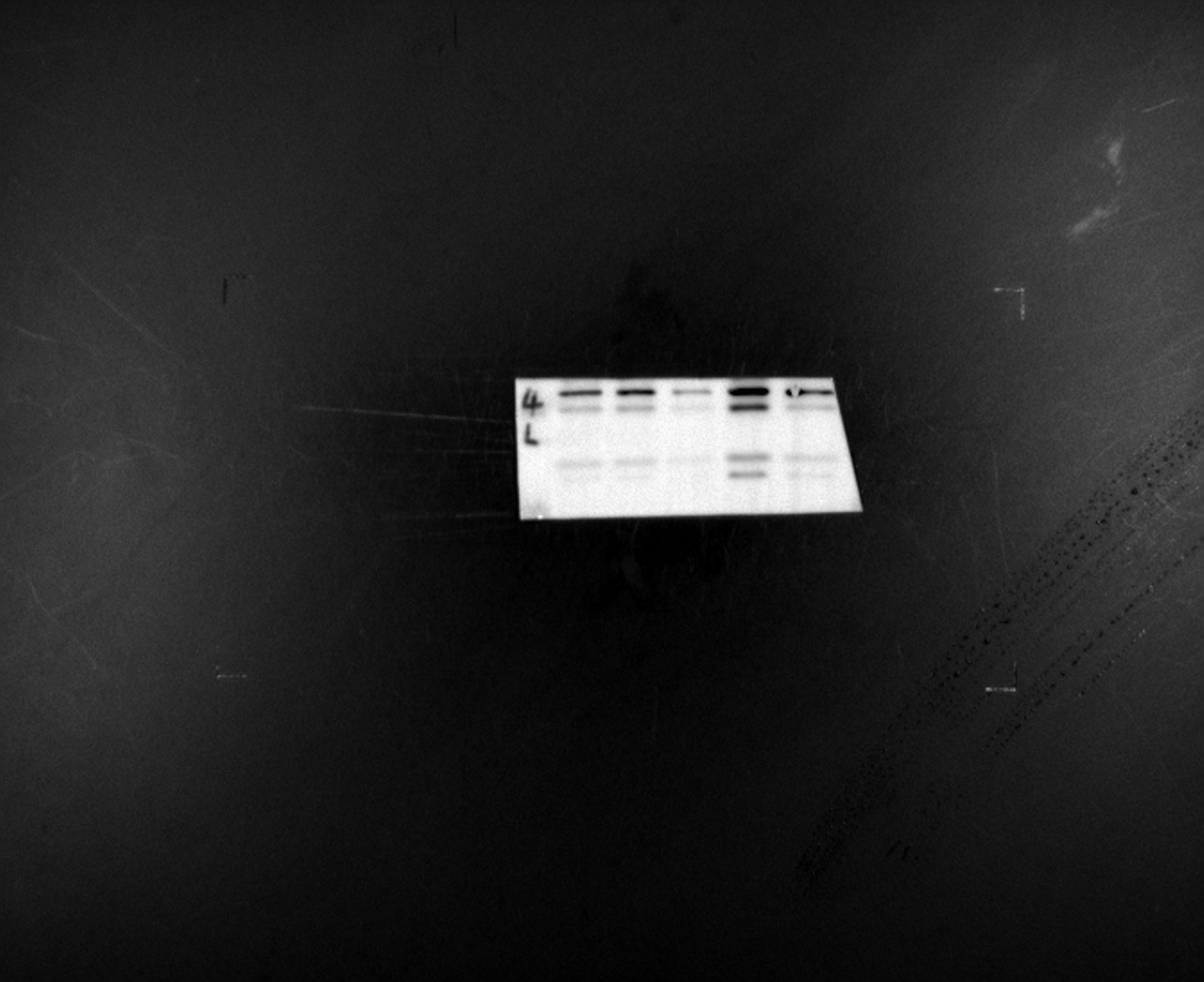

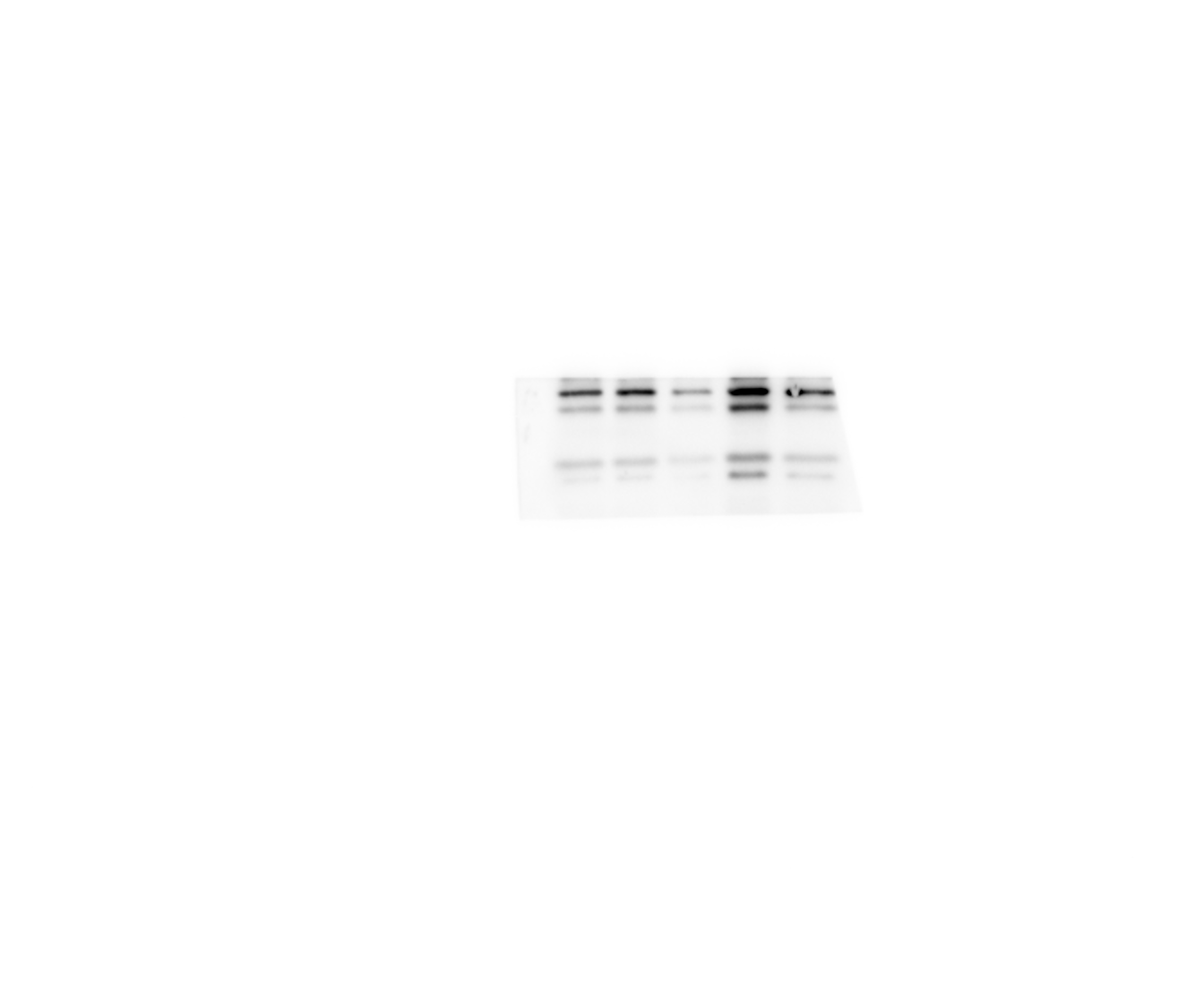


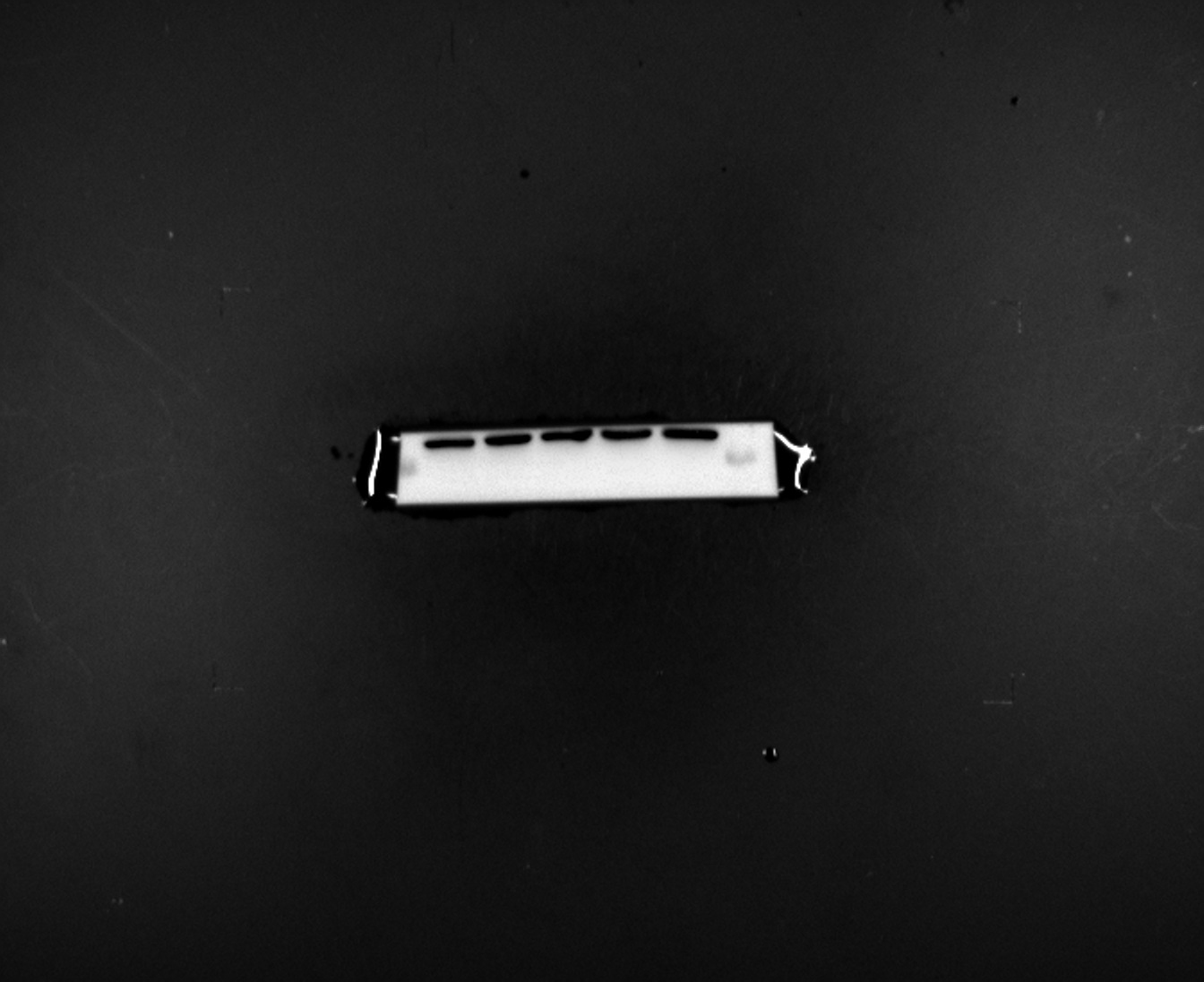


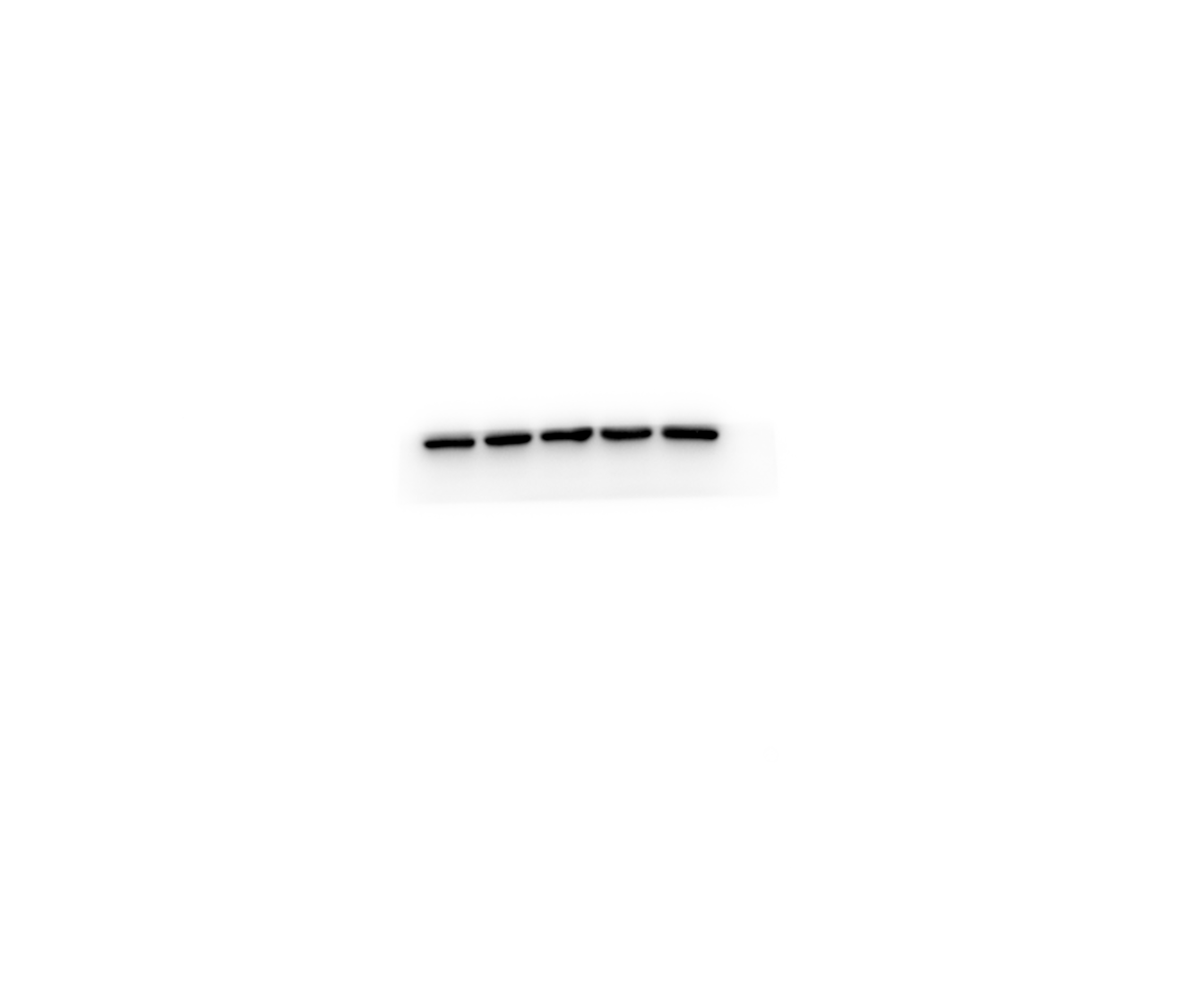

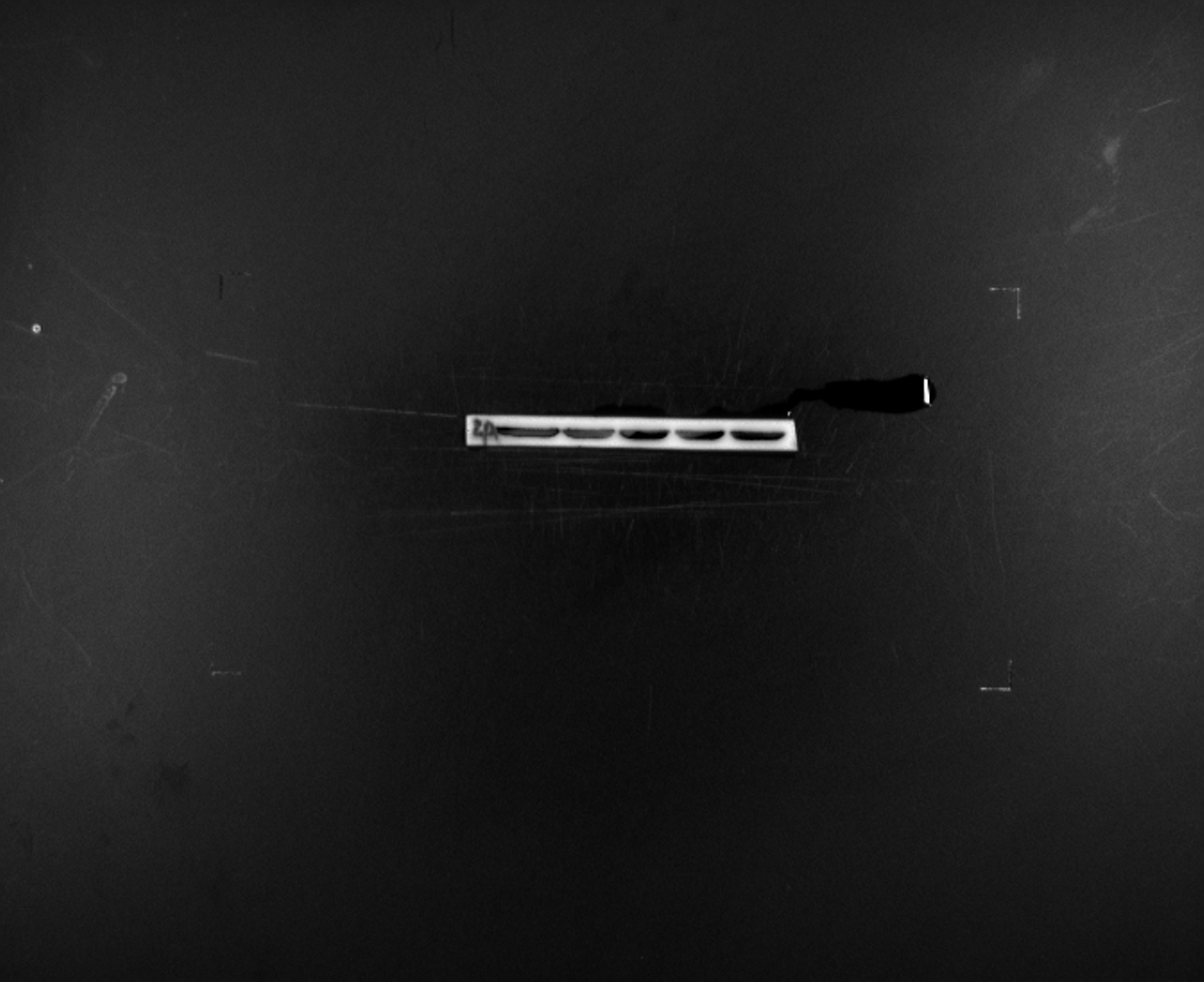

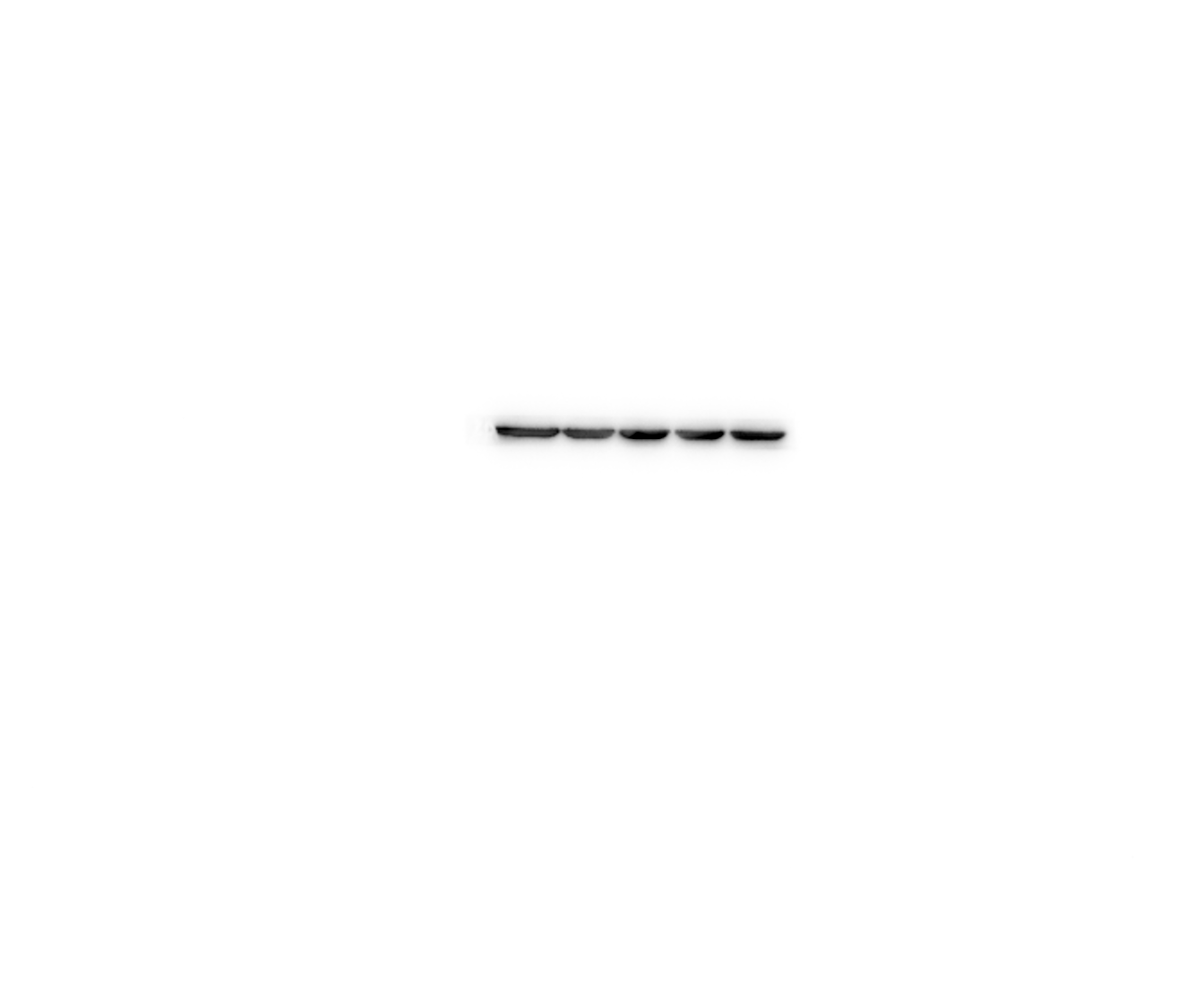


Fig 6E


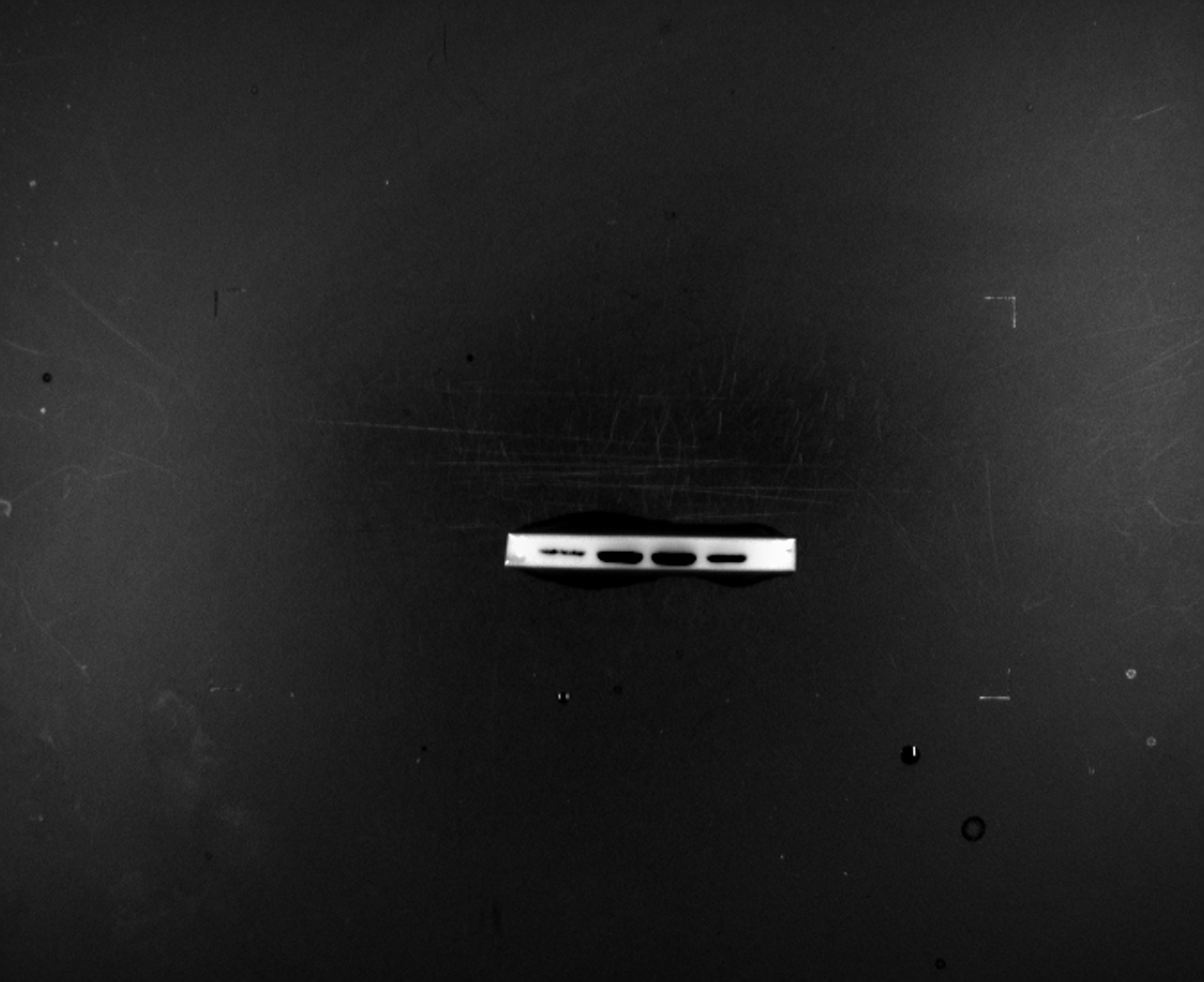

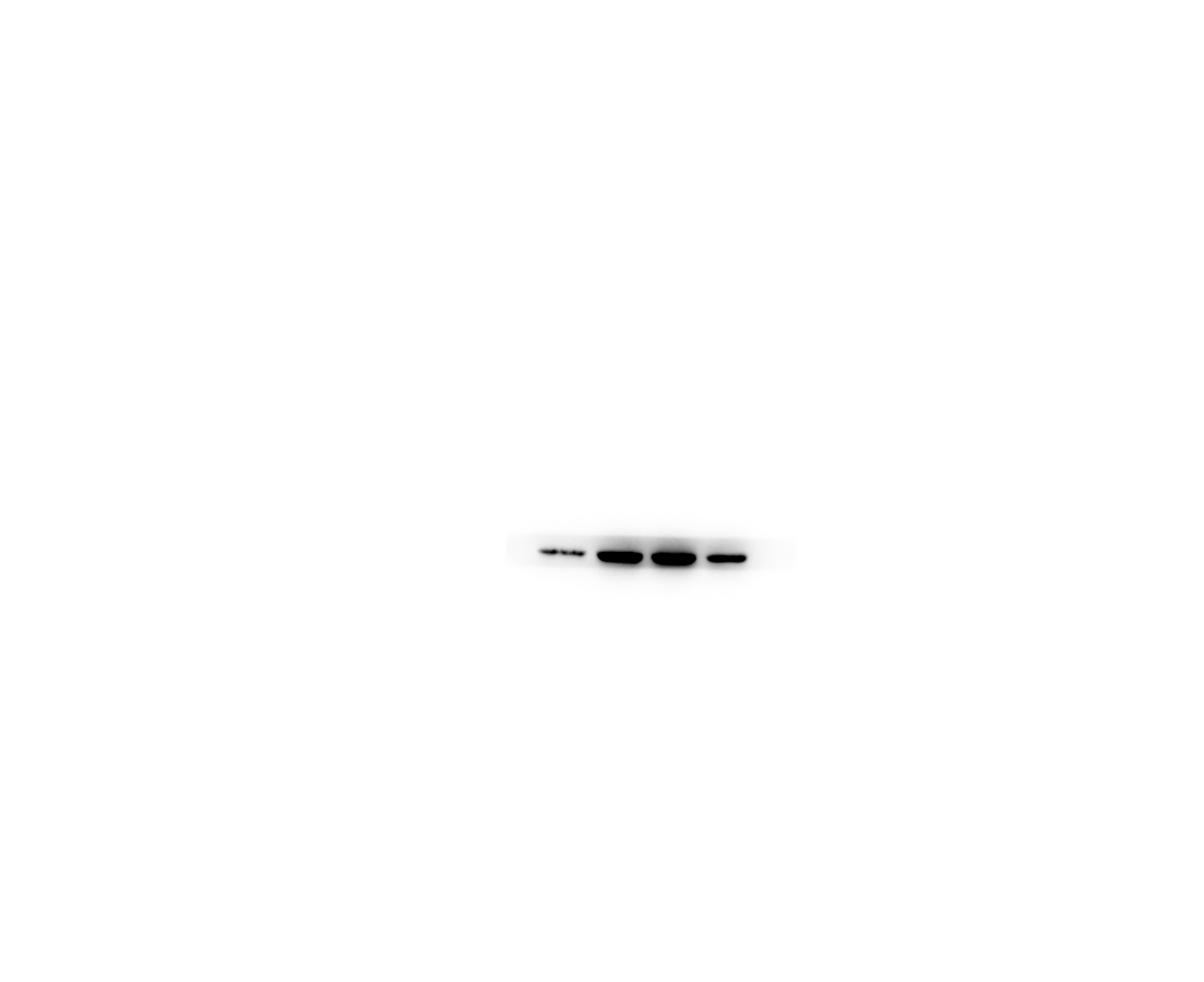

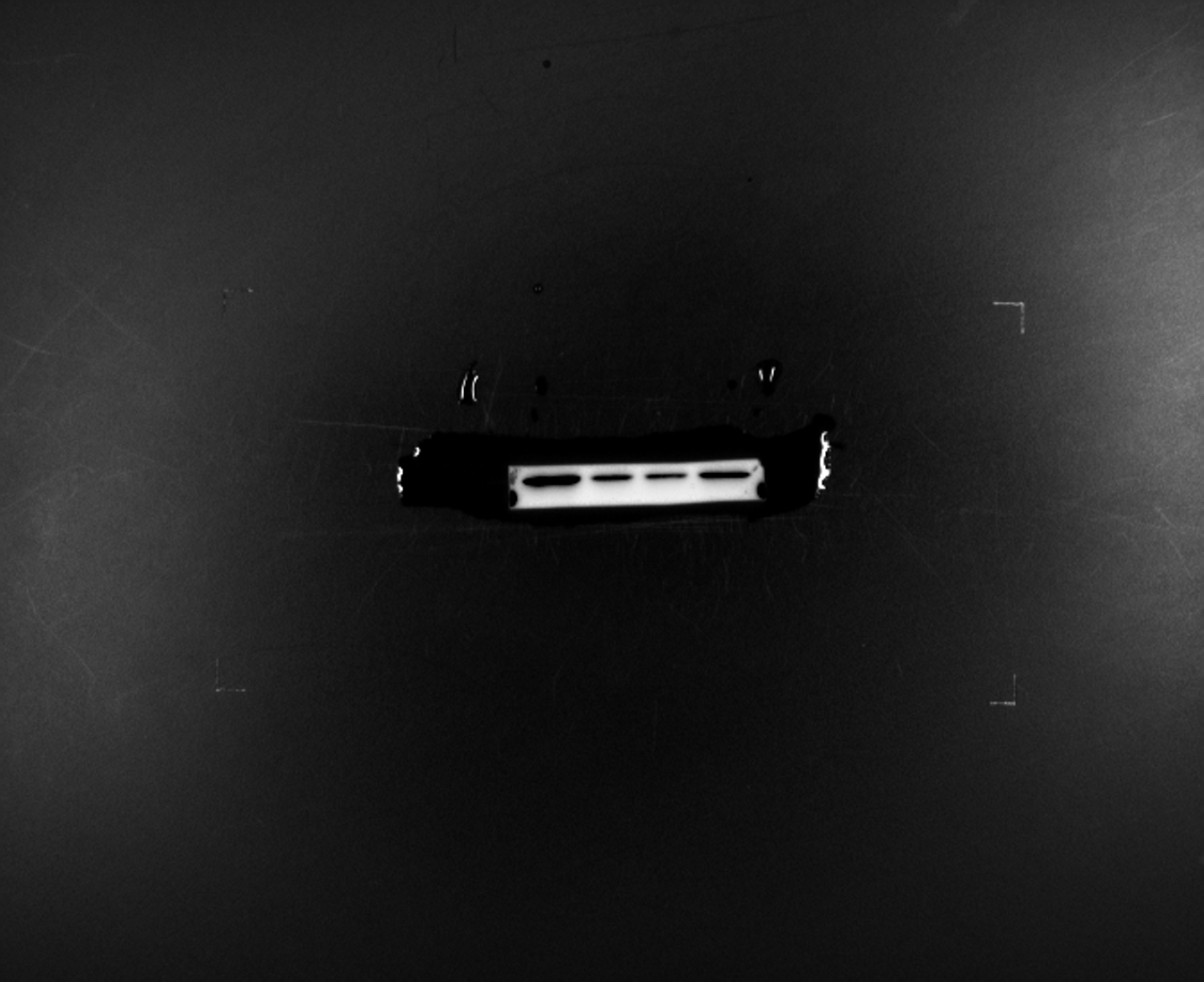

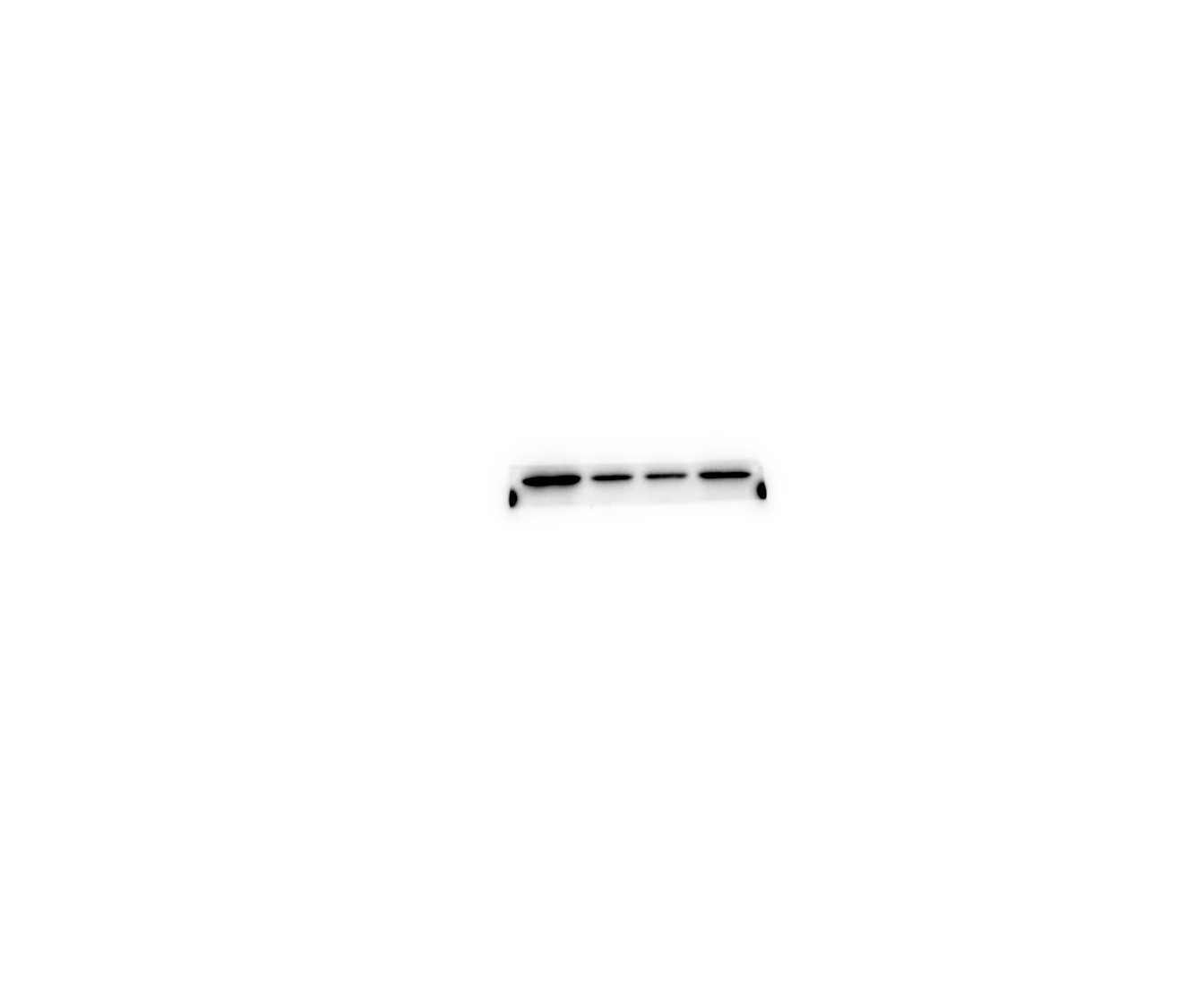

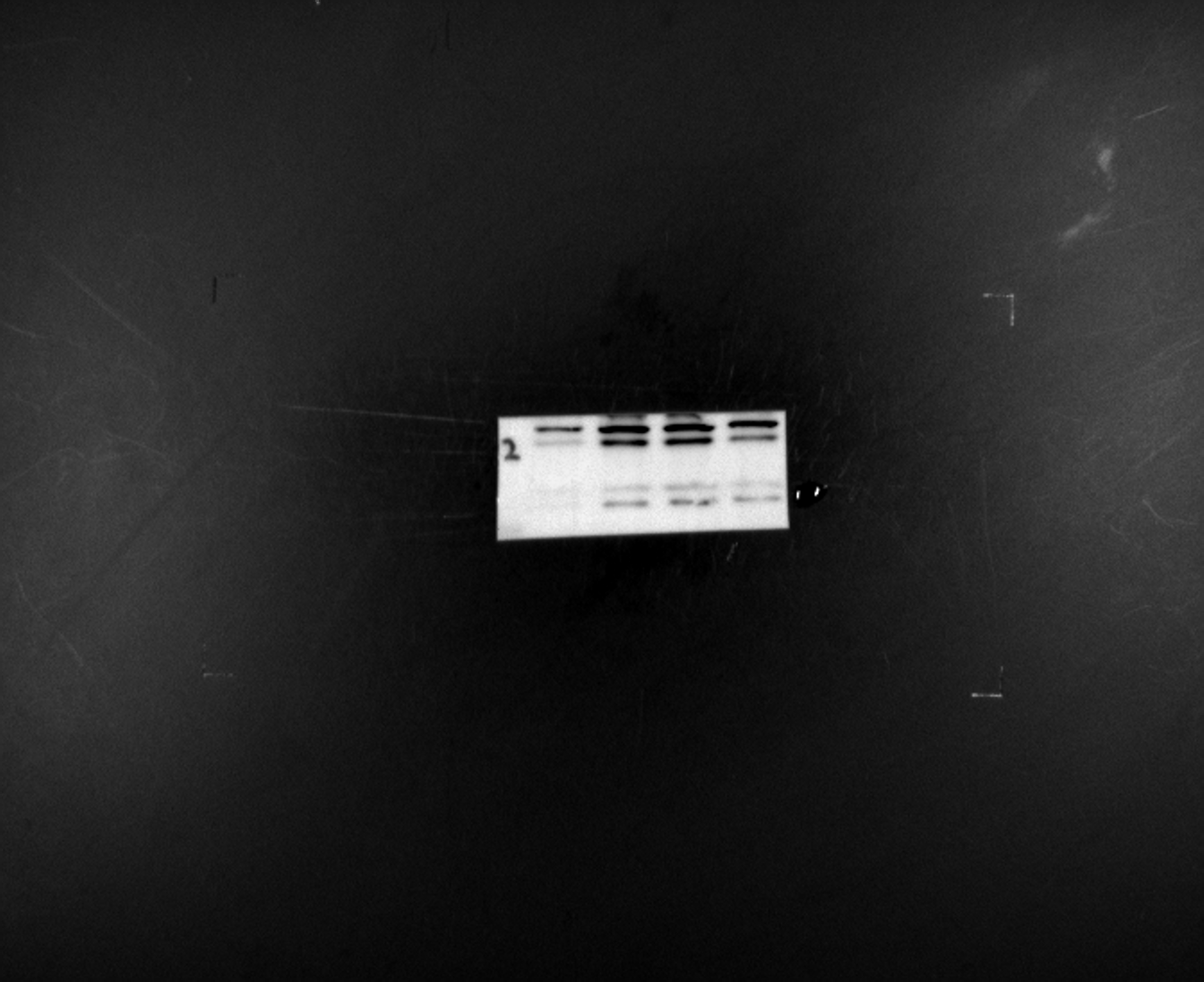

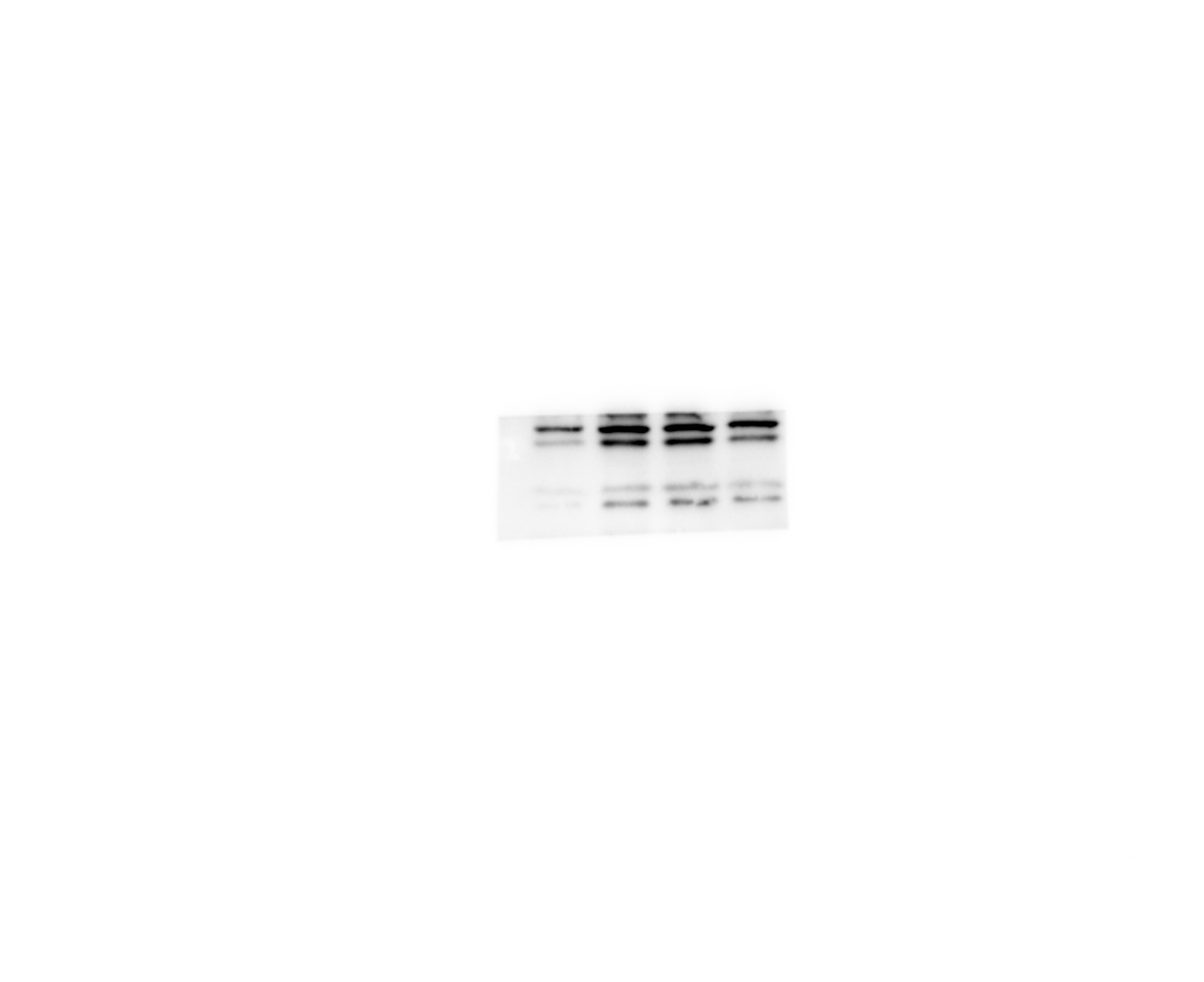

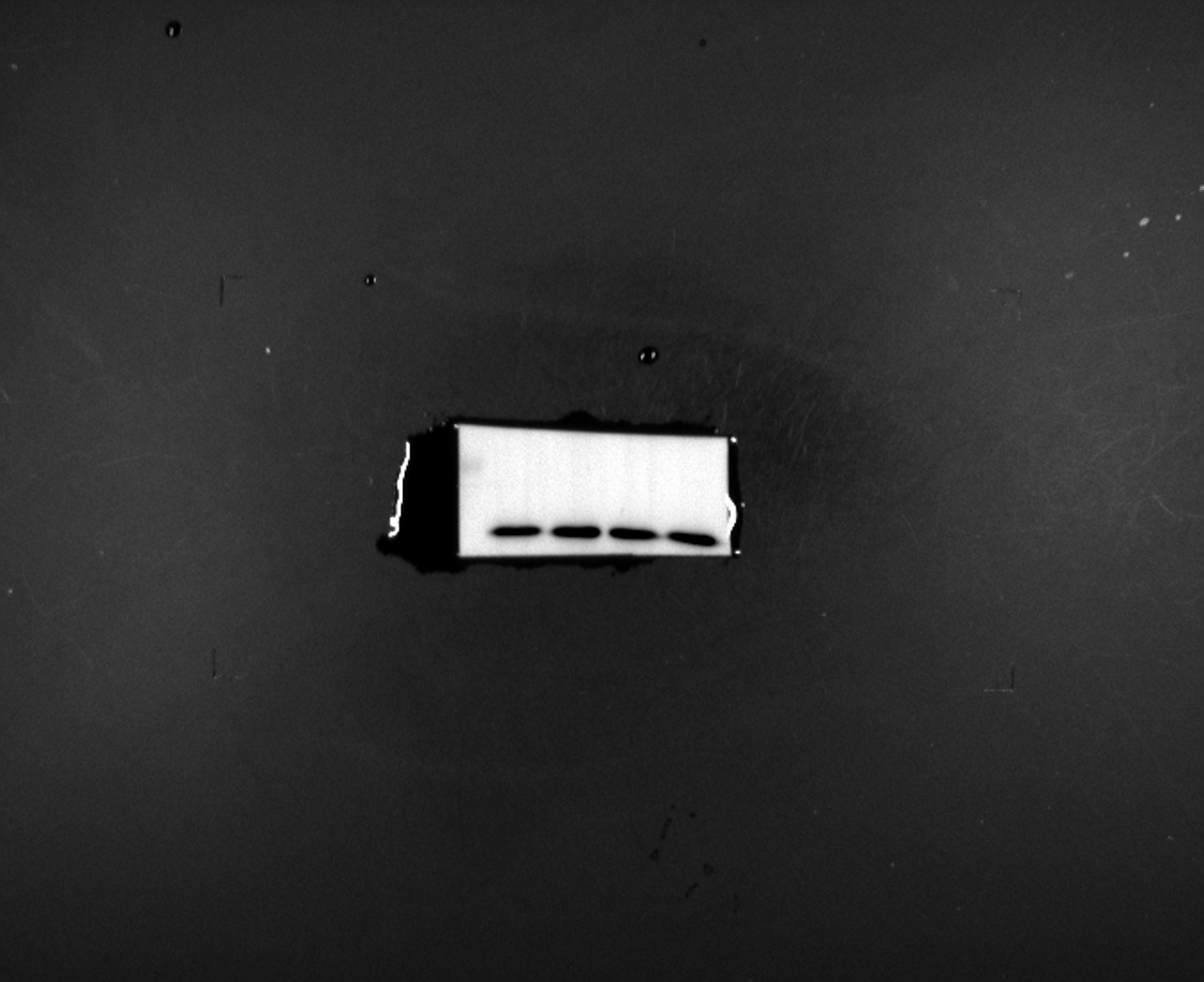

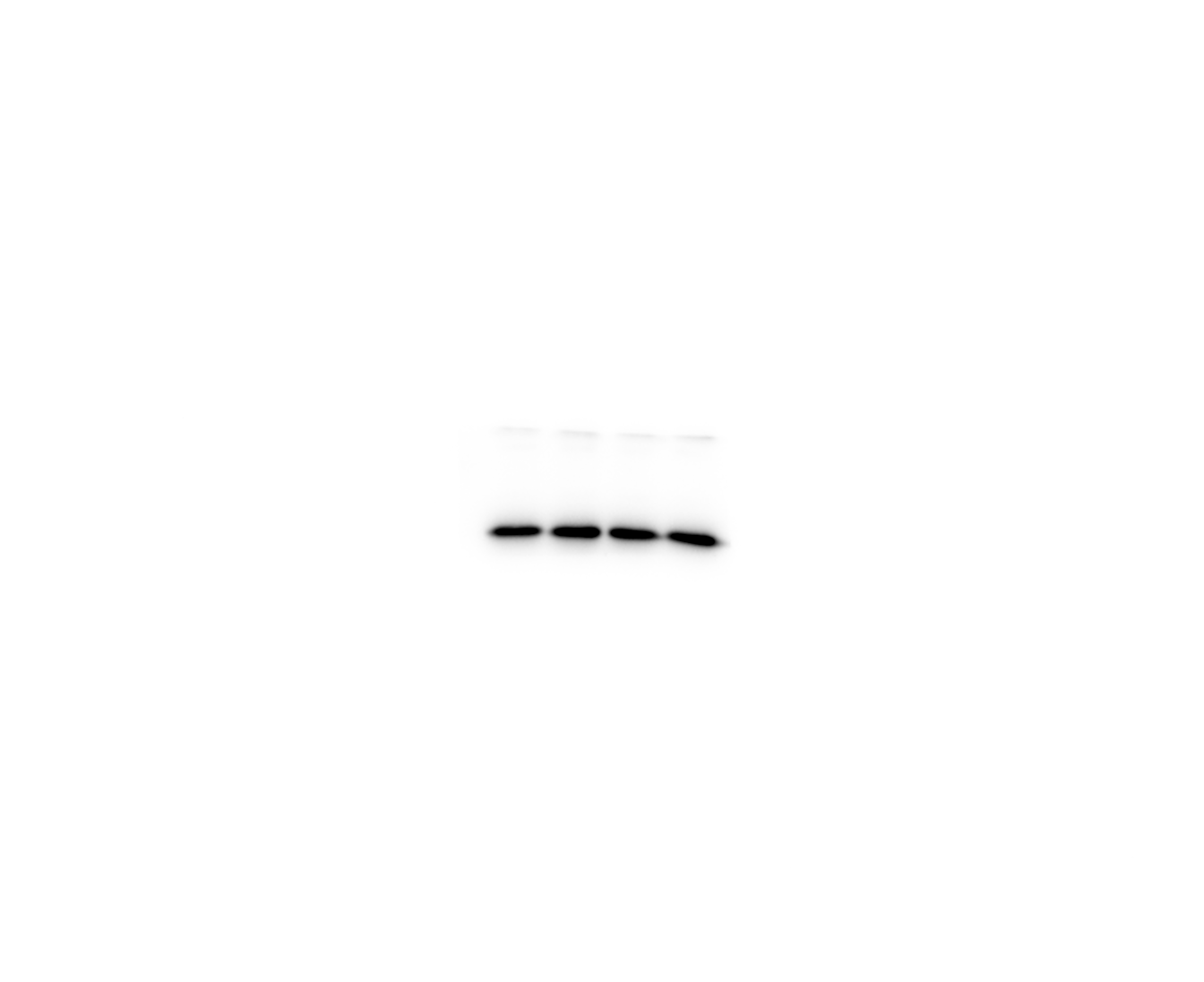


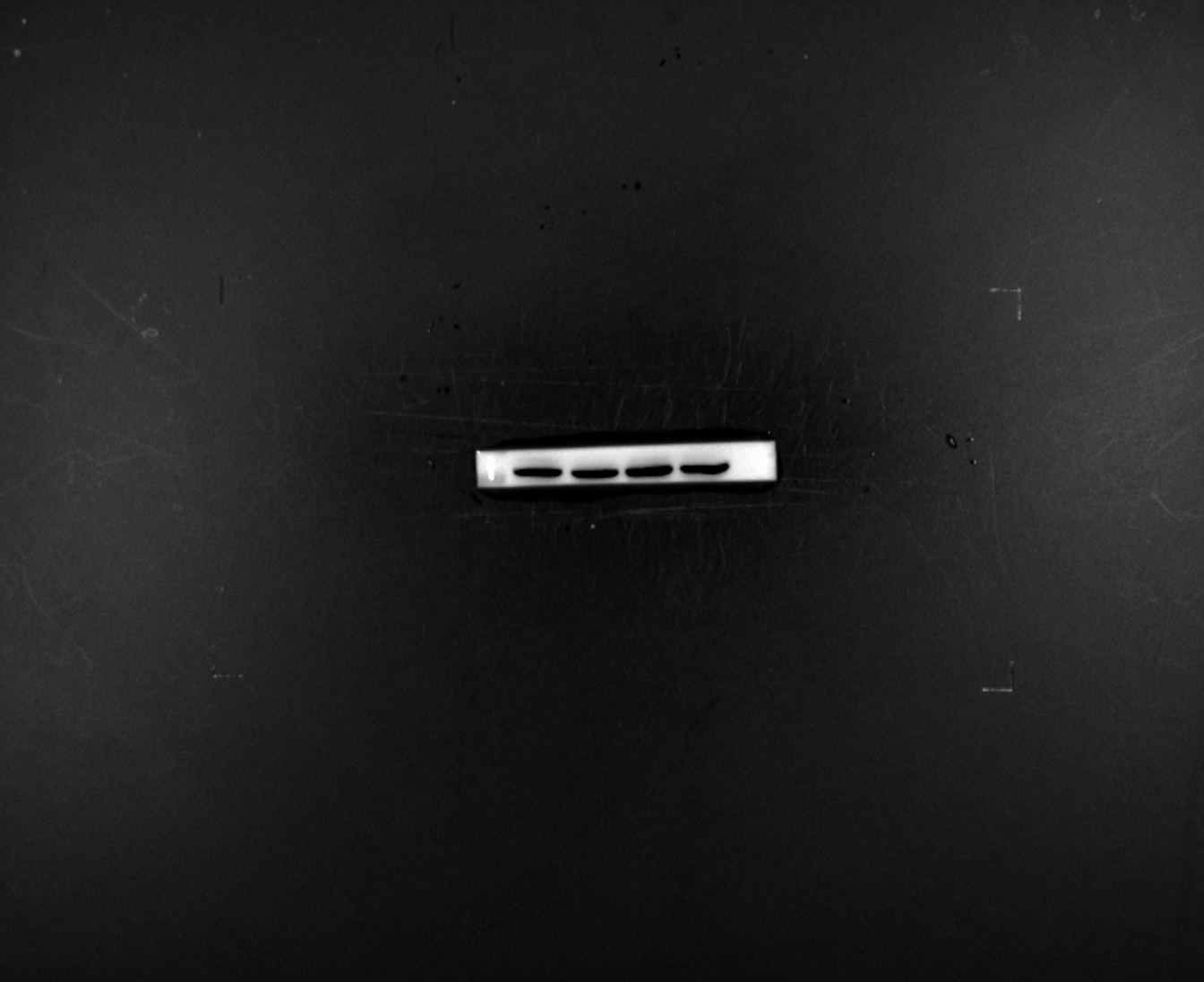


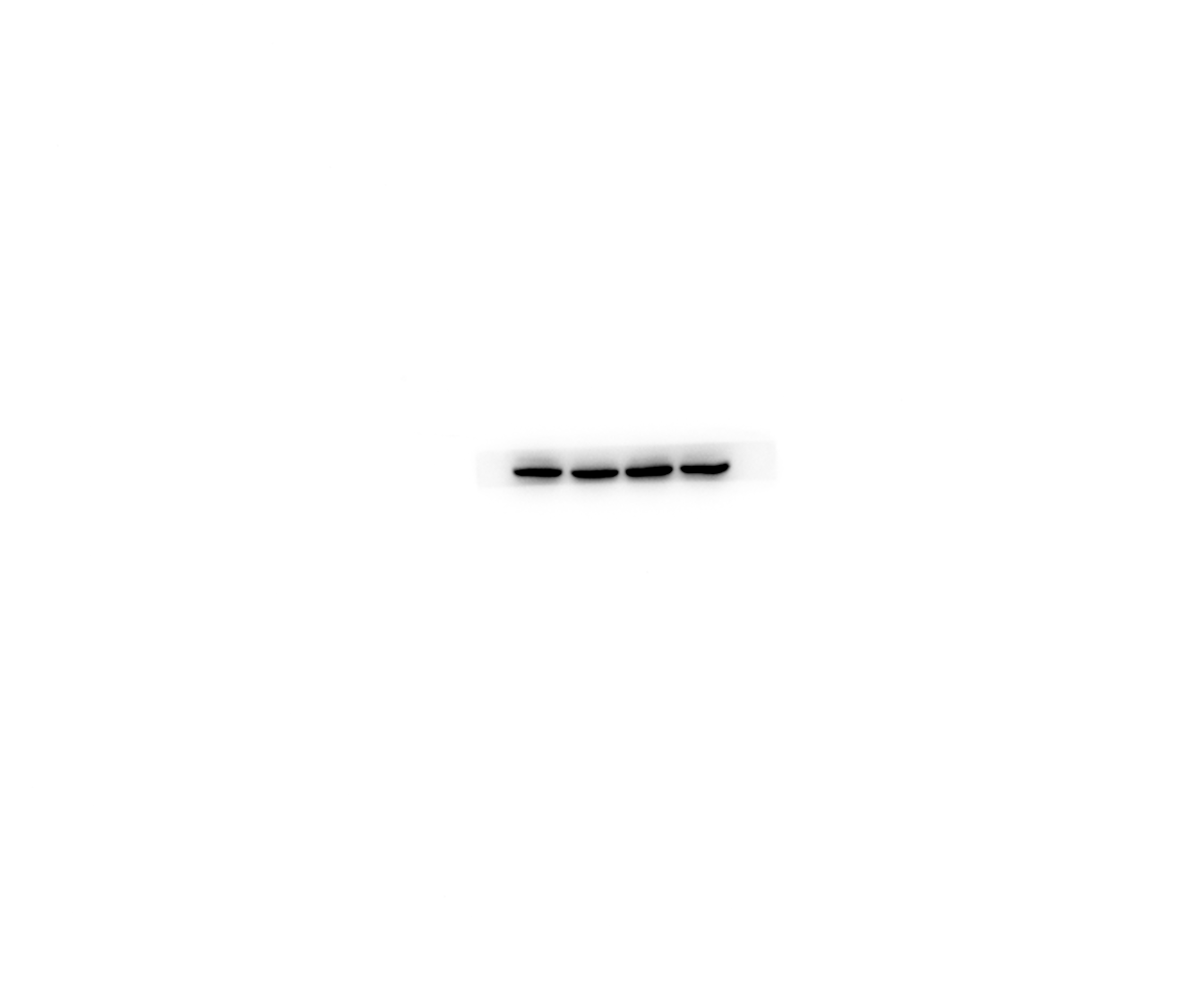


highest quality resolution microscopy images

Fig 6D


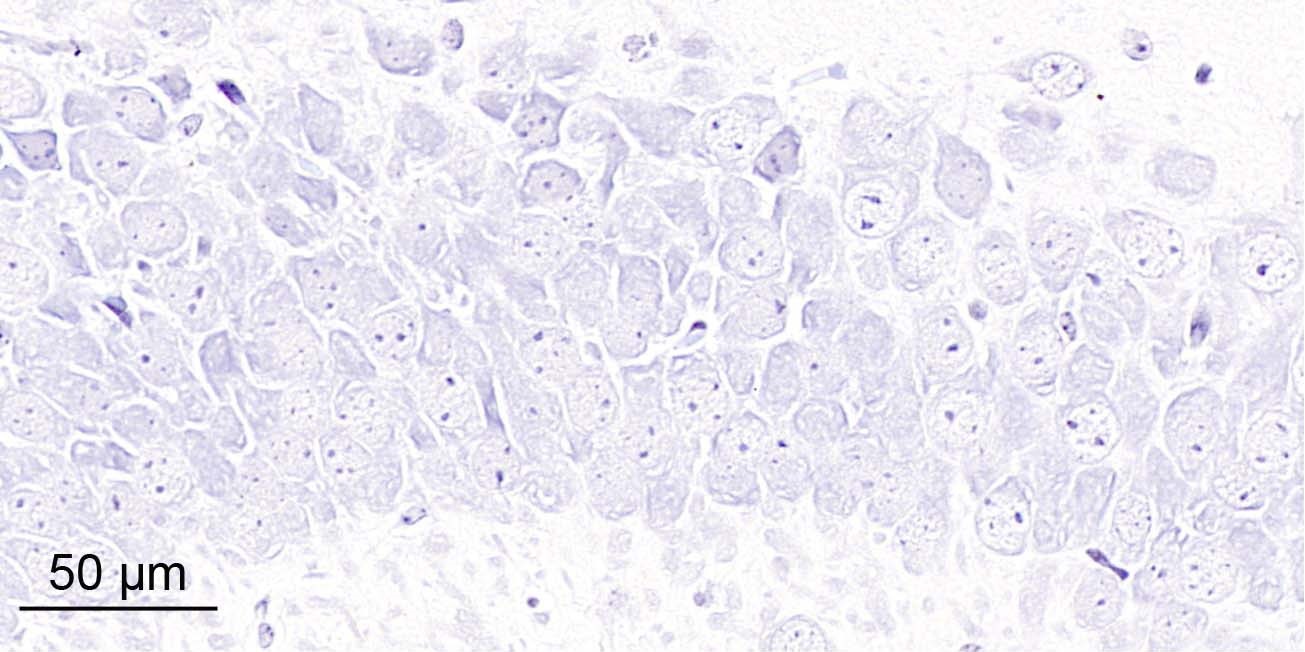

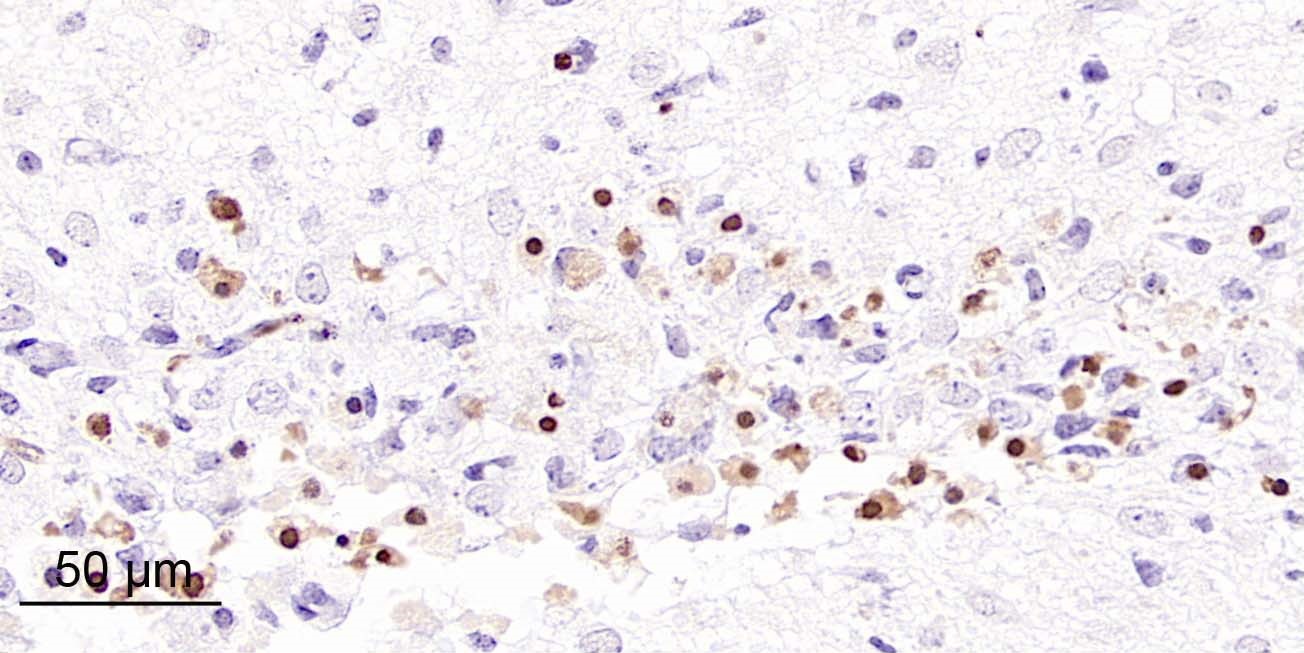

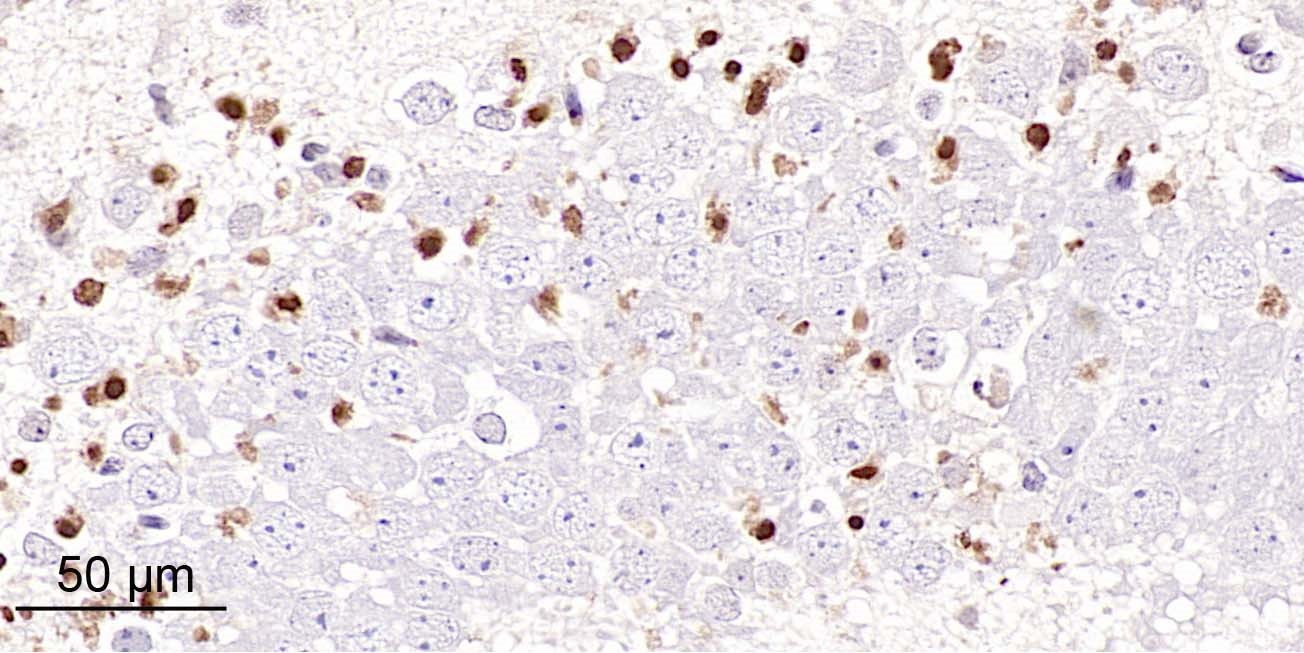

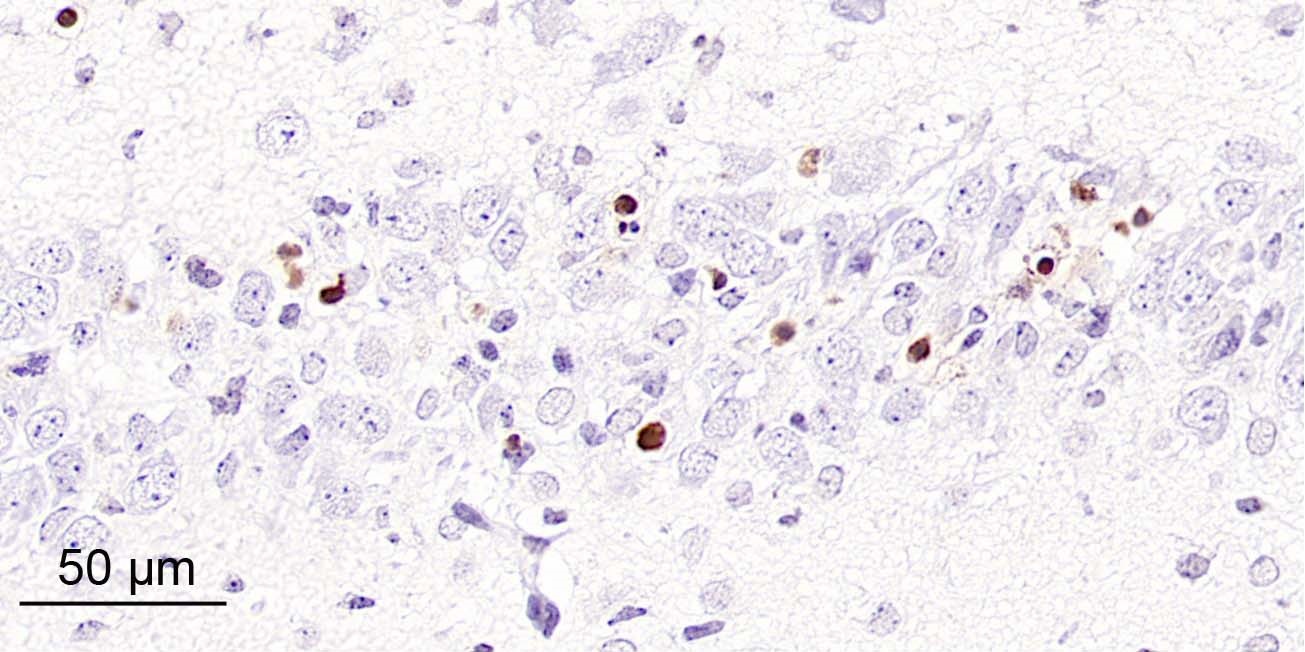

Supplement: Supplemental Material [file KBIE_A_2074738_SM0221.zip › supplementary/western blot images and highest quality resolution microscopy images.docx]
